# Genetic association of telomere length, obesity and tobacoo smoking with idiopathic pulmonary fibrosis risk

**DOI:** 10.1186/s12889-023-15733-5

**Published:** 2023-05-11

**Authors:** Wenjuan Wu, Chenghai Li, Xiaoming Zhu, Xueya Liu, Ping Li, Ruijie Wan, Xinhui Wu, Song Chen

**Affiliations:** 1 Department of Geriatrics Medicine, Henan Provincial People’s Hospital, Zhengzhou University, Zhengzhou, China; 2 Stem cell program of clinical research center, People’s Hospital of Zhengzhou University and Henan Provincial People’s Hospital, Zhengzhou, China; 3 Department of Thoracic Surgery, Henan Provincial People’s Hospital, Zhengzhou, China; 4 Department of Geriatrics Medicine, Henan Provincial People’s Hospital, Zhengzhou, China; 5 Department of Respiratory and Critical Care Medicine, The First Affiliated Hospital of Zhengzhou University, Zhengzhou, China; 6 Department of Geriatrics Medicine, Henan Provincial People’s Hospital, Zhengzhou, China; 7 Department of Traditional Chinese Medicine, Zhengzhou Shuqing Medical College, Zhengzhou, China; 8 Translational Research Institute, Henan Provincial People’s Hospital, Academy of Medical Science, Zhengzhou University, Zhengzhou, China

**Keywords:** Idiopathic pulmonary fibrosis, Mendelian randomization, Obesity, Telomere length, Tobacco smoking

## Abstract

**Background:**

Due to the inadequacy of published evidence, association of telomere length (TL), obesity and tobacco smoking with idiopathic pulmonary fibrosis (IPF) remains unclear. The aim of the study was to explore whether these exposures genetically affected the risk of the disease.

**Methods:**

Genetic variants from genome-wide association studies for TL, body mass index (BMI), body fat percentage (BFP) and tobacco smoking (including maternal smoking) were used as instrumental variables. Inverse-variance weighted were mainly adopted to determine the genetic association of these exposures with IPF. All analyses were conducted by R-software (version 3.6.1).

**Results:**

Firstly, longer TL was associated with the decreased risk of IPF (OR = 0.475 per SD increase in TL, 95%CI = 0.336 ~ 0.670, P<0.001). Secondly, higher levels of BMI and BFP were related to the increased risk of the disease (OR = 1.425 per SD increase in BMI level, 95%CI = 1.114 ~ 1.823, P = 0.005; OR = 1.702 per SD increase in BFP level, 95%CI = 1.202 ~ 2.409, P = 0.003). Thirdly, maternal smoking was implicated in the increased risk of the disease (OR = 13.183 per SD increase in the prevalence of maternal smoking, 95%CI = 1.820 ~ 95.484, P = 0.011).

**Conclusion:**

TL should be a genetic risk factor for IPF. Obesity and exposure to tobacco smoking as a fetus might also contribute to the development of this fibrotic diseases. These findings should be verified by future studies.

**Supplementary Information:**

The online version contains supplementary material available at 10.1186/s12889-023-15733-5.

## Introduction

Idiopathic pulmonary fibrosis (IPF) is a chronic, progressive interstitial lung disease that tends to occur in middle-aged and elderly people [1]. According to statistics, the prevalence rate of the disease in the global population is about 2 to 29 per 100,000 each year [2]. In China, the number of IPF patients is conservatively estimated at least about 500,000 [3, 4]. More importantly, IFP is a fatal disorder with unsatisfactory prognosis [5–7]. So, exploring the risk factors and pathogenesis of the disease is of great significance for the control of the disease, and related research is being carried out.

Telomere length (TL) is a biomarker closely related to aging [8]. Previous observational studies suggested that shortening TL predicted the increased risk and poor prognosis of pulmonary fibrosis [9, 10]. One Mendelian randomization (MR) study which adopted genetic variants as proxies for exposure and outcome reported a casual relationship between TL and IPF for the first time [11]. However, this can only be regarded as a preliminary conclusion due to the small sample size of IPF and insufficient power of test, and needed to be validated by further research.

As we all know, a great number of factors have been shown to potentially affect TL. One recent systematic review and meta-analysis including 84 studies suggested that TL was shorter among ever-smokers compared to never-smokers, which may imply mechanisms linking tobacco smoking to ageing-related disease [12]. One published MR study revealed that higher genetically-predicted alcohol use disorder was associated with shorter TL, and non-linear analyses indicated a potential threshold relationship between alcohol and TL [13]. Two meta-analyses also revealed the possibility of an effect of paediatric obesity on telomerase length, although the current evidence had not been sufficient yet [14, 15].

Importantly, an increasing number of observational studies focused on the potential effects of these telomere-associated factors (i.e. tobacco smoking, alcohol drinking and obesity) on the risk of IPF, and provided some interesting findings [9, 10]. Considering the limitations of observational studies, these findings need to be validated at the genetic level by MR analysis.

Taken together, using the most updated and available genome-wide association study (GWAS) data for IPF, TL and several telomere-associated factors (i.e. tobacco smoking, alcohol drinking and obesity), the present MR study aimed to explore and verify the genetic effects of TL and these telomere-associated factors on the risk of IPF, so as to provide new knowledge about the pathogenesis of this fibrotic disease. In this process, we adopted heavy smoking, smoking initiation, age of smoking initiation, smoking cessation and maternal smoking to interpret different stages and levels of tobacco exposure, and also used body mass index (BMI) and body fat percentage (BFP) to more comprehensively describe obesity and fat accumulation.

## Methods

**Fig. 1:**
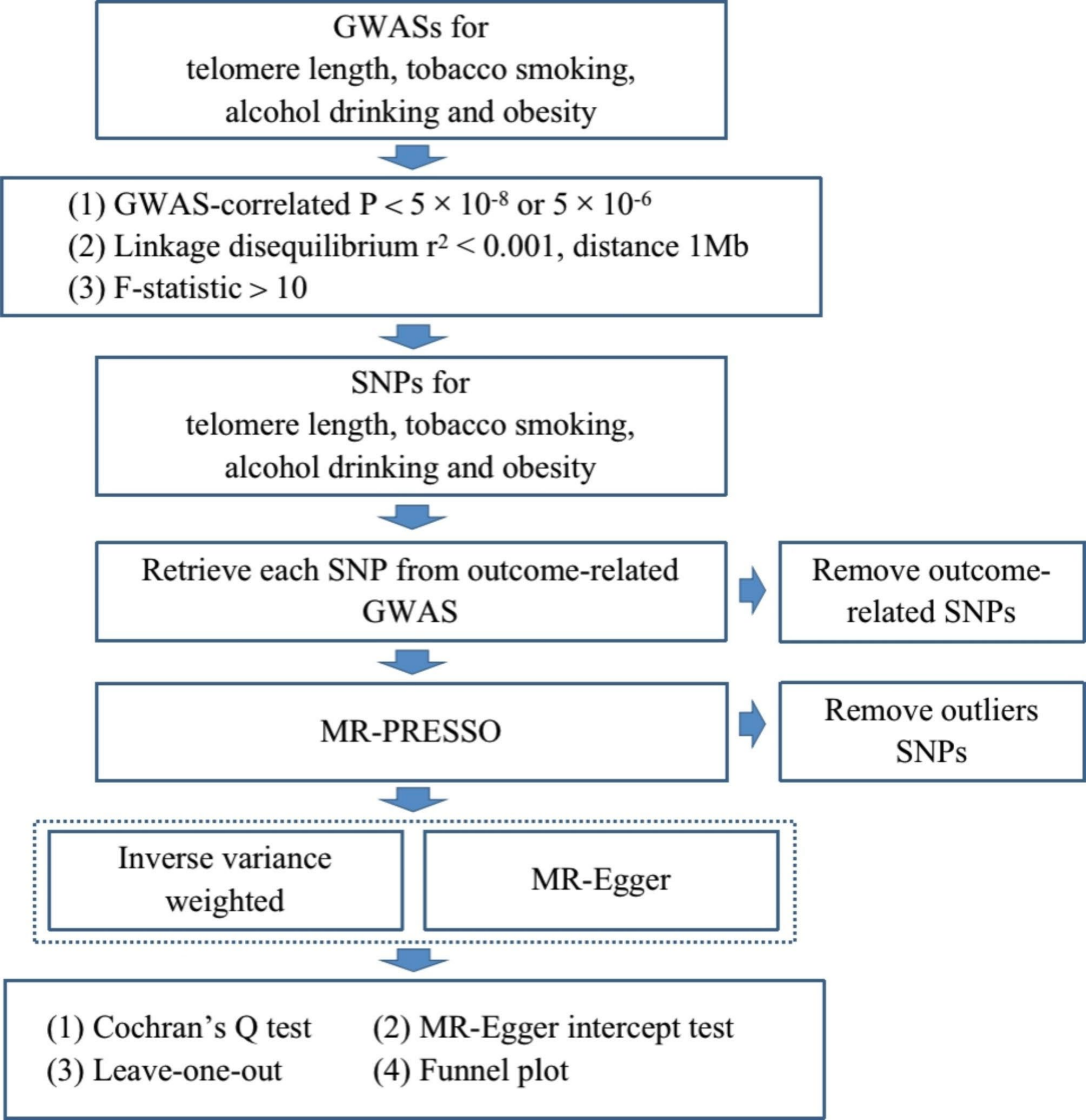
Diagram of Mendelian randomization framework in the study

Diagram of MR framework in the study was showed in Fig. 1.

### Data sources for the exposures

The summary data for TL was obtained from a published GWAS which characterized the genetic architecture of naturally occurring variation in leukocyte TL using quantitative PCR assay and identified causal links between TL and biomedical phenotypes in 472,174 well-characterized UK Biobank participants [16]. An increase or decrease in TL indicated an increase or decrease of one or more standard deviations (SDs) in the mean base pairs.

The data for heavy smoking, smoking initiation, age of smoking initiation, smoking cessation, heavy drinking was collected from a GWAS and Sequencing Consortium of Alcohol and Nicotine use (GSCAN) study which explored the genetic etiology of tobacco and alcohol use in up to 1.2 million European participants [17]. Heavy smoking and heavy drinking were defined as daily tobacco use and weekly alcohol use respectively. Smoking initiation was a binary phenotype indicating whether an individual had ever smoked regularly, while smoking cessation was also a binary variable contrasting current versus former smokers [17].

The data for maternal smoking and BFP was included from studies of MRC Integrative Epidemiology Unit (MRC-IEU) using 397,732 to 454,633 UK Biobank participants from IEU Open GWAS Project (https://gwas.mrcieu.ac.uk/), and the IDs were separately ukb-b-17,685 and ukb-b-8909. Maternal smoking was defined as maternal tobacco smoking around birth. The data for BMI was collected from a meta-analysis of Genetic Investigation of ANthropometric Traits (GIANT) studies for the trait in up to 700,000 participants of European ancestry [18].

Characteristics of these exposures were shown in Table 1.

**Table 1:** Sources and characteristics of GWAS summary data in the study

Phenotype	Data source	Sample size	Characteristics of subjects	Unit of data	Inclusion criteria of SNPs	No. of SNPs
Telomere length	Codd et al. 2021 PMID: 34,611,362	472,174	European Males and Females Aged 40-69	SD	P < 5 × 10^-8^; r2=0.001, kb=1 M; F-statistic>10	96
Heavy smoking	Liu et al. 2019 PMID: 30,643,251 GSCAN ^a^	337,334	European Males and Females Any age	SD	P < 5 × 10^-8^; r2=0.001, kb=1 M; F-statistic>10	19
Smoking initiation	Liu et al. 2019 PMID: 30,643,251 GSCAN	1,232,091	European Males and Females Any age	Log odds	P < 5 × 10^-8^; r2=0.001, kb=1 M; F-statistic>10	77
Age of smoking initiation	Liu et al. 2019 PMID: 30,643,251 GSCAN	341,427	European Males and Females Any age	SD	P < 5 × 10^-6^; r2=0.001, kb=1 M; F-statistic>10	20
Smoking cessation	Liu et al. 2019 PMID: 30,643,251 GSCAN	547,219	European Males and Females Any age	Log odds	P < 5 × 10^-6^; r2=0.001, kb=1 M; F-statistic>10	19
Maternal smoking	Ben Elsworth 2018 ID: ukb-b-17,685 MRC-IEU	397,732	European Males and Females Any age	SD	P < 5 × 10^-6^; r2=0.001, kb=1 M; F-statistic>10	50
Heavy drinking	Liu et al. 2019 PMID: 30,643,251 GSCAN	941,280	European Males and Females Any age	SD	P < 5 × 10^-8^; r2=0.001, kb=1 M; F-statistic>10	29
Body mass index	Yengo et al. 2018 PMID: 30,124,842 GIANT	681,275	European Males and Females Any age	SD	P < 5 × 10^-8^; r2=0.001, kb=1 M; F-statistic>10	443
Body fat percentage	Ben Elsworth 2018 ID: ukb-b-8909 MRC-IEU	454,633	European Males and Females Any age	SD	P < 5 × 10^-8^; r2=0.001, kb=1 M; F-statistic>10	315
IPF	Kurki et al. 2022 FinnGen	340,596	European Males and Females Mean age: 69.80	SD	NA	NA

^a^ GSCAN = GWAS and Sequencing Consortium of Alcohol and Nicotine use; GIANT = Genetic Investigation of ANthropometric Traits; MRC-IEU = MRC Integrative Epidemiology Unit; IPF = Idiopathic pulmonary fibrosis; SD = Standard deviation

### Data source for the outcome

The summary data for IPF was obtained from an available and updated FinnGen database (DF8) including 340,596 European participants with 1,812 cases and 338,784 controls [19]. The outcome was searched from hospital discharge records or death records using International Classification of Diseases 10^th^ edition (ICD−10) codes (ICD−10 code: J84.1). Characteristics of the outcome were shown in Table 1.

### Selection of genetic instruments

Single nucleotide polymorphisms (SNPs) are one of the most common types of genetic variants in humans and are used as instrumental variables to replace traits in MR studies [20]. The suitable SNPs were extracted from the above exposure-associated GWASs according to the following criteria: (1) A genome-wide significance-associated P value was less than 5 × 10^− 8^. If the number of available SNPs was insufficient, a more relaxed threshold (5 × 10^− 6^) was adopted. (2) A F-statistic should be higher than 10. (3) Remove SNPs with linkage disequilibrium [LD] (a clumping window of 10 MB and a r^2^ value less than 0.001). (4) All SNPs associated with the outcome and potential confounders were directly excluded using the PhenoScanner Pheno Scanner V2, and these confounders included air pollution, viral infection, autoimmune deficiency and gastroesophageal reflux disease [21].

### Mendelian randomization analysis

Random-effect inverse-variance weighted (IVW) and MR-Egger were adopted to determine the causal association of TL and the associated factors with the risk of IPF. The IVW result is the slope of a weighted regression of SNP-outcome effect on SNP-exposure effect with an intercept equal to zero [22]. In MR studies, the intercept is the longitudinal coordinate of the line as it crosses the Y-axis. While the x-axis represents the SNP-exposure effect and the y-perimeter represents the SNP-outcome effect in a scatter plot, an intercept of zero means that when the SNP-exposure effect is zero, the SNP-outcome effect is also zero. It is widely accepted that the IVW is the most reliable method compared to the other methods. The MR-Egger complements the IVW, as the MR-Egger can be applied to a wider range of conditions and produce more conservative results, but is less efficient [23]. All instrumental variables in MR-Egger can have pleiotropy, but these pleiotropies cannot affect the association of instrumental variables with exposure. Here, pleiotropy refers primarily to horizontal pleiotropy, meaning that one genetic variant as an instrumental variable also affects outcome through other pathways independent of the exposure [24]. In the study, the correlation was considered statistically significant when the IVW P-value was less than 0.05 and the MR-Egger results were at least in the same direction as the IVW results [25]. In addition, scatter plots and forest plots were used to visualize the results obtained from the IVW and MR-Egger methods.

Four main methods were adopted for sensitivity analysis to control the quality of data and results. (1) Cochran’s Q test was adopted to determine underlying heterogeneity. (2) MR-Egger intercept test was used to measure horizontal pleiotropy. (3) Leave-one-out test evaluated whether the estimate was led by one single SNP, which was also related to pleiotropy. (4) Funnel plot assessed directional pleiotropy, which was similar to publication bias in meta-analysis. In MR studies, directional pleiotropy mainly refers to horizontal pleiotropy.

In addition, MR-Pleiotropy Residual Sum and Outlier method (MR-PRESSO) was also adopted to find outliers and correct horizontal pleiotropy. The method can also provide causal estimates including and removing outiers. MR-PRESSO is more accurate than the IVW and MR-Egger, when the number of genetic variants with horizontal pleiotropy is less than 10% of the total [24].

All analyses were conducted using TwoSampleMR package (version 0.4.25) and MRPRESSO package (version 1.0) in R software (version 3.6.1).

## Results

### Overview of the analysis

**Fig. 2:**
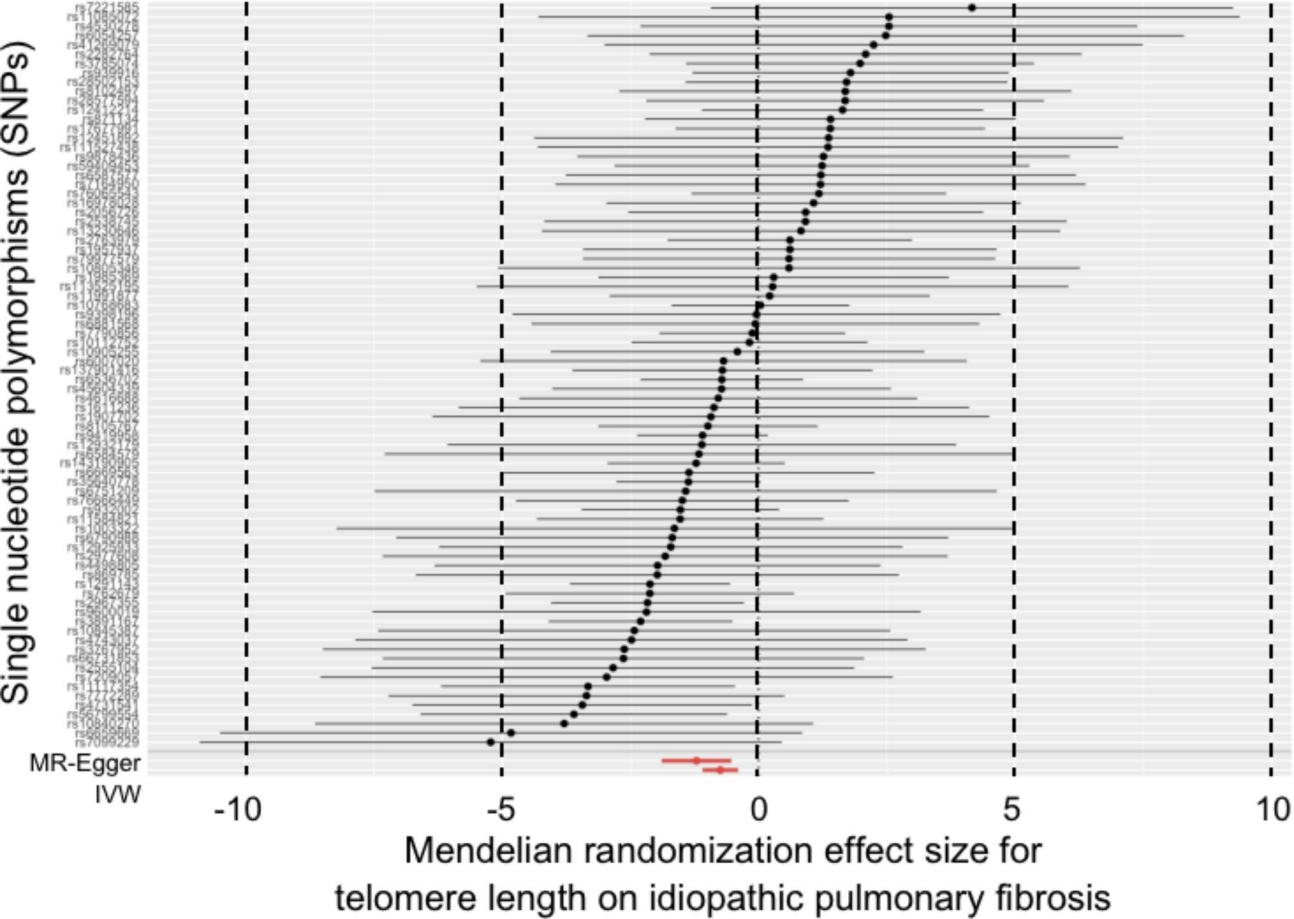
Mendelian randomization effect of telomere length on idiopathic pulmonary fibrosis risk (excluding the SNPs associated with smoking or obesity)

**Fig. 3:**
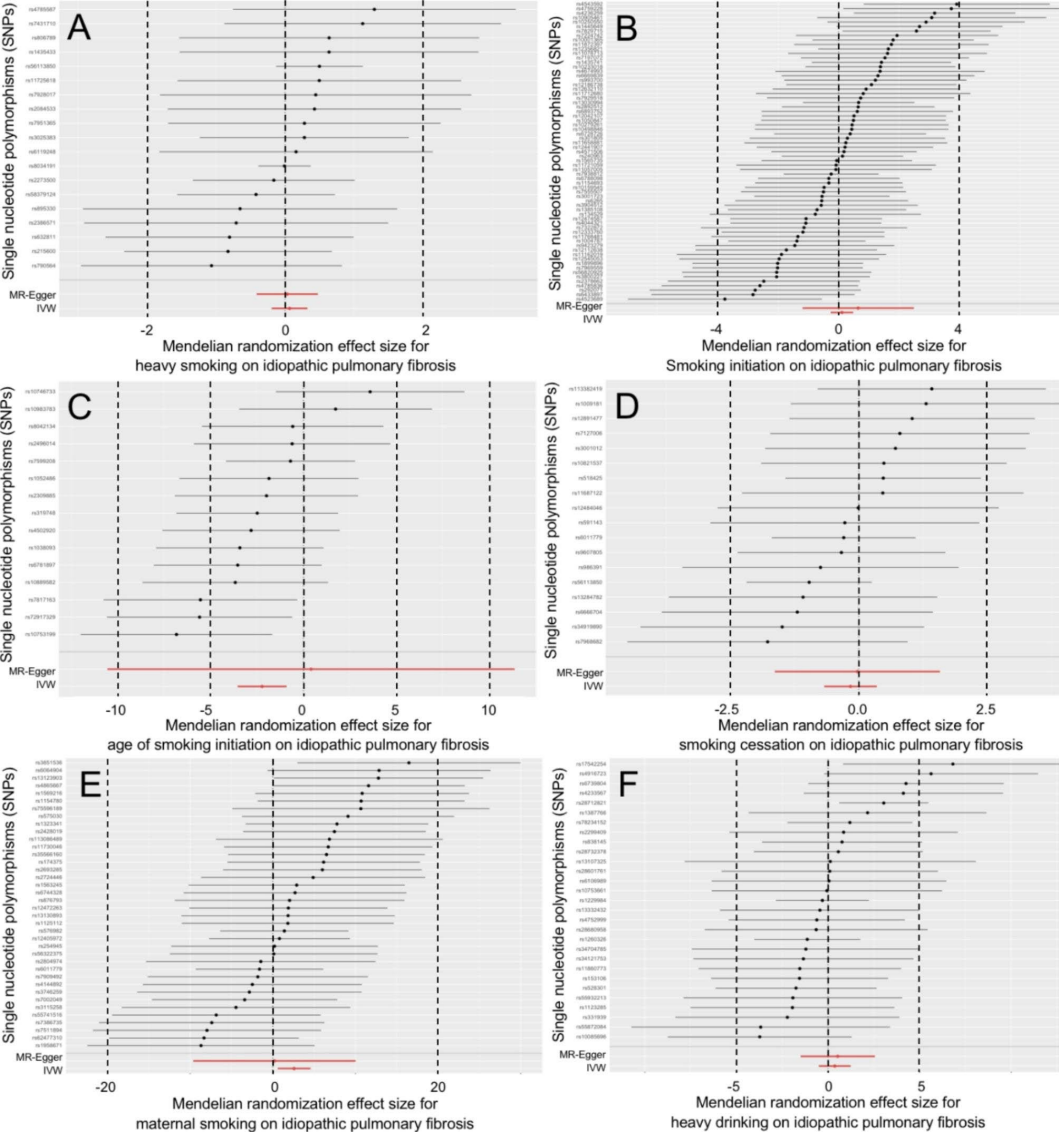
Forest plots for effect of tobacco smoking and alcohol drinking on idiopathic pulmonary fibrosis risk (excluding the SNPs associated with telomere length or obesity in Figure 3C and 3E)

**Fig. 4:**
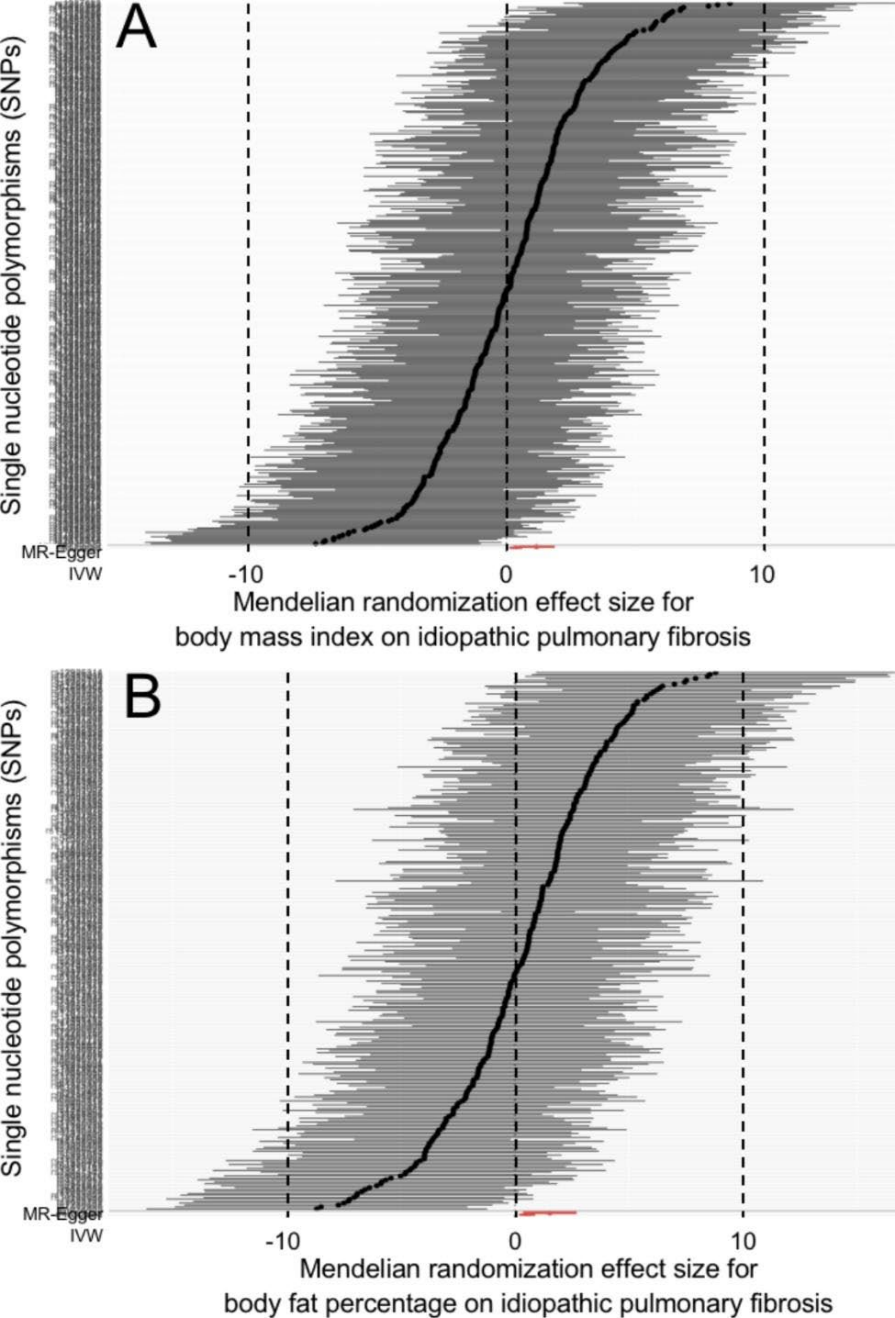
Mendelian randomization effect of body mass index and body fat percentage on idiopathic pulmonary fibrosis risk (excluding the SNPs associated with telomere length or smoking)

In Table 1, a total of 19 to 443 SNPs were separately extracted to genetically predict TL and its 8 associated factors. All SNPs met the inclusion criteria mentioned in Table 1, and were listed in supplemental Table 1 . The IVW and MR-Egger results as well as the Cochran’s Q and MR-Egger intercept results were showed in Table 2. Total R^2^, F-statistic and test power for each estimate was listed in supplemental Table 1 . MR-PRESSO results were showed in supplemental Table 3. The scatter plots, forest plots, Leave-one-out tests and funnel plots were showed in Figs. 2, 3 and 4 and supplemental Fig. 1 to 9 .

**Table 2:** Effect of telomere length and associated factors on idiopathic pulmonary fibrosis risk

Exposures	Modes	No. of SNPs ^a^	P value for MR	OR (95%CI)	P value for Cochran’s Q	P value for MR-Egger intercept
Telomere						
Telomere length	IVW	96	<0.001	0.459 (0.327~0.643)	0.384	0.110
	MR-Egger	96	<0.001	0.284 (0.145~0.556)		
Telomere length ^b^	IVW	85	<0.001	0.475 (0.336~0.670)	0.665	0.121
	MR-Egger	85	<0.001	0.298 (0.152~0.586)		
Tobacco smoking						
Heavy smoking	IVW	19	0.627	1.066 (0.823~1.382)	0.903	0.860
	MR-Egger	19	0.891	1.032 (0.663~1.607)		
Smoking initiation	IVW	77	0.571	1.113 (0.769~1.611)	0.155	0.561
	MR-Egger	77	0.495	1.905 (0.303~11.987)		
Age of initiation	IVW	20	0.001	0.136 (0.040~0.464)	0.134	0.104
	MR-Egger	20	0.251	102.692 (0.049~2.136e+05)		
Age of initiation ^c^	IVW	16	0.001	0.108 (0.029~0.402)	0.300	0.643
	MR-Egger	16	0.943	1.505 (2.592e-05~8.742e+04)		
Smoking cessation	IVW	19	0.540	0.852 (0.511~1.422)	0.841	0.865
	MR-Egger	19	0.975	0.974 (0.195~4.860)		
Maternal smoking	IVW	50	0.002	15.487 (2.679~89.543)	0.404	0.717
	MR-Egger	50	0.812	2.995 (0.001~2.446e+04)		
Maternal smoking ^c^	IVW	40	0.011	13.183 (1.820~95.484)	0.421	0.631
	MR-Egger	40	0.968	1.228 (6.682e-05~2.256e+04)		
Alcohol drinking						
Heavy drinking	IVW	29	0.405	1.446 (0.607~3.442)	0.542	0.861
	MR-Egger	29	0.610	1.704 (0.226~12.874)		
Obesity						
Body mass index	IVW	443	0.005	1.386 (1.106~1.737)	0.264	0.069
	MR-Egger	443	0.006	2.354 (1.276~4.340)		
Body mass index ^d^	IVW	403	0.005	1.425 (1.114~1.823)	0.201	0.164
	MR-Egger	403	0.001	3.200 (1.586~6.455)		
Body fat percentage	IVW	315	0.017	1.502 (1.075~2.097)	0.090	0.130
	MR-Egger	315	0.031	3.384 (1.127~10.163)		
Body fat percentage ^d^	IVW	294	0.003	1.702 (1.202~2.409)	0.143	0.083
	MR-Egger	294	0.011	4.603 (1.424~14.880)		

^a^ IVW = Inverse variance weighted; SNP = SNP = Single nucleotide polymorphism; OR = Odds ratio; 95%CI = 95% Confidence interval; P value for MR = P value for Mendelian randomization

^b^ The SNPs associated with smoking or obesity were excluded. ^c^ The SNPs associated with telomere length or obesity were excluded. ^d^ The SNPs associated with telomere length or smoking were excluded

### Effect of TL on the risk of IPF

Both IVW and MR-Egger results reported that longer TL was associated with the decreased risk of IPF (OR = 0.459 per SD increase in TL, 95%CI = 0.327 ~ 0.643, P<0.001; OR = 0.284 per SD increase in TL, 95%CI = 0.145 ~ 0.556, P<0.001). Pre-analyses showed that some markers of obesity and smoking can also affect the risk of IPF. Therefore, after excluding the obesity and smoking-related SNPs, the analysis was re-conducted. The updated IVW and MR-Egger results still reported that longer TL was associated with the decreased risk of the disease (OR = 0.475 per SD increase in TL, 95%CI = 0.336 ~ 0.670, P<0.001; OR = 0.298 per SD increase in TL, 95%CI = 0.152 ~ 0.586, P<0.001). These results were visualized in Fig. 2 and supplemental Fig. 1 .

For the updated results, P value for Cochran’s Q was 0.665 and P value for MR-Egger intercept was 0.121, which indicated that there was no significant heterogeneity and horizontal pleiotropy (Table 2). The “leave-one-SNP-out” analysis and funnel plot also found no horizontal pleiotropy and bias in the analysis (supplemental Fig. 1 ). The MR-PRESSO did not detect any outliers (P = 0.658), and its causal estimate was consistent with the results from the IVW and MR-Egger (P < 0.001) (supplemental Table 3 ).

These findings indicated that the risk of IPF was reduced by approximately 50–70% for each SD increase in TL.

### Effect of tobacco smoking and alcohol drinking on the risk of IPF

Five variables (i.e. heavy smoking, smoking initiation, age of smoking initiation, smoking cessation and maternal smoking) were used to represent tobacco smoking in a multifaceted way. Heavy drinking was adopted to represent alcohol drinking. Their definitions had been mentioned above.

The IVW results revealed that older age of smoking initiation was associated with the decreased risk of IPF (OR = 0.136 per SD increase in age of smoking initiation, 95%CI = 0.040 ~ 0.464, P = 0.001). However, the MR-Egger results were not statistically significant and were in the opposite direction (OR = 102.692 per SD increase in age of smoking initiation, 95%CI = 0.049 ~ 2.136e + 05, P = 0.251). These results were showed in Table 2.

The IVW results revealed that maternal smoking was associated with the increased risk of the disease (OR = 15.487 per SD increase in the prevalence of maternal smoking, 95%CI = 2.679 ~ 89.543, P = 0.002). And, the MR-Egger results were in the same direction as the IVW results (OR = 2.995 per SD increase in the prevalence of maternal smoking, 95%CI = 0.001 ~ 2.446e + 04, P = 0.812). After removing the TL and obesity-related SNPs, the updated results from the IVW and MR-Egger did not change significantly (OR = 13.183 per SD increase in the prevalence of maternal smoking, 95%CI = 1.820 ~ 95.484, P = 0.011; OR = 1.228 per SD increase in the prevalence of maternal smoking, 95%CI = 6.682e−05 ~ 2.256e + 04, P = 0.968). These results were showed in Table 2, and were also visualized in Fig. 3, supplemental Fig. 6 .

For the updated results of the maternal smoking, the Cochran’s Q, MR-Egger intercept, “leave-one-SNP-out” analysis and funnel plot did not find any heterogeneity and horizontal pleiotropy (Table 2, supplemental Fig. 6 ). The MR-PRESSO reported no significant outliers (P = 0.451), and its causal estimates were consistent with the results from the IVW (P = 0.015) (supplemental Table 3 ).

In addition, the study did not find any association of heavy smoking, smoking initiation, smoking cessation and heavy drinking with the risk of IPF.

These findings indicated that for every SD increase in the prevalence of maternal smoking, the risk of IPF was likely to increase more than tenfold. Given the wide 95%CIs, these results should be further verified.

### Effect of obesity on the risk of IPF

BMI and BFP were used as the markers of obesity in the study. After removing the TL and smoking-associated SNPs, the MR-Egger method reported that higher levels of BMI and BFP were associated with the increased risk of IPF (OR = 1.425 per SD increase in BMI level, 95%CI = 1.114 ~ 1.823, P = 0.005; OR = 1.702 per SD increase in BFP level, 95%CI = 1.202 ~ 2.409, P = 0.003). The MR-Egger reported similar results (OR = 3.200 per SD increase in BMI level, 95%CI = 1.586 ~ 6.455, P = 0.001; OR = 4.603 per SD increase in BFP level, 95%CI = 1.424 ~ 14.880, P = 0.011). The results were showed in Table 2; Fig. 4, supplemental Figs. 8 and 9 .

All P values for Cochran’s Q and MR-Egger intercept were larger than 0.05 (Table 2). The “leave-one-SNP-out” analyses and funnel plots did not find horizontal pleiotropy and bias in the analysis (supplemental Figs. 8 and 9 ). The MR-PRESSO did not detect any outliers (P = 0.200, P = 0.153), and their causal estimates also reported the causal association of BMI and BFP with the disease (P = 0.005, P = 0.003) (supplemental Table 3 ).

These findings indicated that the risk of IPF was increased by approximately 40–70% for each SD increase in BMI or BFP level.

## Discussion

As mentioned above, one previous MR study had preliminarily reported a causal relationship between TL and the risk of IPF [11]. However, all current summary data on IPF (including the data used in the above study) were limited by small sample sizes and cannot be resolved in the short term. Therefore, iterative validation of this correlation was necessary. The present study adopted another set of summary data for the fibrotic disease, and provided a result that TL was associated with the risk of IPF (Detailed data was listed in the result section). This finding was consistent with that previous study, and further confirmed the role of telomere in the pathogenesis of IPF.

The study explored the causal relationship between several types of smoking history and the risk of IPF. Although most correlations were not statistically significant, there were still some interesting findings that may be useful for future studies. Specifically, at least in the IVW results, participants whose mothers had maternal smoking may be subject to a higher risk of the disease (Detailed data was listed in the result section). This finding was supported by the MR-PRESSO, and the MR-Egger result also had a same direction as the IVW result. These results were consistent with one important medical truism that many organs of the fetus (including the lungs) were at a developmental stage and were susceptible to damage from tobacco. In addition, genetic mechanisms were thought to play an important role in the pathogenesis of IPF, which partly supported the results in the study [26].

The study also found that obesity may significantly increase the risk of developing IPF (Detailed data was listed in the result section). A recent meta-analysis showed that BMI might be useful to predict mortality, disease progression and treatment-related toxicity in IPF [27]. Another MR study showed that gastro-esophageal reflux disease may increase the risk of IPF, but after adjustment for BMI, these associations were significantly attenuated. Therefore, the investigators concluded that treating obesity, but not gastro-esophageal reflux disease, was likely to have a protective effect against IPF [28]. So, these results were consistent with the present study.

In two-sample MR studies, sample overlap can lead to biased effect estimates [29]. However, in some cases, sample overlap is difficult to avoid. Researchers can only assess the degree of sample overlap by calculating the overlap percentage, and control it by selecting other data. Thus, sample overlap is still one of the main potential limitations in this type of MR studies. In the present study, the summary data of the exposures were mainly from UK Biobank and other institutions, while the summary data of the outcome (i.e. IPF) came from FinnGen. Since UK Biobank and FinnGen were independent in summary data, we considered the degree of sample overlap in the present study to be acceptable and not to have an significant impact on the results.

At present, the sample sizes of all the summary data on IPF were relatively small, which may be related to the insidious onset of the disease and the difficulty in diagnosing it. In the present study, the most updated summary data for IPF had been selected, but the small sample size remained a limitation. This was a reasonable explanation for the very wide 95%CIs from some of the results in this study [30]. The powers of test had been calculated and listed them in the supplemental Table 2 . As the sample size of IPF data was limited by objective conditions, researchers can adopt summary data from different sources for the same trait to conduct their analyses and validate the results obtained against each other, which may be a realistic solution. However, it must be noted that MR can be difficult to interpret. Its methodology was extremely difficult to implement for non-specialists. Hence, only the researchers with the appropriate co-workers could conduct their analysis and validate the results.

In MR studies, weak instrumental variable bias is an issue that cannot be avoided. A weak instrumental variable is a genetic variant that is less potent in explaining exposure [31]. This instrument is associated with the exposure, but the strength of this association is not very high, so it is fundamentally different from the null instrumental variable. In general, the main cause of weak instrumental variable bias is inadequate sample size. In the present study, we calculated F-statistic, and any instrumental variable with an F-statistic less than 10 was excluded, thus reducing the impact of this bias on the study.

The present study performed several sensitivity analyses to detect and control the horizontal pleiotropy and heterogeneity, such as Cochran’s Q, MR-Egger intercept, and so on. These may help to ensure the reliability of the conclusions in this study. In addition, with the increasing size and number of GWASs, individual SNP is increasingly found to associate with multiple traits. Vertical pleiotropy is one of the scenarios, and it means that exposure is also associated with other traits, and association with outcome can be established through these traits [32]. Vertical pleiotropy is very common. One mainstream view is that vertical pleiotropy does not affect the results, and does not need to be accounted for in MR studies. However, this view is also not fully substantiated. So, sensitivity analyses were also necessary in the present study.

Potential effect of binary exposures on causal estimates could be a potential limitation in MR studies. When a binary exposure is a dichotomization of a continuous risk factor, its genetic variants (instrument variables) might violate the exclusion restriction assumption, because the genetic variants are able to affect the outcome through the continuous risk factor, though the binary exposure does not change [33]. In the present study, the exposures with potential positive results, such as TL, BMI, BFP and maternal smoking, were not classic binary exposures. So, the limitation may not affect our main conclusions. However, some of the other exposures in the study were binary exposures, the results of which may be affected by this limitation and need to be validated by further research.

Telomere length can decrease progressively with age. So, when exploring the effect of telomere length on one disease, the exposure and outcome should be from the same age group population. In the present study, although both sets of summary data (TL and IPF) were from the adult population, there was still a significant difference in age, which was an important limitation of the present study. Therefore, further research was necessary to explore this issue as the study data allowed.

In conclusion, the present study confirmed the causal association of TL with the risk of IPF. The present study also provided some interesting genetic evidence to prove that obesity and exposure to tobacco smoking as a fetus might also contribute to the development of the fibrotic diseases. However, it was important to note that the evidence on tobacco smoking exposure was inadequate and should be verified by future studies.

## Electronic supplementary material

Below is the link to the electronic supplementary material.

Supplementary Material 1

Supplementary Material 2

## Data Availability

Data was already available in the text and supplementary materials.

## List of abbreviations

IPF: Idiopathic pulmonary fibrosis
TL: Telomere length
MR: Mendelian randomization
GWAS: Genome-wide association study
BMI: Body mass index
BFP: Body fat percentage
GSCAN: GWAS and Sequencing Consortium of Alcohol and Nicotine use
MRC-IEU: MRC Integrative Epidemiology Unit
GIANT: Genetic Investigation of ANthropometric Traits
SNP: Single nucleotide polymorphisms
IVW: Inverse-variance weighted
MR-PRESSO: MR-Pleiotropy Residual Sum and Outlier method
