# Supplementary material for: Genetic association of telomere length, obesity and tobacoo smoking with idiopathic pulmonary fibrosis risk
Source: BMC Public Health. 2023 May 11;23:868. doi: 10.1186/s12889-023-15733-5 (PMC10176771; doi:10.1186/s12889-023-15733-5)
Supplement: Supplementary file 1 — Supplementary Material 1 [file 12889_2023_15733_MOESM1_ESM.docx]

**Supplemental Table 1 Exposure-associated SNPs in the study**

| **Exposures/No.** | **SNP ^a^** | **A1** | **A2** | **Eaf** | **Beta** | **SE** | **P** | **R^2^** | **F-statistic** |
| --- | --- | --- | --- | --- | --- | --- | --- | --- | --- |
| **Telomere length ^b^** |  |  |  |  |  |  |  |  |  |
| 1 | rs1003322 | A | C | 0.213742 | 0.0141734 | 0.00247546 | 1.00E-08 | 2.33E-05 | 11.01867817 |
| 2 | rs10112752 | A | G | 0.430369 | -0.0287522 | 0.00202518 | 9.50E-46 | 0.000209304 | 98.84815421 |
| 3 | rs10768683 | G | C | 0.841033 | 0.0469922 | 0.00277015 | 1.50E-64 | 0.000162964 | 76.95980069 |
| 4 | rs10805346 | C | T | 0.439339 | 0.0117072 | 0.00202147 | 7.00E-09 | 3.50E-05 | 16.52399037 |
| 5 | rs10840270 | G | C | 0.655684 | 0.014383 | 0.00212494 | 1.30E-11 | 4.38E-05 | 20.6873371 |
| 6 | rs10845387 | A | G | 0.352666 | -0.0141214 | 0.00209396 | 1.50E-11 | 4.40E-05 | 20.76624691 |
| 7 | rs10905255 | T | G | 0.57919 | -0.0182493 | 0.00203099 | 2.60E-19 | 8.34E-05 | 39.35938595 |
| 8 | rs11085072 | T | C | 0.236909 | -0.0131806 | 0.00236713 | 2.60E-08 | 2.37E-05 | 11.2104359 |
| 9 | rs11117354 | C | T | 0.696513 | 0.0232506 | 0.00219601 | 3.40E-26 | 0.000100368 | 47.39589946 |
| 10 | rs111527438 | C | T | 0.351251 | 0.0125 | 0.00211016 | 3.10E-09 | 3.39E-05 | 15.99286937 |
| 11 | rs113525195 | A | C | 0.290254 | -0.0124075 | 0.00224132 | 3.10E-08 | 2.67E-05 | 12.62646477 |
| 12 | rs11584821 | T | C | 0.176208 | -0.0306517 | 0.00263623 | 3.00E-31 | 8.31E-05 | 39.2509331 |
| 13 | rs11991877 | A | T | 0.889309 | -0.030138 | 0.00318686 | 3.20E-21 | 3.73E-05 | 17.60808948 |
| 14 | rs12412214 | A | G | 0.279765 | -0.0245174 | 0.00222685 | 3.40E-28 | 0.000103458 | 48.85483939 |
| 15 | rs12451892 | C | T | 0.380511 | -0.0116145 | 0.00207578 | 2.20E-08 | 3.13E-05 | 14.75981578 |
| 16 | rs1291143 | C | A | 0.849026 | 0.0493145 | 0.0027991 | 1.80E-69 | 0.000168525 | 79.58604866 |
| 17 | rs12925933 | C | A | 0.66221 | -0.0146622 | 0.00213796 | 7.00E-12 | 4.46E-05 | 21.04210388 |
| 18 | rs12932179 | G | A | 0.561399 | -0.0136257 | 0.0020276 | 1.80E-11 | 4.71E-05 | 22.24041796 |
| 19 | rs131797 | T | TAAAAA | 0.235625 | 0.0243752 | 0.00236561 | 6.80E-25 | 8.10E-05 | 38.24734177 |
| 20 | rs13230646 | C | T | 0.248945 | -0.0173277 | 0.00232377 | 8.90E-14 | 4.40E-05 | 20.79304003 |
| 21 | rs137901416 | A | G | 0.100311 | 0.04572 | 0.00332355 | 4.70E-43 | 7.23E-05 | 34.15926791 |
| 22 | rs143190905 | T | G | 0.080404 | -0.0723995 | 0.00369421 | 1.60E-85 | 0.00012029 | 56.80454739 |
| 23 | rs1611236 | A | G | 0.32687 | -0.0160135 | 0.00213359 | 6.10E-14 | 5.25E-05 | 24.78994483 |
| 24 | rs16978028 | T | A | 0.143727 | -0.029945 | 0.00285068 | 8.20E-26 | 5.75E-05 | 27.16160606 |
| 25 | rs17677991 | G | C | 0.342123 | 0.0222664 | 0.00210806 | 4.40E-26 | 0.000106363 | 50.22678777 |
| 26 | rs1907702 | A | G | 0.766771 | 0.0150247 | 0.00242651 | 5.90E-10 | 2.90E-05 | 13.71312534 |
| 27 | rs1957937 | T | A | 0.16018 | 0.0209365 | 0.00273361 | 1.90E-14 | 3.34E-05 | 15.78237028 |
| 28 | rs1985369 | G | A | 0.868178 | -0.0311893 | 0.00300952 | 3.60E-25 | 5.21E-05 | 24.58466126 |
| 29 | rs2056726 | A | G | 0.214376 | -0.0228078 | 0.00243638 | 7.90E-21 | 6.25E-05 | 29.52044865 |
| 30 | rs2276182 | G | C | 0.403227 | 0.0233529 | 0.00204247 | 2.80E-30 | 0.000133247 | 62.92381792 |
| 31 | rs2282764 | G | A | 0.142384 | -0.0224234 | 0.00289392 | 9.30E-15 | 3.11E-05 | 14.66309312 |
| 32 | rs2306646 | C | G | 0.559475 | -0.0209417 | 0.00201898 | 3.30E-25 | 0.000112315 | 53.03810705 |
| 33 | rs2538745 | C | T | 0.602841 | -0.012942 | 0.002056 | 3.10E-10 | 4.02E-05 | 18.97445753 |
| 34 | rs2555104 | C | A | 0.434255 | -0.0139717 | 0.00203498 | 6.60E-12 | 4.91E-05 | 23.16291565 |
| 35 | rs2763979 | T | C | 0.359721 | -0.0277713 | 0.00208092 | 1.30E-40 | 0.000173758 | 82.05788634 |
| 36 | rs28502153 | A | C | 0.377958 | -0.0215916 | 0.00206208 | 1.20E-25 | 0.000109182 | 51.55816581 |
| 37 | rs28577594 | C | G | 0.709829 | 0.0187657 | 0.00224024 | 5.40E-17 | 6.12E-05 | 28.90699396 |
| 38 | rs2967355 | C | A | 0.774276 | -0.0461595 | 0.00238972 | 4.00E-83 | 0.000276204 | 130.4517223 |
| 39 | rs2977608 | C | A | 0.743949 | 0.0129483 | 0.00233716 | 3.00E-08 | 2.48E-05 | 11.69384058 |
| 40 | rs35640778 | A | G | 0.020757 | -0.209011 | 0.00702087 | 9.59E-195 | 7.63E-05 | 36.0307066 |
| 41 | rs3767952 | A | G | 0.226709 | 0.0134472 | 0.00238826 | 1.80E-08 | 2.35E-05 | 11.11605318 |
| 42 | rs3785074 | G | A | 0.289672 | 0.023863 | 0.00220455 | 2.60E-27 | 0.000102118 | 48.22232391 |
| 43 | rs3891167 | G | A | 0.253435 | -0.0425685 | 0.00239551 | 1.20E-70 | 0.000253071 | 119.5234204 |
| 44 | rs41269079 | A | T | 0.188991 | 0.0153617 | 0.0025499 | 1.70E-09 | 2.36E-05 | 11.1259588 |
| 45 | rs4498805 | T | G | 0.546632 | 0.0150601 | 0.00200376 | 5.70E-14 | 5.93E-05 | 28.00039321 |
| 46 | rs4530278 | T | G | 0.59815 | 0.0138793 | 0.0020567 | 1.50E-11 | 4.64E-05 | 21.89352053 |
| 47 | rs45604339 | T | C | 0.34242 | -0.020433 | 0.00211433 | 4.30E-22 | 8.91E-05 | 42.06232622 |
| 48 | rs4616688 | T | G | 0.525394 | -0.0173476 | 0.00200198 | 4.50E-18 | 7.93E-05 | 37.44900326 |
| 49 | rs4731541 | G | C | 0.624901 | -0.0206119 | 0.00205962 | 1.40E-23 | 9.94E-05 | 46.95591652 |
| 50 | rs4743037 | T | C | 0.230874 | 0.0147971 | 0.00238094 | 5.10E-10 | 2.91E-05 | 13.71733068 |
| 51 | rs56178008 | A | T | 0.437497 | 0.0143739 | 0.00201464 | 9.70E-13 | 5.31E-05 | 25.05563748 |
| 52 | rs56799554 | G | A | 0.170183 | -0.0259793 | 0.00267858 | 3.00E-22 | 5.63E-05 | 26.57031748 |
| 53 | rs59409453 | G | A | 0.730602 | 0.0202133 | 0.00230175 | 1.60E-18 | 6.43E-05 | 30.35913845 |
| 54 | rs6007020 | C | T | 0.367823 | 0.0144904 | 0.00209637 | 4.80E-12 | 4.71E-05 | 22.22035755 |
| 55 | rs6054257 | A | G | 0.793522 | -0.0141684 | 0.00247729 | 1.10E-08 | 2.27E-05 | 10.7191091 |
| 56 | rs6536702 | A | G | 0.774647 | 0.0534148 | 0.00238875 | 9.40E-111 | 0.000369723 | 174.6376003 |
| 57 | rs6584579 | G | A | 0.398876 | 0.0114923 | 0.00204674 | 2.00E-08 | 3.20E-05 | 15.11933154 |
| 58 | rs6587577 | G | A | 0.826346 | -0.0182148 | 0.0026359 | 4.80E-12 | 2.90E-05 | 13.70498094 |
| 59 | rs6659669 | T | C | 0.605095 | -0.0117091 | 0.00205167 | 1.10E-08 | 3.30E-05 | 15.56648954 |
| 60 | rs6669563 | A | G | 0.437768 | 0.0182358 | 0.00202476 | 2.10E-19 | 8.46E-05 | 39.93254522 |
| 61 | rs66731853 | A | G | 0.317304 | -0.0177791 | 0.00215421 | 1.50E-16 | 6.25E-05 | 29.51220612 |
| 62 | rs670180 | A | T | 0.569104 | -0.0115801 | 0.00203062 | 1.20E-08 | 3.38E-05 | 15.95049732 |
| 63 | rs6751209 | C | T | 0.204231 | -0.0140465 | 0.00248465 | 1.60E-08 | 2.20E-05 | 10.38848454 |
| 64 | rs6790988 | G | A | 0.741902 | 0.0145728 | 0.00228428 | 1.80E-10 | 3.30E-05 | 15.58694518 |
| 65 | rs6881568 | A | C | 0.36258 | 0.0169256 | 0.00207735 | 3.70E-16 | 6.50E-05 | 30.68701945 |
| 66 | rs7099229 | A | G | 0.273301 | -0.0153288 | 0.00224403 | 8.40E-12 | 3.93E-05 | 18.53531564 |
| 67 | rs7164950 | G | A | 0.405979 | 0.0129362 | 0.00204001 | 2.30E-10 | 4.11E-05 | 19.39546577 |
| 68 | rs7209057 | A | G | 0.561044 | 0.011819 | 0.00202865 | 5.70E-09 | 3.54E-05 | 16.71893696 |
| 69 | rs7221585 | T | C | 0.22401 | 0.0143271 | 0.00247042 | 6.70E-09 | 2.48E-05 | 11.69330929 |
| 70 | rs76065543 | T | C | 0.137527 | 0.0342843 | 0.00290708 | 4.20E-32 | 6.99E-05 | 32.99655418 |
| 71 | rs762679 | A | T | 0.856501 | 0.0310104 | 0.00285024 | 1.40E-27 | 6.16E-05 | 29.09936717 |
| 72 | rs76666449 | C | T | 0.100625 | 0.0295125 | 0.00333186 | 8.20E-19 | 3.01E-05 | 14.20124544 |
| 73 | rs7772289 | T | G | 0.503081 | 0.017549 | 0.00200003 | 1.70E-18 | 8.15E-05 | 38.49628392 |
| 74 | rs7790856 | T | C | 0.289139 | -0.0437199 | 0.00220526 | 1.80E-87 | 0.000342183 | 161.62438 |
| 75 | rs79977579 | A | C | 0.09555 | 0.0281517 | 0.00343182 | 2.30E-16 | 2.46E-05 | 11.63092775 |
| 76 | rs8102497 | A | G | 0.431828 | -0.0149654 | 0.0020233 | 1.40E-13 | 5.69E-05 | 26.8472354 |
| 77 | rs8105767 | G | A | 0.29467 | 0.0328384 | 0.00220117 | 2.50E-50 | 0.000195935 | 92.53336261 |
| 78 | rs869785 | C | T | 0.672471 | -0.0147303 | 0.00212801 | 4.40E-12 | 4.47E-05 | 21.10798344 |
| 79 | rs871134 | T | C | 0.569032 | -0.0182986 | 0.0020263 | 1.70E-19 | 8.47E-05 | 40.00137316 |
| 80 | rs932002 | T | C | 0.150843 | -0.0402052 | 0.00279667 | 7.30E-47 | 0.00011213 | 52.95073805 |
| 81 | rs9398196 | G | A | 0.52005 | -0.0143586 | 0.00201175 | 9.50E-13 | 5.39E-05 | 25.43131503 |
| 82 | rs939916 | A | G | 0.669967 | 0.0241795 | 0.00216724 | 6.60E-29 | 0.000116579 | 55.05163018 |
| 83 | rs9419958 | C | T | 0.86139 | -0.0810098 | 0.00293847 | 2.60E-167 | 0.000384375 | 181.560808 |
| 84 | rs9600019 | T | C | 0.335579 | 0.0127134 | 0.00213096 | 2.40E-09 | 3.36E-05 | 15.87280602 |
| 85 | rs9878436 | T | C | 0.434393 | -0.0143407 | 0.00201819 | 1.20E-12 | 5.25E-05 | 24.81219426 |
| **Exposures/No.** | **SNP** | **A1** | **A2** | **Eaf** | **Beta** | **SE** | **P** | **R^2^** | **F-statistic** |
| **Heavy smoking** |  |  |  |  |  |  |  |  |  |
| 1 | rs11725618 | C | T | 0.287 | 0.036064018 | 0.006157821 | 4.72E-09 | 4.16E-05 | 14.03818695 |
| 2 | rs1435433 | A | G | 0.451 | 0.030476587 | 0.005573535 | 4.55E-08 | 4.39E-05 | 14.80698253 |
| 3 | rs2084533 | T | C | 0.319 | 0.033640552 | 0.005900943 | 1.19E-08 | 4.19E-05 | 14.12104324 |
| 4 | rs215600 | A | G | 0.64 | -0.049250294 | 0.005752503 | 1.11E-17 | 0.000100128 | 33.77981991 |
| 5 | rs2273500 | C | T | 0.159 | 0.068093833 | 0.007795528 | 2.44E-18 | 6.05E-05 | 20.40663062 |
| 6 | rs2386571 | C | A | 0.57 | -0.031868709 | 0.005564518 | 1.02E-08 | 4.77E-05 | 16.07923085 |
| 7 | rs3025383 | C | T | 0.18 | -0.057839414 | 0.00704521 | 2.22E-16 | 5.90E-05 | 19.89753499 |
| 8 | rs4785587 | A | G | 0.511 | -0.033622317 | 0.00553496 | 1.24E-09 | 5.47E-05 | 18.44196638 |
| 9 | rs56113850 | C | T | 0.568 | 0.107204606 | 0.005603675 | 1.39E-81 | 0.000532455 | 179.7098239 |
| 10 | rs58379124 | C | T | 0.748 | 0.06693935 | 0.006501724 | 7.37E-25 | 0.000118462 | 39.96565475 |
| 11 | rs6119248 | G | A | 0.415 | 0.033383557 | 0.005618822 | 2.83E-09 | 5.08E-05 | 17.14068286 |
| 12 | rs632811 | G | A | 0.351 | -0.036713674 | 0.006410485 | 1.02E-08 | 4.43E-05 | 14.94419037 |
| 13 | rs7431710 | A | G | 0.644 | -0.034957881 | 0.005810196 | 1.78E-09 | 4.92E-05 | 16.59943437 |
| 14 | rs790564 | C | A | 0.719 | -0.040892693 | 0.00619302 | 4.03E-11 | 5.22E-05 | 17.61861528 |
| 15 | rs7928017 | A | C | 0.413 | -0.03293136 | 0.005558481 | 3.13E-09 | 5.05E-05 | 17.01941259 |
| 16 | rs7951365 | C | T | 0.306 | 0.038951113 | 0.005967811 | 6.72E-11 | 5.36E-05 | 18.09427701 |
| 17 | rs8034191 | C | T | 0.328 | 0.182566723 | 0.005889249 | 5.39E-211 | 0.001255846 | 424.1697508 |
| 18 | rs806789 | A | C | 0.54 | -0.030854126 | 0.005532648 | 2.45E-08 | 4.58E-05 | 15.45109708 |
| 19 | rs895330 | G | C | 0.206 | -0.039004149 | 0.007016678 | 2.72E-08 | 3.00E-05 | 10.10849911 |
| **Exposures/No.** | **SNP** | **A1** | **A2** | **Eaf** | **Beta** | **SE** | **P** | **R^2^** | **F-statistic** |
| **Smoking initiation** |  |  |  |  |  |  |  |  |  |
| 1 | rs10001365 | A | G | 0.405 | -0.024991752 | 0.00364155 | 6.75E-12 | 1.84E-05 | 22.70022341 |
| 2 | rs1004787 | A | G | 0.581 | 0.02992312 | 0.003571399 | 5.36E-17 | 2.77E-05 | 34.1797197 |
| 3 | rs10159545 | G | C | 0.375 | 0.026250115 | 0.003727266 | 1.89E-12 | 1.89E-05 | 23.25039933 |
| 4 | rs10233018 | G | A | 0.503 | 0.027069038 | 0.003557407 | 2.76E-14 | 2.35E-05 | 28.94958895 |
| 5 | rs10250550 | C | T | 0.597 | -0.020329029 | 0.003610663 | 1.80E-08 | 1.24E-05 | 15.2536369 |
| 6 | rs10279261 | A | G | 0.619 | -0.02141936 | 0.003662633 | 4.97E-09 | 1.31E-05 | 16.13157508 |
| 7 | rs10498846 | T | C | 0.473 | 0.020610294 | 0.003555613 | 6.77E-09 | 1.36E-05 | 16.7512166 |
| 8 | rs1050847 | T | C | 0.505 | -0.021623119 | 0.00358893 | 1.69E-09 | 1.47E-05 | 18.14842605 |
| 9 | rs10905461 | C | T | 0.718 | -0.023955389 | 0.004145052 | 7.50E-09 | 1.10E-05 | 13.52552401 |
| 10 | rs10956809 | C | G | 0.442 | -0.020781065 | 0.003579751 | 6.43E-09 | 1.35E-05 | 16.62346609 |
| 11 | rs11057005 | G | A | 0.43 | -0.02092978 | 0.003578917 | 4.97E-09 | 1.36E-05 | 16.76503965 |
| 12 | rs11078713 | G | A | 0.454 | -0.020172092 | 0.00360561 | 2.21E-08 | 1.26E-05 | 15.51771021 |
| 13 | rs11162019 | T | C | 0.366 | -0.020509083 | 0.003701494 | 3.01E-08 | 1.16E-05 | 14.24764357 |
| 14 | rs1154693 | G | A | 0.856 | 0.032621668 | 0.004912323 | 3.12E-11 | 8.82E-06 | 10.87196076 |
| 15 | rs1160685 | G | C | 0.478 | 0.020772436 | 0.00358893 | 7.13E-09 | 1.36E-05 | 16.71777465 |
| 16 | rs11658881 | G | A | 0.418 | 0.020135721 | 0.003610663 | 2.45E-08 | 1.23E-05 | 15.13193091 |
| 17 | rs11712680 | C | A | 0.174 | -0.027047624 | 0.004578428 | 3.47E-09 | 8.14E-06 | 10.03200144 |
| 18 | rs11721059 | T | C | 0.474 | 0.019935687 | 0.003563355 | 2.21E-08 | 1.27E-05 | 15.60785093 |
| 19 | rs11768481 | A | C | 0.347 | -0.023201667 | 0.003763807 | 7.07E-10 | 1.40E-05 | 17.22113234 |
| 20 | rs11872397 | A | G | 0.252 | -0.024772541 | 0.004094775 | 1.45E-09 | 1.12E-05 | 13.79803742 |
| 21 | rs12042107 | C | T | 0.527 | -0.022283376 | 0.003568196 | 4.24E-10 | 1.58E-05 | 19.44341174 |
| 22 | rs12112638 | G | A | 0.275 | -0.024525968 | 0.004042986 | 1.31E-09 | 1.19E-05 | 14.67415166 |
| 23 | rs12186738 | T | G | 0.154 | -0.033264435 | 0.005020511 | 3.46E-11 | 9.28E-06 | 11.43902116 |
| 24 | rs12333760 | C | T | 0.204 | -0.029046684 | 0.004801269 | 1.45E-09 | 9.65E-06 | 11.88660301 |
| 25 | rs12356821 | C | G | 0.14 | 0.039370036 | 0.005049101 | 6.32E-15 | 1.19E-05 | 14.64078778 |
| 26 | rs12441907 | A | C | 0.186 | -0.029205064 | 0.00452262 | 1.06E-10 | 1.02E-05 | 12.6272046 |
| 27 | rs12474587 | T | G | 0.404 | 0.027632862 | 0.003582345 | 1.22E-14 | 2.33E-05 | 28.65391279 |
| 28 | rs12545053 | G | A | 0.397 | 0.020280798 | 0.003636678 | 2.45E-08 | 1.21E-05 | 14.89027568 |
| 29 | rs12632110 | G | A | 0.647 | -0.023376824 | 0.003752923 | 4.70E-10 | 1.44E-05 | 17.72336699 |
| 30 | rs13030994 | A | G | 0.485 | 0.036092466 | 0.003556296 | 3.35E-24 | 4.18E-05 | 51.45572538 |
| 31 | rs13246563 | G | C | 0.526 | -0.023463104 | 0.003737983 | 3.45E-10 | 1.59E-05 | 19.64701556 |
| 32 | rs134529 | C | T | 0.349 | -0.019983953 | 0.003660777 | 4.79E-08 | 1.10E-05 | 13.54118683 |
| 33 | rs1385108 | T | C | 0.239 | 0.024661699 | 0.004156729 | 2.98E-09 | 1.04E-05 | 12.80439092 |
| 34 | rs1435741 | A | G | 0.425 | 0.029415124 | 0.003590951 | 2.58E-16 | 2.66E-05 | 32.79593631 |
| 35 | rs1445649 | C | T | 0.525 | 0.023993234 | 0.003564837 | 1.69E-11 | 1.83E-05 | 22.59375386 |
| 36 | rs1555445 | T | A | 0.337 | 0.022554797 | 0.003823396 | 3.65E-09 | 1.26E-05 | 15.55097001 |
| 37 | rs1565735 | A | T | 0.212 | -0.037618034 | 0.004461299 | 3.40E-17 | 1.93E-05 | 23.75577865 |
| 38 | rs1899896 | T | C | 0.286 | 0.026448125 | 0.003886909 | 1.01E-11 | 1.53E-05 | 18.90955168 |
| 39 | rs1937443 | G | C | 0.562 | 0.026064233 | 0.003586972 | 3.69E-13 | 2.11E-05 | 25.99458001 |
| 40 | rs2378662 | A | G | 0.556 | 0.020948155 | 0.003566451 | 4.26E-09 | 1.38E-05 | 17.03382146 |
| 41 | rs240963 | C | T | 0.836 | -0.041044352 | 0.004837123 | 2.15E-17 | 1.60E-05 | 19.74326253 |
| 42 | rs2672851 | G | C | 0.529 | -0.030505242 | 0.003737983 | 3.33E-16 | 2.69E-05 | 33.18882474 |
| 43 | rs2892512 | C | T | 0.272 | 0.027730657 | 0.003986002 | 3.48E-12 | 1.56E-05 | 19.16821289 |
| 44 | rs292071 | C | T | 0.244 | 0.023582199 | 0.004009094 | 4.05E-09 | 1.04E-05 | 12.765021 |
| 45 | rs3001723 | A | G | 0.321 | 0.033511798 | 0.003898302 | 8.22E-18 | 2.61E-05 | 32.2151346 |
| 46 | rs301805 | G | T | 0.559 | 0.021467915 | 0.003613287 | 2.83E-09 | 1.41E-05 | 17.40446348 |
| 47 | rs3800227 | G | A | 0.701 | 0.022812119 | 0.004058094 | 1.89E-08 | 1.08E-05 | 13.24677677 |
| 48 | rs3904512 | A | G | 0.429 | -0.021158885 | 0.003576504 | 3.30E-09 | 1.39E-05 | 17.14734403 |
| 49 | rs4044321 | G | A | 0.642 | -0.027841695 | 0.003710577 | 6.22E-14 | 2.10E-05 | 25.88003827 |
| 50 | rs4236259 | G | T | 0.499 | -0.024768919 | 0.00355661 | 3.30E-12 | 1.97E-05 | 24.25033405 |
| 51 | rs4523689 | G | A | 0.408 | -0.020609101 | 0.003643209 | 1.54E-08 | 1.25E-05 | 15.45847088 |
| 52 | rs4543592 | C | T | 0.468 | 0.021931432 | 0.003562439 | 7.45E-10 | 1.53E-05 | 18.87263473 |
| 53 | rs4571506 | T | C | 0.492 | -0.027574664 | 0.003568807 | 1.10E-14 | 2.42E-05 | 29.843033 |
| 54 | rs4674993 | G | A | 0.207 | -0.02521221 | 0.004436185 | 1.32E-08 | 8.61E-06 | 10.60422893 |
| 55 | rs4759228 | C | G | 0.27 | -0.021691308 | 0.003934132 | 3.52E-08 | 9.73E-06 | 11.98377831 |
| 56 | rs4785836 | C | T | 0.398 | -0.020470447 | 0.003658939 | 2.21E-08 | 1.22E-05 | 14.99886702 |
| 57 | rs56820925 | T | C | 0.347 | -0.021863819 | 0.003877149 | 1.71E-08 | 1.17E-05 | 14.41133091 |
| 58 | rs6265 | T | C | 0.203 | -0.031786296 | 0.004578428 | 3.85E-12 | 1.27E-05 | 15.59682562 |
| 59 | rs6433897 | C | T | 0.754 | 0.022448266 | 0.004058094 | 3.17E-08 | 9.21E-06 | 11.35170612 |
| 60 | rs6508144 | G | C | 0.563 | -0.020693519 | 0.003586016 | 7.90E-09 | 1.33E-05 | 16.3858567 |
| 61 | rs6669839 | T | C | 0.204 | 0.026004122 | 0.004395499 | 3.30E-09 | 9.23E-06 | 11.36696574 |
| 62 | rs6728726 | C | T | 0.829 | 0.035448593 | 0.004732794 | 6.89E-14 | 1.29E-05 | 15.9055371 |
| 63 | rs6788098 | T | A | 0.623 | -0.031346052 | 0.003689048 | 1.94E-17 | 2.75E-05 | 33.91624233 |
| 64 | rs6893752 | G | A | 0.766 | -0.024099534 | 0.004073565 | 3.30E-09 | 1.02E-05 | 12.5471858 |
| 65 | rs7197072 | T | C | 0.238 | -0.024767214 | 0.004168596 | 2.83E-09 | 1.04E-05 | 12.80384567 |
| 66 | rs7224742 | T | C | 0.595 | -0.020709882 | 0.003655318 | 1.46E-08 | 1.26E-05 | 15.47076009 |
| 67 | rs7322872 | T | C | 0.782 | -0.025571284 | 0.004334739 | 3.65E-09 | 9.63E-06 | 11.86522244 |
| 68 | rs7555507 | T | C | 0.496 | -0.02414444 | 0.00355604 | 1.12E-11 | 1.87E-05 | 23.04892246 |
| 69 | rs7585579 | G | C | 0.505 | 0.022399646 | 0.0037281 | 1.87E-09 | 1.46E-05 | 18.04843016 |
| 70 | rs7829715 | C | T | 0.521 | -0.026894575 | 0.00355604 | 3.94E-14 | 2.32E-05 | 28.55016927 |
| 71 | rs7921378 | C | G | 0.463 | -0.025460084 | 0.003558155 | 8.34E-13 | 2.07E-05 | 25.46029787 |
| 72 | rs7929518 | G | A | 0.765 | 0.024237689 | 0.004284658 | 1.54E-08 | 9.34E-06 | 11.50569179 |
| 73 | rs7938812 | G | T | 0.424 | 0.043791401 | 0.003636678 | 2.15E-33 | 5.75E-05 | 70.82891301 |
| 74 | rs7969559 | G | A | 0.688 | -0.024375606 | 0.003959459 | 7.45E-10 | 1.32E-05 | 16.27111073 |
| 75 | rs9423279 | G | C | 0.641 | -0.020513169 | 0.003708276 | 3.17E-08 | 1.14E-05 | 14.08342055 |
| 76 | rs9540729 | T | A | 0.501 | -0.019552223 | 0.003557891 | 3.90E-08 | 1.23E-05 | 15.10010234 |
| 77 | rs993700 | C | T | 0.766 | -0.025927991 | 0.004291632 | 1.53E-09 | 1.06E-05 | 13.08492732 |
| **Exposures/No.** | **SNP** | **A1** | **A2** | **Eaf** | **Beta** | **SE** | **P** | **R^2^** | **F-statistic** |
| **Age of smoking initiation ^c^** |  |  |  |  |  |  |  |  |  |
| 1 | rs1038093 | C | T | 0.4 | 0.014897333 | 0.002851197 | 1.74E-07 | 3.84E-05 | 13.10341891 |
| 2 | rs1052486 | G | A | 0.465 | 0.014015034 | 0.002981247 | 2.59E-06 | 3.22E-05 | 10.99543947 |
| 3 | rs10746733 | G | A | 0.468 | -0.012999717 | 0.002765271 | 2.59E-06 | 3.22E-05 | 11.00432318 |
| 4 | rs10753199 | C | T | 0.494 | 0.013000571 | 0.002771731 | 2.73E-06 | 3.22E-05 | 10.99799768 |
| 5 | rs10889582 | G | T | 0.431 | -0.013174753 | 0.002808867 | 2.73E-06 | 3.16E-05 | 10.79011006 |
| 6 | rs10983783 | T | G | 0.551 | 0.01269596 | 0.002770486 | 4.59E-06 | 3.04E-05 | 10.39037795 |
| 7 | rs12449192 | G | C | 0.398 | 0.01366527 | 0.002868173 | 1.89E-06 | 3.19E-05 | 10.87724538 |
| 8 | rs2309885 | T | C | 0.474 | -0.013669812 | 0.002761719 | 7.43E-07 | 3.58E-05 | 12.21636796 |
| 9 | rs2496014 | C | T | 0.665 | 0.013925413 | 0.002872593 | 1.25E-06 | 3.07E-05 | 10.47004525 |
| 10 | rs319748 | A | G | 0.711 | -0.017033929 | 0.003074296 | 3.01E-08 | 3.70E-05 | 12.61586846 |
| 11 | rs4502920 | T | C | 0.357 | -0.014309499 | 0.002839276 | 4.66E-07 | 3.42E-05 | 11.66072201 |
| 12 | rs6781897 | G | A | 0.4 | 0.014741875 | 0.002869134 | 2.78E-07 | 3.71E-05 | 12.67145131 |
| 13 | rs72917329 | A | G | 0.397 | -0.013347227 | 0.002801419 | 1.89E-06 | 3.18E-05 | 10.86794419 |
| 14 | rs7599208 | T | C | 0.558 | -0.019617115 | 0.002779844 | 1.70E-12 | 7.19E-05 | 24.56303906 |
| 15 | rs7817163 | T | G | 0.464 | 0.012744573 | 0.002767944 | 4.14E-06 | 3.09E-05 | 10.54466503 |
| 16 | rs8042134 | G | T | 0.463 | -0.013498916 | 0.002767008 | 1.07E-06 | 3.47E-05 | 11.83435613 |
| **Exposures/No.** | **SNP** | **A1** | **A2** | **Eaf** | **Beta** | **SE** | **P** | **R^2^** | **F-statistic** |
| **Smoking cessation** |  |  |  |  |  |  |  |  |  |
| 1 | rs1009181 | C | T | 0.338 | -0.027571065 | 0.005376203 | 2.92E-07 | 2.15E-05 | 11.76977622 |
| 2 | rs10821537 | G | A | 0.623 | -0.029146883 | 0.005357304 | 5.31E-08 | 2.54E-05 | 13.90466383 |
| 3 | rs113382419 | A | C | 0.0936 | 0.084449289 | 0.008280934 | 2.02E-24 | 3.22E-05 | 17.64702527 |
| 4 | rs11687122 | G | A | 0.494 | 0.023940185 | 0.005163073 | 3.54E-06 | 1.96E-05 | 10.74862417 |
| 5 | rs12484046 | A | G | 0.371 | 0.025354473 | 0.005405592 | 2.73E-06 | 1.88E-05 | 10.2679504 |
| 6 | rs12891477 | T | C | 0.345 | 0.027830929 | 0.005336337 | 1.83E-07 | 2.25E-05 | 12.29327141 |
| 7 | rs13284782 | C | A | 0.334 | 0.026563028 | 0.005550854 | 1.71E-06 | 1.86E-05 | 10.18808925 |
| 8 | rs3001012 | C | T | 0.668 | 0.027180301 | 0.005393087 | 4.66E-07 | 2.06E-05 | 11.2664104 |
| 9 | rs34919890 | T | C | 0.48 | 0.023988115 | 0.005161421 | 3.36E-06 | 1.97E-05 | 10.78289118 |
| 10 | rs4791905 | G | C | 0.545 | -0.024577004 | 0.00519284 | 2.21E-06 | 2.03E-05 | 11.1094644 |
| 11 | rs518425 | G | A | 0.307 | -0.037147419 | 0.00570487 | 7.44E-11 | 3.30E-05 | 18.04181067 |
| 12 | rs56113850 | C | T | 0.568 | -0.055278591 | 0.005200172 | 2.16E-26 | 0.00010134 | 55.46040246 |
| 13 | rs591143 | T | C | 0.583 | -0.026557359 | 0.005311472 | 5.73E-07 | 2.22E-05 | 12.15577468 |
| 14 | rs6011779 | T | C | 0.786 | -0.054699896 | 0.006652933 | 2.00E-16 | 4.16E-05 | 22.74204309 |
| 15 | rs6666704 | G | A | 0.471 | -0.0250109 | 0.005249481 | 1.89E-06 | 2.07E-05 | 11.31201229 |
| 16 | rs7127006 | A | G | 0.278 | 0.032408212 | 0.005792725 | 2.21E-08 | 2.30E-05 | 12.56506365 |
| 17 | rs7968682 | T | G | 0.541 | 0.024155429 | 0.005161699 | 2.87E-06 | 1.99E-05 | 10.87654973 |
| 18 | rs9607805 | T | C | 0.694 | 0.034367998 | 0.005792725 | 2.98E-09 | 2.73E-05 | 14.95077881 |
| 19 | rs986391 | A | G | 0.619 | -0.025763456 | 0.005360412 | 1.54E-06 | 1.99E-05 | 10.89593691 |
| **Exposures/No.** | **SNP** | **A1** | **A2** | **Eaf** | **Beta** | **SE** | **P** | **R^2^** | **F-statistic** |
| **Maternal smoking ^c^** |  |  |  |  |  |  |  |  |  |
| 1 | rs10091451 | T | A | 0.462903 | -0.00504151 | 0.00103015 | 9.90E-07 | 2.99E-05 | 11.90907037 |
| 2 | rs1125112 | A | C | 0.593644 | 0.00536362 | 0.00110922 | 1.30E-06 | 2.84E-05 | 11.28051359 |
| 3 | rs113086489 | T | C | 0.546024 | -0.00487867 | 0.00103602 | 2.50E-06 | 2.76E-05 | 10.99327369 |
| 4 | rs1154780 | G | A | 0.45333 | 0.00536733 | 0.00104148 | 2.60E-07 | 3.31E-05 | 13.16339356 |
| 5 | rs11730046 | G | C | 0.338223 | -0.00536198 | 0.00109159 | 9.00E-07 | 2.72E-05 | 10.80095375 |
| 6 | rs12405972 | T | G | 0.348302 | -0.00806153 | 0.0010749 | 6.40E-14 | 6.42E-05 | 25.53295669 |
| 7 | rs12472263 | G | A | 0.301064 | -0.00558817 | 0.00112031 | 6.10E-07 | 2.63E-05 | 10.4706926 |
| 8 | rs13123903 | T | C | 0.494305 | 0.00520107 | 0.00102369 | 3.80E-07 | 3.24E-05 | 12.90463898 |
| 9 | rs13130893 | G | A | 0.422897 | 0.00554041 | 0.00104098 | 1.00E-07 | 3.48E-05 | 13.82609981 |
| 10 | rs1323341 | G | A | 0.781645 | -0.00682946 | 0.00124268 | 3.90E-08 | 2.59E-05 | 10.30963674 |
| 11 | rs1563245 | G | T | 0.40191 | 0.00513344 | 0.0010506 | 1.00E-06 | 2.89E-05 | 11.47764583 |
| 12 | rs1569216 | G | A | 0.654737 | 0.00549011 | 0.00108678 | 4.40E-07 | 2.90E-05 | 11.53747981 |
| 13 | rs174375 | G | C | 0.681483 | -0.00583204 | 0.00110243 | 1.20E-07 | 3.05E-05 | 12.14901674 |
| 14 | rs1958671 | A | G | 0.519994 | -0.00480842 | 0.00102688 | 2.80E-06 | 2.75E-05 | 10.9452644 |
| 15 | rs2428019 | A | C | 0.239291 | 0.00702278 | 0.0012016 | 5.10E-09 | 3.13E-05 | 12.43533358 |
| 16 | rs254945 | A | G | 0.733334 | -0.00602309 | 0.00117007 | 2.60E-07 | 2.61E-05 | 10.36336941 |
| 17 | rs2693285 | G | A | 0.449737 | 0.00549911 | 0.0010333 | 1.00E-07 | 3.52E-05 | 14.0175912 |
| 18 | rs2724446 | C | T | 0.59959 | -0.00489522 | 0.00105389 | 3.40E-06 | 2.60E-05 | 10.35928205 |
| 19 | rs2804974 | T | C | 0.523357 | -0.0047697 | 0.00103092 | 3.70E-06 | 2.69E-05 | 10.67922176 |
| 20 | rs3115258 | G | T | 0.311542 | -0.00547787 | 0.0011178 | 9.60E-07 | 2.59E-05 | 10.30162768 |
| 21 | rs35566160 | G | A | 0.27477 | 0.00637328 | 0.00116502 | 4.50E-08 | 3.00E-05 | 11.92666714 |
| 22 | rs3746259 | G | A | 0.415088 | -0.004925 | 0.00105478 | 3.00E-06 | 2.66E-05 | 10.58609187 |
| 23 | rs3851536 | A | G | 0.364148 | 0.00497935 | 0.00106831 | 3.10E-06 | 2.53E-05 | 10.06008194 |
| 24 | rs4144892 | T | C | 0.38729 | 0.00518837 | 0.00105175 | 8.10E-07 | 2.90E-05 | 11.54897098 |
| 25 | rs4865667 | T | C | 0.387775 | -0.00581087 | 0.00105303 | 3.40E-08 | 3.64E-05 | 14.45785022 |
| 26 | rs55741516 | C | T | 0.636786 | -0.00548761 | 0.00106986 | 2.90E-07 | 3.06E-05 | 12.16979286 |
| 27 | rs56322375 | T | G | 0.239664 | 0.00655095 | 0.00122725 | 9.40E-08 | 2.61E-05 | 10.38406308 |
| 28 | rs575030 | C | T | 0.336841 | 0.00523469 | 0.00108335 | 1.40E-06 | 2.62E-05 | 10.43045847 |
| 29 | rs576982 | T | C | 0.227864 | -0.00933094 | 0.00122232 | 2.30E-14 | 5.16E-05 | 20.50481694 |
| 30 | rs6011779 | T | C | 0.808714 | -0.00993868 | 0.00130401 | 2.50E-14 | 4.52E-05 | 17.97139979 |
| 31 | rs6064904 | G | T | 0.627805 | -0.00495518 | 0.0010647 | 3.30E-06 | 2.55E-05 | 10.12223523 |
| 32 | rs62477310 | C | T | 0.486743 | -0.00577877 | 0.0010297 | 2.00E-08 | 3.96E-05 | 15.7360098 |
| 33 | rs6730303 | C | G | 0.56353 | 0.00492736 | 0.00103406 | 1.90E-06 | 2.81E-05 | 11.16925392 |
| 34 | rs6744328 | G | A | 0.538588 | 0.00493907 | 0.00103043 | 1.60E-06 | 2.87E-05 | 11.41863721 |
| 35 | rs7002049 | C | T | 0.7847 | 0.0075623 | 0.00124967 | 1.40E-09 | 3.11E-05 | 12.37311641 |
| 36 | rs7386735 | C | T | 0.676952 | 0.00554666 | 0.00110692 | 5.40E-07 | 2.76E-05 | 10.98174548 |
| 37 | rs7511894 | C | T | 0.557746 | -0.00493154 | 0.00102888 | 1.60E-06 | 2.85E-05 | 11.3333771 |
| 38 | rs75596189 | T | C | 0.109986 | 0.0120528 | 0.00164225 | 2.10E-13 | 2.65E-05 | 10.545032 |
| 39 | rs7909492 | A | G | 0.364563 | -0.00495804 | 0.00106452 | 3.20E-06 | 2.53E-05 | 10.05019128 |
| 40 | rs876793 | C | T | 0.35213 | -0.00537022 | 0.00107559 | 6.00E-07 | 2.86E-05 | 11.37357334 |
| **Exposures/No.** | **SNP** | **A1** | **A2** | **Eaf** | **Beta** | **SE** | **P** | **R^2^** | **F-statistic** |
| **Heavy drinking** |  |  |  |  |  |  |  |  |  |
| 1 | rs10085696 | G | A | 0.201 | -0.016051676 | 0.002494711 | 1.24E-10 | 1.41E-05 | 13.29775512 |
| 2 | rs10753661 | A | G | 0.702 | -0.011365553 | 0.002075057 | 4.32E-08 | 1.33E-05 | 12.55189569 |
| 3 | rs1123285 | G | C | 0.339 | -0.012418317 | 0.002049886 | 1.38E-09 | 1.75E-05 | 16.44765107 |
| 4 | rs11860773 | C | T | 0.176 | -0.015005068 | 0.002443809 | 8.25E-10 | 1.16E-05 | 10.93491505 |
| 5 | rs1229984 | C | T | 0.953 | 0.188115393 | 0.00617852 | 1.33E-203 | 8.82E-05 | 83.04966838 |
| 6 | rs1260326 | C | T | 0.595 | 0.02381201 | 0.001984334 | 3.55E-33 | 7.37E-05 | 69.4057815 |
| 7 | rs13107325 | T | C | 0.0654 | -0.036454753 | 0.00391286 | 1.20E-20 | 1.13E-05 | 10.61102151 |
| 8 | rs13332432 | G | C | 0.296 | 0.014004968 | 0.002140722 | 6.06E-11 | 1.90E-05 | 17.83796758 |
| 9 | rs1387766 | A | G | 0.622 | -0.01082702 | 0.001983357 | 4.79E-08 | 1.49E-05 | 14.01308687 |
| 10 | rs153106 | C | T | 0.409 | -0.013611756 | 0.001958577 | 3.66E-12 | 2.48E-05 | 23.35057858 |
| 11 | rs17542254 | G | A | 0.251 | 0.013141799 | 0.002146047 | 9.14E-10 | 1.50E-05 | 14.1001035 |
| 12 | rs2299409 | A | G | 0.493 | -0.010554626 | 0.001933458 | 4.79E-08 | 1.58E-05 | 14.89728196 |
| 13 | rs28601761 | G | C | 0.405 | 0.011298868 | 0.001955067 | 7.50E-09 | 1.71E-05 | 16.09737526 |
| 14 | rs28680958 | A | G | 0.23 | -0.01358482 | 0.002368405 | 9.70E-09 | 1.24E-05 | 11.65329791 |
| 15 | rs28712821 | A | G | 0.594 | 0.028333875 | 0.001974115 | 1.02E-46 | 0.000105558 | 99.36980725 |
| 16 | rs28732378 | G | A | 0.729 | -0.016727407 | 0.002190758 | 2.25E-14 | 2.45E-05 | 23.03588787 |
| 17 | rs331939 | A | G | 0.339 | -0.011901612 | 0.002029208 | 4.49E-09 | 1.64E-05 | 15.416855 |
| 18 | rs34121753 | G | A | 0.532 | 0.011069397 | 0.001950725 | 1.39E-08 | 1.70E-05 | 16.03429183 |
| 19 | rs34704785 | T | C | 0.412 | -0.010581507 | 0.001935138 | 4.55E-08 | 1.54E-05 | 14.4871002 |
| 20 | rs4233567 | T | C | 0.34 | -0.012589937 | 0.002010854 | 3.83E-10 | 1.87E-05 | 17.59325015 |
| 21 | rs4752999 | T | C | 0.321 | -0.01456432 | 0.002070082 | 1.98E-12 | 2.29E-05 | 21.57839617 |
| 22 | rs4916723 | C | A | 0.404 | -0.011270904 | 0.001953155 | 7.90E-09 | 1.70E-05 | 16.03644794 |
| 23 | rs528301 | A | G | 0.605 | 0.015591937 | 0.001948992 | 1.24E-15 | 3.25E-05 | 30.58973301 |
| 24 | rs55872084 | T | G | 0.218 | 0.012731384 | 0.002268404 | 1.99E-08 | 1.14E-05 | 10.74008456 |
| 25 | rs55932213 | G | A | 0.701 | 0.012476712 | 0.002216004 | 1.80E-08 | 1.41E-05 | 13.2887347 |
| 26 | rs6106989 | A | G | 0.628 | 0.010899442 | 0.001983357 | 3.90E-08 | 1.50E-05 | 14.11058144 |
| 27 | rs6739804 | C | T | 0.66 | -0.01296884 | 0.002082022 | 4.70E-10 | 1.85E-05 | 17.41372156 |
| 28 | rs78234152 | A | G | 0.0986 | 0.027653801 | 0.00307075 | 2.15E-19 | 1.53E-05 | 14.41620464 |
| 29 | rs838145 | A | G | 0.584 | -0.015797822 | 0.001940173 | 3.87E-16 | 3.42E-05 | 32.21540781 |
| **Exposures/No.** | **SNP** | **A1** | **A2** | **Eaf** | **Beta** | **SE** | **P** | **R^2^** | **F-statistic** |
| **Body mass index ^d^** |  |  |  |  |  |  |  |  |  |
| 1 | rs10009336 | T | C | 0.1638 | -0.014 | 0.0022 | 2.20E-10 | 1.40E-05 | 11.09352931 |
| 2 | rs1006896 | C | A | 0.1061 | -0.0234 | 0.0027 | 5.50E-18 | 2.06E-05 | 14.24774688 |
| 3 | rs10132280 | A | C | 0.3017 | -0.0223 | 0.0018 | 5.60E-35 | 8.22E-05 | 64.67652357 |
| 4 | rs10169594 | C | T | 0.3596 | 0.0121 | 0.0018 | 2.00E-11 | 3.04E-05 | 20.81318994 |
| 5 | rs10182181 | G | A | 0.4753 | 0.0325 | 0.0016 | 6.70E-90 | 0.000259806 | 205.8483458 |
| 6 | rs10192119 | G | T | 0.1673 | 0.0166 | 0.0022 | 3.00E-14 | 1.99E-05 | 15.86327618 |
| 7 | rs10197031 | C | T | 0.2834 | 0.0166 | 0.0019 | 1.90E-18 | 4.48E-05 | 31.00514997 |
| 8 | rs10243319 | C | T | 0.3939 | -0.0107 | 0.0018 | 1.20E-09 | 2.44E-05 | 16.87299408 |
| 9 | rs10248136 | T | C | 0.5142 | -0.0097 | 0.0017 | 2.00E-08 | 2.37E-05 | 16.2657549 |
| 10 | rs10269783 | A | G | 0.3896 | 0.0133 | 0.0017 | 1.40E-15 | 3.68E-05 | 29.1127883 |
| 11 | rs10408324 | T | C | 0.2744 | -0.0124 | 0.0019 | 9.50E-11 | 2.46E-05 | 16.96121471 |
| 12 | rs10478110 | C | A | 0.4348 | 0.01 | 0.0017 | 9.60E-09 | 2.50E-05 | 17.00722353 |
| 13 | rs1048932 | A | C | 0.4162 | -0.016 | 0.0017 | 3.80E-22 | 5.41E-05 | 43.04876569 |
| 14 | rs10492229 | T | C | 0.2268 | 0.0142 | 0.0019 | 7.70E-14 | 2.46E-05 | 19.59043093 |
| 15 | rs10510419 | T | G | 0.1416 | -0.0177 | 0.0023 | 2.20E-14 | 1.82E-05 | 14.39728626 |
| 16 | rs1064213 | A | G | 0.492 | 0.012 | 0.0017 | 2.40E-12 | 3.60E-05 | 24.90794079 |
| 17 | rs10733051 | G | A | 0.4802 | -0.0097 | 0.0016 | 2.90E-09 | 2.35E-05 | 18.34851874 |
| 18 | rs10742752 | C | T | 0.6159 | 0.0124 | 0.0017 | 1.10E-13 | 3.18E-05 | 25.17344947 |
| 19 | rs10747488 | A | C | 0.7601 | -0.0123 | 0.002 | 1.20E-09 | 2.00E-05 | 13.79394972 |
| 20 | rs10750215 | T | G | 0.3883 | 0.0108 | 0.0017 | 1.30E-10 | 2.43E-05 | 19.17321706 |
| 21 | rs1075901 | C | T | 0.5639 | 0.0121 | 0.0016 | 1.20E-13 | 3.54E-05 | 28.12957884 |
| 22 | rs10768994 | C | T | 0.4337 | -0.0114 | 0.0017 | 6.40E-12 | 2.79E-05 | 22.08965127 |
| 23 | rs10795422 | G | A | 0.6905 | 0.0139 | 0.0019 | 9.30E-14 | 3.31E-05 | 22.87651318 |
| 24 | rs10811871 | G | A | 0.3829 | -0.0108 | 0.0018 | 1.60E-09 | 2.48E-05 | 17.0130786 |
| 25 | rs10832778 | G | C | 0.6222 | 0.0125 | 0.0017 | 1.30E-13 | 3.25E-05 | 25.41892196 |
| 26 | rs10867256 | T | C | 0.553 | -0.0118 | 0.0017 | 8.70E-12 | 3.45E-05 | 23.82004434 |
| 27 | rs10887578 | C | G | 0.4896 | 0.0128 | 0.0017 | 1.60E-13 | 4.17E-05 | 28.33485578 |
| 28 | rs10914462 | G | A | 0.4255 | -0.0112 | 0.0017 | 1.50E-10 | 3.08E-05 | 21.22119797 |
| 29 | rs10915840 | A | G | 0.283 | -0.0118 | 0.0019 | 1.30E-09 | 2.29E-05 | 15.65312515 |
| 30 | rs10920678 | G | A | 0.5709 | -0.0155 | 0.0016 | 1.50E-21 | 5.83E-05 | 45.98288385 |
| 31 | rs10938397 | G | A | 0.4317 | 0.0324 | 0.0016 | 3.40E-86 | 0.000253561 | 201.2559807 |
| 32 | rs10942267 | G | A | 0.3088 | -0.0156 | 0.0019 | 3.90E-17 | 4.18E-05 | 28.77861495 |
| 33 | rs10953740 | G | A | 0.5534 | -0.0153 | 0.0017 | 1.00E-18 | 5.85E-05 | 40.04027261 |
| 34 | rs10962550 | C | G | 0.1801 | 0.0182 | 0.0022 | 6.20E-16 | 2.93E-05 | 20.21219421 |
| 35 | rs10968114 | C | A | 0.4681 | -0.0113 | 0.0017 | 6.10E-11 | 3.21E-05 | 22.00241409 |
| 36 | rs10971709 | T | C | 0.2062 | 0.0132 | 0.0021 | 6.20E-10 | 1.88E-05 | 12.93438915 |
| 37 | rs11030618 | T | C | 0.5679 | 0.011 | 0.0017 | 2.40E-10 | 2.98E-05 | 20.54874642 |
| 38 | rs11084553 | G | A | 0.1518 | -0.021 | 0.0024 | 1.80E-18 | 2.85E-05 | 19.71638429 |
| 39 | rs11105839 | A | T | 0.3799 | -0.0109 | 0.0017 | 1.10E-10 | 2.48E-05 | 19.36983073 |
| 40 | rs11115176 | C | T | 0.2399 | -0.0121 | 0.0019 | 2.00E-10 | 1.87E-05 | 14.79113584 |
| 41 | rs11118308 | G | A | 0.4703 | -0.0101 | 0.0016 | 4.80E-10 | 2.50E-05 | 19.85397576 |
| 42 | rs11150911 | C | A | 0.7191 | -0.0133 | 0.0018 | 4.70E-13 | 2.82E-05 | 22.05669501 |
| 43 | rs11170468 | C | A | 0.2326 | -0.0123 | 0.0019 | 1.90E-10 | 1.88E-05 | 14.96137815 |
| 44 | rs11173522 | A | C | 0.2078 | 0.0128 | 0.0021 | 1.10E-09 | 1.77E-05 | 12.23201914 |
| 45 | rs11185111 | A | G | 0.3042 | -0.0129 | 0.0019 | 7.70E-12 | 2.84E-05 | 19.51447753 |
| 46 | rs11251352 | G | A | 0.5988 | 0.0109 | 0.0018 | 7.00E-10 | 2.55E-05 | 17.61937573 |
| 47 | rs1144387 | C | G | 0.5714 | 0.0098 | 0.0017 | 1.60E-08 | 2.37E-05 | 16.27742583 |
| 48 | rs11496125 | T | C | 0.4212 | 0.0169 | 0.0017 | 3.00E-22 | 7.04E-05 | 48.18942549 |
| 49 | rs11538 | G | A | 0.1805 | 0.0135 | 0.0023 | 3.30E-09 | 1.47E-05 | 10.19232266 |
| 50 | rs1158805 | A | C | 0.3766 | -0.0137 | 0.0018 | 1.20E-14 | 3.93E-05 | 27.20126205 |
| 51 | rs11609659 | C | T | 0.2371 | -0.0154 | 0.002 | 2.20E-14 | 3.16E-05 | 21.44979035 |
| 52 | rs11611246 | T | G | 0.21 | 0.024 | 0.002 | 5.00E-32 | 6.13E-05 | 47.78200503 |
| 53 | rs11615578 | T | C | 0.2474 | 0.013 | 0.002 | 8.10E-11 | 2.35E-05 | 15.73365156 |
| 54 | rs11656076 | A | G | 0.2254 | -0.0142 | 0.0021 | 5.60E-12 | 2.31E-05 | 15.96644663 |
| 55 | rs11672660 | T | C | 0.2049 | -0.034 | 0.0021 | 1.70E-60 | 0.00011115 | 85.42010412 |
| 56 | rs11713193 | A | G | 0.5073 | 0.0239 | 0.0017 | 2.40E-44 | 0.000142748 | 98.81801448 |
| 57 | rs11736228 | T | A | 0.2587 | -0.0139 | 0.002 | 4.10E-12 | 2.68E-05 | 18.52679994 |
| 58 | rs11738695 | A | C | 0.586 | 0.0097 | 0.0017 | 2.00E-08 | 2.28E-05 | 15.79727743 |
| 59 | rs11739877 | T | C | 0.6118 | 0.0117 | 0.0018 | 6.60E-11 | 2.90E-05 | 20.06933784 |
| 60 | rs11781699 | C | T | 0.1896 | 0.0132 | 0.0021 | 3.10E-10 | 1.55E-05 | 12.14178805 |
| 61 | rs11855853 | T | C | 0.2649 | -0.0145 | 0.002 | 2.40E-13 | 3.00E-05 | 20.47133392 |
| 62 | rs1187352 | C | T | 0.6518 | 0.0119 | 0.0018 | 6.00E-11 | 2.88E-05 | 19.83961698 |
| 63 | rs11880870 | G | A | 0.4801 | -0.0189 | 0.0017 | 1.00E-28 | 8.60E-05 | 61.70827858 |
| 64 | rs11889536 | G | A | 0.1493 | -0.0189 | 0.0024 | 6.40E-15 | 2.29E-05 | 15.75346275 |
| 65 | rs11908637 | A | G | 0.236 | -0.012 | 0.0021 | 4.90E-09 | 1.70E-05 | 11.77512157 |
| 66 | rs11945861 | A | G | 0.2369 | -0.0148 | 0.002 | 5.00E-13 | 2.90E-05 | 19.79936566 |
| 67 | rs11951673 | T | C | 0.3941 | -0.0123 | 0.0017 | 1.10E-13 | 3.16E-05 | 25.00128733 |
| 68 | rs12041258 | C | T | 0.2287 | -0.0146 | 0.002 | 9.50E-13 | 2.73E-05 | 18.80077742 |
| 69 | rs12044597 | G | A | 0.5029 | 0.0143 | 0.0016 | 1.70E-18 | 5.06E-05 | 39.94002973 |
| 70 | rs12049202 | T | C | 0.203 | 0.024 | 0.0022 | 1.00E-28 | 5.57E-05 | 38.5109653 |
| 71 | rs12098284 | T | C | 0.1241 | 0.0178 | 0.0026 | 1.80E-11 | 1.48E-05 | 10.18954509 |
| 72 | rs1218822 | A | G | 0.6663 | 0.0168 | 0.0017 | 1.90E-22 | 5.46E-05 | 43.43095478 |
| 73 | rs12299814 | A | C | 0.2525 | -0.0157 | 0.002 | 5.20E-15 | 3.38E-05 | 23.26244241 |
| 74 | rs12328930 | C | T | 0.4235 | 0.0098 | 0.0017 | 1.80E-08 | 2.35E-05 | 16.22728929 |
| 75 | rs12334877 | A | G | 0.198 | -0.0144 | 0.0022 | 7.70E-11 | 1.99E-05 | 13.60681713 |
| 76 | rs12364470 | G | T | 0.1626 | 0.0178 | 0.0022 | 1.10E-15 | 2.26E-05 | 17.82735308 |
| 77 | rs12369179 | T | C | 0.08782 | -0.0359 | 0.0031 | 2.50E-31 | 3.19E-05 | 21.48730931 |
| 78 | rs12416812 | A | G | 0.5088 | 0.0111 | 0.0016 | 6.10E-12 | 3.03E-05 | 24.05766779 |
| 79 | rs12422552 | C | G | 0.2663 | -0.0134 | 0.002 | 1.60E-11 | 2.54E-05 | 17.54199873 |
| 80 | rs12429545 | A | G | 0.1248 | 0.0316 | 0.0025 | 9.60E-38 | 4.48E-05 | 34.90313066 |
| 81 | rs12448257 | A | G | 0.218 | 0.0184 | 0.002 | 8.10E-20 | 3.70E-05 | 28.85917148 |
| 82 | rs12546578 | A | T | 0.7246 | 0.0146 | 0.002 | 1.00E-13 | 3.09E-05 | 21.2691495 |
| 83 | rs12564992 | G | A | 0.1144 | 0.0196 | 0.0026 | 5.30E-14 | 1.45E-05 | 11.51500874 |
| 84 | rs12593036 | G | A | 0.2993 | -0.0154 | 0.0019 | 3.80E-16 | 4.02E-05 | 27.55619487 |
| 85 | rs12602912 | T | C | 0.2048 | 0.0176 | 0.0021 | 9.90E-18 | 2.94E-05 | 22.87887828 |
| 86 | rs1260326 | C | T | 0.5973 | 0.0105 | 0.0017 | 3.90E-10 | 2.34E-05 | 18.35244572 |
| 87 | rs12629015 | G | A | 0.1852 | -0.0135 | 0.0023 | 2.10E-09 | 1.50E-05 | 10.39774448 |
| 88 | rs1266874 | G | A | 0.3558 | 0.014 | 0.0018 | 9.80E-15 | 4.01E-05 | 27.7321725 |
| 89 | rs1268065 | A | G | 0.4794 | -0.0102 | 0.0017 | 1.00E-09 | 2.37E-05 | 17.96982386 |
| 90 | rs12718572 | T | C | 0.4024 | -0.0117 | 0.0018 | 3.00E-11 | 2.96E-05 | 20.32061554 |
| 91 | rs12779328 | T | C | 0.2833 | 0.0105 | 0.0019 | 4.50E-08 | 1.80E-05 | 12.40200525 |
| 92 | rs1285997 | G | C | 0.7153 | 0.0142 | 0.0019 | 1.20E-13 | 3.32E-05 | 22.75036376 |
| 93 | rs12888545 | G | A | 0.2519 | 0.0136 | 0.002 | 9.10E-12 | 2.53E-05 | 17.4279126 |
| 94 | rs12888955 | A | G | 0.6513 | -0.0178 | 0.0018 | 1.40E-22 | 6.42E-05 | 44.42062282 |
| 95 | rs12922346 | C | G | 0.2657 | 0.0136 | 0.002 | 1.00E-11 | 2.65E-05 | 18.04359855 |
| 96 | rs12936083 | G | A | 0.3267 | 0.0139 | 0.0019 | 4.10E-13 | 3.72E-05 | 23.54642136 |
| 97 | rs12939549 | G | A | 0.4335 | -0.018 | 0.0016 | 2.70E-28 | 7.83E-05 | 62.16657868 |
| 98 | rs1296328 | C | A | 0.5657 | -0.0179 | 0.0018 | 4.90E-24 | 7.11E-05 | 48.5955679 |
| 99 | rs12981256 | A | G | 0.5325 | 0.0142 | 0.0018 | 1.10E-15 | 4.57E-05 | 30.98713755 |
| 100 | rs13047416 | G | C | 0.3769 | -0.0154 | 0.0018 | 2.20E-17 | 5.03E-05 | 34.38198124 |
| 101 | rs13110266 | A | G | 0.4065 | -0.0117 | 0.0017 | 1.90E-12 | 2.89E-05 | 22.85580905 |
| 102 | rs13132853 | G | A | 0.3452 | -0.0142 | 0.0018 | 4.70E-15 | 4.12E-05 | 28.13570239 |
| 103 | rs13147390 | C | T | 0.3569 | 0.0103 | 0.0018 | 1.00E-08 | 2.21E-05 | 15.03117054 |
| 104 | rs13174863 | G | A | 0.1548 | 0.0192 | 0.0023 | 2.90E-16 | 2.36E-05 | 18.23544384 |
| 105 | rs1320903 | A | G | 0.3174 | 0.0216 | 0.0018 | 9.20E-32 | 9.02E-05 | 62.40273539 |
| 106 | rs1321432 | C | A | 0.6321 | 0.0201 | 0.0018 | 3.50E-29 | 8.45E-05 | 58.00001485 |
| 107 | rs13263601 | C | A | 0.3478 | 0.0154 | 0.0018 | 2.20E-17 | 4.84E-05 | 33.20905757 |
| 108 | rs1327259 | G | A | 0.3872 | -0.0155 | 0.0018 | 1.70E-18 | 5.13E-05 | 35.1903431 |
| 109 | rs13287131 | C | T | 0.2491 | 0.0123 | 0.002 | 6.80E-10 | 2.07E-05 | 14.14958738 |
| 110 | rs1330052 | G | C | 0.3504 | 0.0132 | 0.0018 | 1.50E-13 | 3.54E-05 | 24.48257419 |
| 111 | rs13329567 | T | C | 0.2308 | -0.0293 | 0.002 | 1.00E-50 | 9.60E-05 | 76.21157153 |
| 112 | rs1365466 | T | C | 0.7406 | -0.0137 | 0.0019 | 3.30E-13 | 2.52E-05 | 19.97687107 |
| 113 | rs1371108 | A | C | 0.3247 | 0.0119 | 0.0018 | 9.00E-11 | 2.80E-05 | 19.16764851 |
| 114 | rs138289 | T | A | 0.4829 | -0.0103 | 0.0017 | 3.30E-09 | 2.67E-05 | 18.33363864 |
| 115 | rs1412235 | C | G | 0.3175 | 0.0246 | 0.0017 | 6.00E-45 | 0.000114853 | 90.76063712 |
| 116 | rs1421334 | C | A | 0.5431 | -0.0125 | 0.0018 | 1.00E-12 | 3.52E-05 | 23.93425794 |
| 117 | rs1430387 | C | T | 0.4295 | -0.0114 | 0.0017 | 5.80E-11 | 3.20E-05 | 22.03805674 |
| 118 | rs1431659 | G | A | 0.7344 | -0.0196 | 0.0019 | 6.00E-24 | 6.02E-05 | 41.51648303 |
| 119 | rs1436344 | C | G | 0.5922 | 0.0141 | 0.0017 | 4.10E-16 | 4.80E-05 | 33.228107 |
| 120 | rs1452075 | T | C | 0.7277 | 0.0141 | 0.0018 | 1.30E-14 | 3.10E-05 | 24.31843341 |
| 121 | rs1465900 | C | A | 0.2188 | -0.0125 | 0.002 | 4.80E-10 | 1.71E-05 | 13.35383194 |
| 122 | rs1472169 | T | C | 0.3772 | -0.0139 | 0.0018 | 2.80E-15 | 4.06E-05 | 28.01891099 |
| 123 | rs1476322 | A | G | 0.569 | 0.0101 | 0.0017 | 5.00E-09 | 2.50E-05 | 17.31306821 |
| 124 | rs1477199 | G | A | 0.1451 | 0.0228 | 0.0024 | 9.40E-22 | 2.82E-05 | 22.39087588 |
| 125 | rs1492767 | T | C | 0.4957 | 0.0094 | 0.0016 | 1.00E-08 | 2.17E-05 | 17.25686763 |
| 126 | rs1503526 | C | T | 0.4838 | 0.014 | 0.0017 | 5.50E-17 | 4.53E-05 | 33.87588202 |
| 127 | rs1521527 | C | G | 0.5324 | -0.0121 | 0.0017 | 3.10E-12 | 3.72E-05 | 25.22495009 |
| 128 | rs1522569 | G | T | 0.1819 | -0.0164 | 0.0022 | 2.90E-13 | 2.40E-05 | 16.53943154 |
| 129 | rs1528435 | T | C | 0.6331 | 0.0164 | 0.0017 | 9.10E-23 | 5.44E-05 | 43.23768407 |
| 130 | rs1538247 | C | T | 0.3181 | 0.0108 | 0.0019 | 1.00E-08 | 2.05E-05 | 14.01723352 |
| 131 | rs1552893 | G | A | 0.2777 | -0.0126 | 0.0019 | 8.10E-11 | 2.55E-05 | 17.64278759 |
| 132 | rs156201 | C | G | 0.7606 | 0.0123 | 0.002 | 5.80E-10 | 1.99E-05 | 13.77425395 |
| 133 | rs1624134 | C | G | 0.4068 | 0.0101 | 0.0018 | 1.10E-08 | 2.20E-05 | 15.19560928 |
| 134 | rs1656377 | C | T | 0.5885 | 0.0099 | 0.0017 | 1.60E-08 | 2.37E-05 | 16.4258519 |
| 135 | rs1681740 | C | A | 0.3933 | -0.0115 | 0.0018 | 1.10E-10 | 2.89E-05 | 19.48004159 |
| 136 | rs16849710 | G | A | 0.515 | -0.0116 | 0.0018 | 6.00E-11 | 3.11E-05 | 20.74732701 |
| 137 | rs16871902 | A | G | 0.4877 | 0.0125 | 0.0017 | 4.60E-13 | 3.91E-05 | 27.01749113 |
| 138 | rs16903285 | C | T | 0.1407 | 0.0331 | 0.0026 | 7.60E-38 | 5.70E-05 | 39.19238342 |
| 139 | rs17001561 | A | G | 0.1573 | 0.0151 | 0.0023 | 3.80E-11 | 1.44E-05 | 11.42707529 |
| 140 | rs17014375 | G | T | 0.1348 | 0.0172 | 0.0025 | 1.10E-11 | 1.60E-05 | 11.04126818 |
| 141 | rs17033117 | T | C | 0.1872 | 0.0137 | 0.0022 | 8.90E-10 | 1.71E-05 | 11.801072 |
| 142 | rs17056301 | C | T | 0.2636 | 0.0118 | 0.002 | 2.40E-09 | 1.96E-05 | 13.51451523 |
| 143 | rs17113297 | T | C | 0.2082 | 0.0166 | 0.0021 | 2.10E-15 | 3.00E-05 | 20.60229376 |
| 144 | rs17203016 | G | A | 0.196 | 0.015 | 0.002 | 2.10E-13 | 2.25E-05 | 17.72855463 |
| 145 | rs17207196 | T | C | 0.4118 | -0.0221 | 0.0018 | 2.10E-35 | 0.000109175 | 73.03432394 |
| 146 | rs17238110 | G | A | 0.1634 | -0.0353 | 0.005 | 2.00E-12 | 1.76E-05 | 13.62748842 |
| 147 | rs17311369 | T | C | 0.3278 | -0.0104 | 0.0019 | 3.10E-08 | 1.96E-05 | 13.20395874 |
| 148 | rs17399237 | C | T | 0.5497 | -0.0129 | 0.0017 | 6.70E-14 | 4.13E-05 | 28.50728898 |
| 149 | rs17424296 | A | G | 0.3659 | -0.0108 | 0.0018 | 2.40E-09 | 2.44E-05 | 16.70559664 |
| 150 | rs17513613 | C | T | 0.3236 | 0.0186 | 0.0018 | 3.60E-26 | 5.92E-05 | 46.74633824 |
| 151 | rs175165 | G | T | 0.3941 | -0.0103 | 0.0018 | 5.20E-09 | 2.26E-05 | 15.63779081 |
| 152 | rs17551974 | A | C | 0.1782 | -0.0141 | 0.0022 | 1.90E-10 | 1.74E-05 | 12.03103525 |
| 153 | rs17636031 | C | T | 0.2701 | 0.016 | 0.0019 | 1.20E-17 | 3.57E-05 | 27.96179903 |
| 154 | rs17710386 | C | T | 0.3319 | 0.0126 | 0.0018 | 1.00E-12 | 2.77E-05 | 21.73130126 |
| 155 | rs17789218 | C | T | 0.2392 | 0.013 | 0.0019 | 7.40E-12 | 2.15E-05 | 17.039208 |
| 156 | rs17806379 | T | C | 0.1789 | -0.0258 | 0.0022 | 1.50E-30 | 5.86E-05 | 40.40681416 |
| 157 | rs1784460 | A | T | 0.4035 | 0.0132 | 0.0018 | 9.00E-14 | 3.81E-05 | 25.88821402 |
| 158 | rs1804528 | A | G | 0.3507 | 0.0109 | 0.002 | 3.00E-08 | 2.61E-05 | 13.52738397 |
| 159 | rs1830074 | C | T | 0.288 | 0.0115 | 0.0019 | 1.40E-09 | 2.18E-05 | 15.02446978 |
| 160 | rs1836303 | G | A | 0.3873 | 0.0116 | 0.0018 | 5.30E-11 | 2.86E-05 | 19.71094767 |
| 161 | rs1843328 | A | C | 0.5085 | -0.0099 | 0.0017 | 7.90E-09 | 2.47E-05 | 16.95221595 |
| 162 | rs1863652 | A | G | 0.3449 | -0.0115 | 0.0018 | 1.40E-10 | 2.66E-05 | 18.44555695 |
| 163 | rs1885728 | A | G | 0.6787 | 0.0108 | 0.0019 | 1.00E-08 | 2.07E-05 | 14.09180341 |
| 164 | rs1891216 | G | T | 0.3759 | 0.0107 | 0.0018 | 2.40E-09 | 2.42E-05 | 16.58014376 |
| 165 | rs1896767 | A | G | 0.5376 | -0.0109 | 0.0017 | 2.40E-10 | 2.98E-05 | 20.43967111 |
| 166 | rs189843 | C | G | 0.5557 | -0.0098 | 0.0017 | 1.70E-08 | 2.39E-05 | 16.41005914 |
| 167 | rs1927790 | C | T | 0.4109 | 0.0148 | 0.0016 | 1.80E-19 | 5.21E-05 | 41.42477707 |
| 168 | rs1937683 | T | C | 0.6699 | 0.0109 | 0.0018 | 3.20E-09 | 2.34E-05 | 16.21819059 |
| 169 | rs1948080 | G | T | 0.3749 | -0.0137 | 0.0018 | 1.10E-14 | 3.93E-05 | 27.15231576 |
| 170 | rs1982441 | T | G | 0.1381 | 0.0175 | 0.0026 | 7.00E-12 | 1.57E-05 | 10.78488464 |
| 171 | rs1982725 | T | C | 0.4778 | 0.0097 | 0.0017 | 3.30E-08 | 2.38E-05 | 16.24679465 |
| 172 | rs2007231 | T | C | 0.6387 | -0.0104 | 0.0018 | 5.20E-09 | 2.23E-05 | 15.40724386 |
| 173 | rs200810 | C | T | 0.3716 | -0.0136 | 0.0017 | 5.50E-16 | 3.77E-05 | 29.89077065 |
| 174 | rs2009416 | T | C | 0.361 | -0.0121 | 0.0018 | 1.10E-11 | 3.01E-05 | 20.84853868 |
| 175 | rs2033529 | G | A | 0.2936 | 0.0205 | 0.0018 | 1.90E-30 | 6.79E-05 | 53.80564627 |
| 176 | rs2051559 | C | T | 0.1308 | 0.0176 | 0.0026 | 5.00E-12 | 1.51E-05 | 10.41936859 |
| 177 | rs2065418 | G | T | 0.3623 | -0.0166 | 0.0018 | 3.60E-20 | 5.68E-05 | 39.30151863 |
| 178 | rs208015 | C | T | 0.9216 | -0.0356 | 0.0034 | 1.40E-25 | 2.29E-05 | 15.8430714 |
| 179 | rs2124499 | C | G | 0.3718 | -0.0123 | 0.0017 | 3.40E-13 | 3.11E-05 | 24.45468657 |
| 180 | rs2143253 | A | G | 0.1189 | -0.0188 | 0.0026 | 1.10E-12 | 1.60E-05 | 10.95498365 |
| 181 | rs2162524 | C | T | 0.3321 | 0.0155 | 0.0018 | 4.10E-17 | 4.76E-05 | 32.89638005 |
| 182 | rs2163188 | C | G | 0.474 | 0.0131 | 0.0017 | 2.00E-14 | 4.31E-05 | 29.61121974 |
| 183 | rs2174307 | C | G | 0.4067 | 0.0121 | 0.0017 | 4.90E-12 | 3.56E-05 | 24.44925413 |
| 184 | rs217671 | G | A | 0.2719 | 0.0144 | 0.0019 | 1.30E-13 | 3.29E-05 | 22.74369618 |
| 185 | rs2228213 | A | G | 0.3481 | -0.0139 | 0.0017 | 4.60E-16 | 3.81E-05 | 30.34325936 |
| 186 | rs2235564 | T | C | 0.3466 | 0.0131 | 0.0018 | 3.70E-13 | 3.47E-05 | 23.99104004 |
| 187 | rs2246012 | C | T | 0.1628 | 0.0158 | 0.0022 | 3.10E-13 | 1.77E-05 | 14.06011948 |
| 188 | rs2283093 | T | C | 0.2066 | 0.0127 | 0.0021 | 3.10E-09 | 1.73E-05 | 11.99023329 |
| 189 | rs2284746 | G | C | 0.5238 | -0.0104 | 0.0017 | 1.40E-09 | 2.70E-05 | 18.6708537 |
| 190 | rs2285178 | C | T | 0.3109 | 0.0112 | 0.0019 | 9.40E-09 | 2.33E-05 | 14.88917317 |
| 191 | rs2306537 | G | A | 0.3092 | 0.0133 | 0.0019 | 8.70E-13 | 3.03E-05 | 20.93291805 |
| 192 | rs2307111 | C | T | 0.3962 | -0.0265 | 0.0016 | 1.60E-58 | 0.000165001 | 131.2683212 |
| 193 | rs2317299 | C | T | 0.5597 | -0.0106 | 0.0017 | 1.30E-09 | 2.83E-05 | 19.16279571 |
| 194 | rs2357760 | A | G | 0.6754 | 0.0145 | 0.0017 | 6.80E-17 | 4.03E-05 | 31.90025424 |
| 195 | rs2361988 | C | T | 0.2539 | -0.0155 | 0.002 | 5.20E-15 | 3.30E-05 | 22.75653844 |
| 196 | rs2367112 | G | T | 0.4919 | -0.0119 | 0.0016 | 2.30E-13 | 3.48E-05 | 27.65183748 |
| 197 | rs2411182 | A | G | 0.6943 | 0.0123 | 0.0019 | 7.70E-11 | 2.57E-05 | 17.79039269 |
| 198 | rs2423668 | C | T | 0.5505 | -0.0105 | 0.0019 | 2.80E-08 | 2.87E-05 | 15.11468886 |
| 199 | rs2425840 | C | A | 0.4059 | 0.0119 | 0.0018 | 1.60E-11 | 3.09E-05 | 21.07995442 |
| 200 | rs2429150 | C | A | 0.4164 | 0.0111 | 0.0018 | 2.70E-10 | 2.69E-05 | 18.48278356 |
| 201 | rs2479958 | G | A | 0.5075 | -0.0154 | 0.0018 | 1.50E-17 | 5.51E-05 | 36.5924362 |
| 202 | rs2543132 | C | G | 0.8134 | 0.0146 | 0.0022 | 5.00E-11 | 1.94E-05 | 13.36944338 |
| 203 | rs2600226 | T | C | 0.6697 | -0.0116 | 0.0019 | 3.70E-10 | 2.41E-05 | 16.49061407 |
| 204 | rs2605603 | A | G | 0.4887 | -0.0103 | 0.0016 | 2.50E-10 | 2.62E-05 | 20.71060979 |
| 205 | rs2608703 | A | C | 0.4546 | 0.0142 | 0.0017 | 1.90E-16 | 5.04E-05 | 34.59983466 |
| 206 | rs2643452 | A | T | 0.5448 | 0.0136 | 0.0017 | 4.70E-15 | 4.60E-05 | 31.74446612 |
| 207 | rs2693826 | A | G | 0.4421 | -0.0137 | 0.0017 | 2.00E-15 | 4.64E-05 | 32.03826929 |
| 208 | rs2694047 | G | A | 0.747 | 0.0188 | 0.002 | 3.90E-21 | 4.84E-05 | 33.40000874 |
| 209 | rs273504 | G | A | 0.4266 | 0.0153 | 0.0018 | 4.40E-18 | 5.12E-05 | 35.34820424 |
| 210 | rs2744974 | T | C | 0.338 | 0.0249 | 0.0018 | 1.40E-45 | 0.000108449 | 85.64546482 |
| 211 | rs2791653 | G | A | 0.7577 | -0.0141 | 0.0019 | 1.30E-13 | 2.54E-05 | 20.22188663 |
| 212 | rs2820311 | G | A | 0.3369 | 0.0235 | 0.0018 | 4.10E-38 | 0.000110071 | 76.1635712 |
| 213 | rs2832283 | A | G | 0.2208 | 0.0115 | 0.002 | 5.80E-09 | 1.44E-05 | 11.37676632 |
| 214 | rs2836964 | C | T | 0.3576 | -0.011 | 0.0018 | 1.30E-09 | 2.48E-05 | 17.15864174 |
| 215 | rs2861683 | C | A | 0.407 | -0.0144 | 0.0017 | 1.30E-16 | 5.01E-05 | 34.63592145 |
| 216 | rs2868975 | A | G | 0.178 | -0.0143 | 0.0023 | 2.20E-10 | 1.64E-05 | 11.31212184 |
| 217 | rs287104 | A | G | 0.6604 | 0.0115 | 0.0017 | 4.40E-11 | 2.61E-05 | 20.52640054 |
| 218 | rs2875762 | C | G | 0.2473 | 0.0139 | 0.002 | 1.20E-11 | 2.62E-05 | 17.98273595 |
| 219 | rs2907948 | A | G | 0.2427 | -0.0141 | 0.0019 | 1.30E-13 | 2.55E-05 | 20.24457797 |
| 220 | rs2931434 | T | C | 0.3168 | -0.0104 | 0.0018 | 1.40E-08 | 2.09E-05 | 14.45082107 |
| 221 | rs294704 | T | G | 0.7239 | -0.0113 | 0.0019 | 4.00E-09 | 2.05E-05 | 14.13944309 |
| 222 | rs3007105 | T | C | 0.4697 | 0.0142 | 0.0017 | 1.10E-17 | 4.42E-05 | 34.75914947 |
| 223 | rs326896 | T | C | 0.3925 | -0.0128 | 0.0018 | 2.80E-13 | 3.49E-05 | 24.11597258 |
| 224 | rs331966 | C | A | 0.3792 | 0.0112 | 0.0018 | 3.20E-10 | 2.65E-05 | 18.22851642 |
| 225 | rs33500 | T | C | 0.8082 | -0.0167 | 0.0022 | 4.30E-14 | 2.59E-05 | 17.8646708 |
| 226 | rs339991 | G | A | 0.5631 | 0.0124 | 0.0018 | 1.20E-12 | 3.40E-05 | 23.35121246 |
| 227 | rs349088 | A | C | 0.4976 | -0.0128 | 0.0017 | 1.80E-13 | 4.14E-05 | 28.34645884 |
| 228 | rs355777 | C | G | 0.4106 | 0.0153 | 0.0017 | 1.40E-18 | 5.68E-05 | 39.20735184 |
| 229 | rs3731695 | C | T | 0.5582 | 0.0116 | 0.0016 | 7.90E-13 | 3.27E-05 | 25.92594802 |
| 230 | rs3732084 | C | T | 0.6139 | 0.0107 | 0.0018 | 1.10E-09 | 2.42E-05 | 16.75171351 |
| 231 | rs3749897 | T | C | 0.4172 | 0.0122 | 0.0018 | 8.40E-12 | 3.50E-05 | 22.33995687 |
| 232 | rs3754963 | T | A | 0.2574 | -0.0123 | 0.002 | 3.30E-10 | 2.09E-05 | 14.45944219 |
| 233 | rs3772882 | A | C | 0.3661 | 0.0127 | 0.0018 | 6.60E-13 | 3.34E-05 | 23.10607377 |
| 234 | rs3800229 | T | G | 0.7123 | 0.0175 | 0.0018 | 1.40E-22 | 4.89E-05 | 38.74217729 |
| 235 | rs3800637 | C | T | 0.336 | 0.0115 | 0.0018 | 5.10E-10 | 2.67E-05 | 18.21370707 |
| 236 | rs3806114 | A | G | 0.6773 | -0.0113 | 0.0018 | 3.40E-10 | 2.21E-05 | 17.22782313 |
| 237 | rs3806572 | A | G | 0.2788 | -0.0145 | 0.0019 | 1.60E-14 | 3.41E-05 | 23.42182948 |
| 238 | rs3807645 | A | G | 0.221 | -0.0166 | 0.0021 | 2.40E-15 | 3.15E-05 | 21.51541468 |
| 239 | rs3814883 | T | C | 0.4764 | 0.0232 | 0.0017 | 1.10E-40 | 0.000135538 | 92.92597223 |
| 240 | rs3829849 | T | C | 0.3589 | 0.0098 | 0.0017 | 5.90E-09 | 1.93E-05 | 15.29293391 |
| 241 | rs38314 | A | G | 0.4912 | -0.012 | 0.0017 | 4.70E-12 | 3.61E-05 | 24.90660469 |
| 242 | rs3844598 | G | A | 0.521 | 0.0095 | 0.0017 | 3.80E-08 | 2.26E-05 | 15.58695003 |
| 243 | rs391300 | C | T | 0.6275 | -0.0119 | 0.0017 | 3.10E-12 | 2.90E-05 | 22.90749288 |
| 244 | rs3935648 | G | C | 0.2328 | -0.0125 | 0.0022 | 6.80E-09 | 1.83E-05 | 11.53195153 |
| 245 | rs3977755 | T | C | 0.2804 | -0.0135 | 0.0019 | 5.90E-13 | 2.79E-05 | 20.37372326 |
| 246 | rs40067 | A | G | 0.1713 | -0.0266 | 0.0023 | 7.10E-30 | 5.57E-05 | 37.9765246 |
| 247 | rs4012234 | G | T | 0.5924 | 0.0141 | 0.0018 | 9.90E-16 | 4.30E-05 | 29.63397004 |
| 248 | rs4072917 | A | G | 0.4694 | 0.0115 | 0.0018 | 6.90E-11 | 2.97E-05 | 20.33305451 |
| 249 | rs4148155 | G | A | 0.1127 | -0.0188 | 0.0026 | 5.00E-13 | 1.32E-05 | 10.45678109 |
| 250 | rs4148866 | T | C | 0.4068 | 0.0098 | 0.0018 | 4.00E-08 | 2.11E-05 | 14.30629331 |
| 251 | rs4237643 | G | T | 0.6938 | -0.0223 | 0.0019 | 4.30E-33 | 8.45E-05 | 58.53389921 |
| 252 | rs427943 | C | A | 0.5669 | 0.017 | 0.0017 | 7.30E-23 | 6.90E-05 | 49.1081265 |
| 253 | rs429343 | G | A | 0.5813 | -0.015 | 0.0017 | 6.80E-18 | 5.50E-05 | 37.90011884 |
| 254 | rs4307239 | G | A | 0.4578 | 0.0115 | 0.0017 | 3.90E-11 | 3.31E-05 | 22.71832075 |
| 255 | rs4358081 | C | A | 0.4631 | 0.0097 | 0.0017 | 1.50E-08 | 2.35E-05 | 16.19021952 |
| 256 | rs4430672 | C | T | 0.8004 | -0.0127 | 0.0022 | 3.90E-09 | 1.54E-05 | 10.64792847 |
| 257 | rs4482463 | A | C | 0.9213 | -0.0331 | 0.0033 | 2.80E-23 | 2.30E-05 | 14.58957066 |
| 258 | rs4516268 | A | C | 0.1925 | -0.0217 | 0.0021 | 5.20E-25 | 4.22E-05 | 33.19719294 |
| 259 | rs4518345 | A | G | 0.2842 | -0.0117 | 0.0019 | 1.00E-09 | 2.24E-05 | 15.42832411 |
| 260 | rs4556997 | A | C | 0.1349 | 0.0197 | 0.0024 | 6.90E-17 | 1.98E-05 | 15.72627058 |
| 261 | rs4639527 | G | A | 0.3012 | 0.0172 | 0.0019 | 3.30E-20 | 4.99E-05 | 34.49911512 |
| 262 | rs4660443 | T | C | 0.2218 | 0.0164 | 0.0021 | 6.80E-15 | 3.06E-05 | 21.05445053 |
| 263 | rs4740619 | C | T | 0.4521 | -0.0186 | 0.0016 | 2.30E-30 | 8.43E-05 | 66.95565018 |
| 264 | rs4757144 | A | G | 0.5878 | 0.0169 | 0.0018 | 5.60E-22 | 6.19E-05 | 42.71905029 |
| 265 | rs4783830 | A | G | 0.3074 | -0.0105 | 0.0019 | 2.40E-08 | 1.93E-05 | 13.00453468 |
| 266 | rs4786903 | G | A | 0.7368 | 0.0125 | 0.002 | 3.50E-10 | 2.23E-05 | 15.15074294 |
| 267 | rs4800191 | C | G | 0.6369 | 0.0103 | 0.0017 | 2.50E-09 | 2.16E-05 | 16.97901075 |
| 268 | rs4813619 | T | G | 0.5101 | -0.0108 | 0.0018 | 2.30E-09 | 2.89E-05 | 17.99311735 |
| 269 | rs4818225 | G | A | 0.6606 | 0.0117 | 0.0018 | 2.30E-10 | 2.75E-05 | 18.94601204 |
| 270 | rs4820408 | G | T | 0.592 | -0.0151 | 0.0017 | 2.10E-19 | 4.80E-05 | 38.11427522 |
| 271 | rs4842491 | T | C | 0.7138 | 0.0098 | 0.0018 | 4.00E-08 | 1.52E-05 | 12.11124616 |
| 272 | rs4858193 | C | T | 0.2779 | -0.0129 | 0.0019 | 1.60E-11 | 2.69E-05 | 18.50114214 |
| 273 | rs486359 | C | G | 0.4853 | 0.0112 | 0.0017 | 1.60E-11 | 2.81E-05 | 21.68421704 |
| 274 | rs4864201 | C | T | 0.6469 | -0.0141 | 0.0017 | 1.50E-16 | 3.95E-05 | 31.42833093 |
| 275 | rs4880341 | T | C | 0.5606 | -0.0118 | 0.0017 | 1.10E-11 | 3.44E-05 | 23.73684612 |
| 276 | rs4906908 | G | T | 0.5253 | 0.0103 | 0.0017 | 2.50E-09 | 2.65E-05 | 18.30810857 |
| 277 | rs491711 | C | A | 0.316 | -0.0115 | 0.0019 | 1.10E-09 | 2.31E-05 | 15.83690932 |
| 278 | rs4929923 | C | T | 0.6376 | 0.0181 | 0.0017 | 7.20E-27 | 6.59E-05 | 52.39059479 |
| 279 | rs4936175 | C | T | 0.4445 | 0.0122 | 0.0017 | 1.40E-12 | 3.67E-05 | 25.43444924 |
| 280 | rs4952843 | G | A | 0.3807 | -0.0131 | 0.0018 | 6.80E-14 | 3.61E-05 | 24.97617582 |
| 281 | rs4954638 | C | A | 0.2492 | -0.0118 | 0.002 | 2.90E-09 | 1.89E-05 | 13.0260656 |
| 282 | rs4968656 | G | A | 0.3216 | 0.0116 | 0.0019 | 8.20E-10 | 2.41E-05 | 16.2648412 |
| 283 | rs4981693 | A | G | 0.771 | 0.0206 | 0.002 | 6.90E-24 | 5.44E-05 | 37.46421654 |
| 284 | rs4986044 | T | C | 0.4687 | -0.0164 | 0.0016 | 3.30E-23 | 6.65E-05 | 52.32873792 |
| 285 | rs538579 | C | G | 0.3228 | 0.0137 | 0.0019 | 1.30E-13 | 3.30E-05 | 22.73146789 |
| 286 | rs559231 | T | G | 0.3956 | 0.0135 | 0.0018 | 2.40E-14 | 3.93E-05 | 26.89979955 |
| 287 | rs577525 | C | T | 0.5676 | 0.0166 | 0.0017 | 9.70E-22 | 6.78E-05 | 46.80632854 |
| 288 | rs592483 | T | C | 0.5716 | -0.0147 | 0.0017 | 2.00E-18 | 4.68E-05 | 36.62079217 |
| 289 | rs6011457 | A | T | 0.4975 | -0.0116 | 0.0017 | 2.70E-11 | 3.37E-05 | 23.28041206 |
| 290 | rs6235 | G | C | 0.2702 | 0.0175 | 0.0019 | 1.50E-19 | 4.84E-05 | 33.45860624 |
| 291 | rs6443750 | C | T | 0.8068 | 0.0148 | 0.0021 | 3.20E-12 | 1.99E-05 | 15.48443585 |
| 292 | rs6448587 | C | A | 0.1891 | -0.0167 | 0.0023 | 2.30E-13 | 2.34E-05 | 16.16869612 |
| 293 | rs645040 | T | G | 0.7762 | 0.0171 | 0.002 | 2.50E-18 | 3.19E-05 | 25.39853801 |
| 294 | rs6461115 | G | A | 0.2285 | -0.0144 | 0.0019 | 1.20E-13 | 2.56E-05 | 20.25255988 |
| 295 | rs6471941 | A | G | 0.1684 | 0.0156 | 0.0021 | 3.10E-13 | 1.95E-05 | 15.45626414 |
| 296 | rs6500208 | A | G | 0.2006 | 0.014 | 0.002 | 4.10E-12 | 2.01E-05 | 15.71552037 |
| 297 | rs6512302 | C | G | 0.7511 | 0.0142 | 0.002 | 2.10E-12 | 2.75E-05 | 18.8486399 |
| 298 | rs6561943 | T | C | 0.2595 | 0.0119 | 0.0019 | 4.20E-10 | 1.90E-05 | 15.07600574 |
| 299 | rs6587552 | G | A | 0.7591 | -0.0173 | 0.002 | 1.60E-17 | 3.97E-05 | 27.36616706 |
| 300 | rs6593688 | G | A | 0.3733 | 0.0137 | 0.0018 | 8.60E-15 | 3.92E-05 | 27.10563371 |
| 301 | rs6595205 | G | C | 0.5305 | -0.0114 | 0.0016 | 2.00E-12 | 3.18E-05 | 25.28910431 |
| 302 | rs6673081 | C | T | 0.5534 | -0.01 | 0.0018 | 1.80E-08 | 2.25E-05 | 15.25637491 |
| 303 | rs6692586 | G | A | 0.832 | -0.0192 | 0.0023 | 1.10E-16 | 2.82E-05 | 19.48140959 |
| 304 | rs6764533 | A | G | 0.359 | 0.0116 | 0.0018 | 1.40E-10 | 2.77E-05 | 19.11455541 |
| 305 | rs6772756 | G | A | 0.3372 | -0.0104 | 0.0019 | 4.00E-08 | 1.96E-05 | 13.39265853 |
| 306 | rs6785245 | C | T | 0.3969 | 0.0132 | 0.0017 | 4.00E-14 | 4.17E-05 | 28.8647165 |
| 307 | rs6815910 | A | T | 0.5435 | -0.0128 | 0.0017 | 1.40E-13 | 4.08E-05 | 28.1325363 |
| 308 | rs6841761 | T | G | 0.5252 | -0.0131 | 0.0016 | 6.40E-16 | 4.21E-05 | 33.43376255 |
| 309 | rs685870 | C | T | 0.7035 | 0.012 | 0.0019 | 2.40E-10 | 2.42E-05 | 16.64114948 |
| 310 | rs6985109 | A | G | 0.5338 | -0.0177 | 0.0017 | 1.50E-26 | 6.80E-05 | 53.95826084 |
| 311 | rs7025938 | G | C | 0.3187 | 0.0166 | 0.0019 | 3.70E-19 | 4.79E-05 | 33.14965271 |
| 312 | rs7037266 | A | C | 0.3739 | -0.0112 | 0.0018 | 3.50E-10 | 2.62E-05 | 18.12718332 |
| 313 | rs705217 | G | T | 0.3652 | -0.0102 | 0.0018 | 9.30E-09 | 2.16E-05 | 14.88884966 |
| 314 | rs7084454 | A | G | 0.335 | 0.0193 | 0.0019 | 4.00E-25 | 6.78E-05 | 45.9760873 |
| 315 | rs7102454 | C | T | 0.3435 | 0.0158 | 0.0018 | 2.40E-18 | 5.03E-05 | 34.75211266 |
| 316 | rs7124681 | A | C | 0.4133 | 0.0263 | 0.0016 | 3.20E-58 | 0.000164724 | 131.0549634 |
| 317 | rs7144011 | T | G | 0.2136 | 0.0282 | 0.002 | 5.20E-47 | 8.41E-05 | 66.79568513 |
| 318 | rs7172627 | G | A | 0.4719 | 0.0117 | 0.0017 | 1.10E-11 | 3.42E-05 | 23.60932731 |
| 319 | rs7181498 | C | T | 0.6309 | -0.0163 | 0.0018 | 1.00E-19 | 5.53E-05 | 38.19332905 |
| 320 | rs7196720 | C | T | 0.5068 | -0.0129 | 0.0017 | 7.30E-14 | 4.17E-05 | 28.78645002 |
| 321 | rs7222349 | A | G | 0.3441 | 0.0115 | 0.0018 | 3.30E-10 | 2.66E-05 | 18.42524514 |
| 322 | rs7239575 | C | T | 0.4832 | -0.0202 | 0.0017 | 7.40E-32 | 0.000101855 | 70.52243595 |
| 323 | rs7318817 | T | C | 0.6071 | -0.0155 | 0.0018 | 2.70E-18 | 5.11E-05 | 35.37623353 |
| 324 | rs7334078 | C | T | 0.2882 | -0.0121 | 0.0019 | 2.20E-10 | 2.42E-05 | 16.64005398 |
| 325 | rs7358465 | T | C | 0.6781 | 0.0103 | 0.0019 | 3.00E-08 | 1.87E-05 | 12.82976823 |
| 326 | rs7488867 | T | C | 0.2639 | -0.0204 | 0.002 | 8.40E-24 | 6.36E-05 | 40.42339584 |
| 327 | rs7519259 | A | G | 0.5356 | 0.0125 | 0.0017 | 3.80E-13 | 3.95E-05 | 26.89681322 |
| 328 | rs754635 | G | C | 0.8873 | 0.0198 | 0.0027 | 2.20E-13 | 1.56E-05 | 10.75555322 |
| 329 | rs7557796 | C | T | 0.6524 | -0.016 | 0.0018 | 2.30E-19 | 5.18E-05 | 35.83768056 |
| 330 | rs756717 | A | G | 0.3973 | -0.0148 | 0.0017 | 5.40E-18 | 4.70E-05 | 36.29899789 |
| 331 | rs7626079 | T | C | 0.3434 | 0.011 | 0.0018 | 1.60E-09 | 2.43E-05 | 16.8415024 |
| 332 | rs7637852 | G | A | 0.6951 | -0.0139 | 0.0019 | 1.70E-13 | 3.28E-05 | 22.68663549 |
| 333 | rs7640424 | T | C | 0.2969 | -0.0136 | 0.0018 | 2.30E-14 | 3.01E-05 | 23.83428299 |
| 334 | rs765875 | T | C | 0.4808 | -0.0121 | 0.0017 | 3.00E-12 | 3.66E-05 | 25.29395125 |
| 335 | rs7683836 | A | G | 0.5405 | -0.0114 | 0.0017 | 6.30E-11 | 3.25E-05 | 22.33757001 |
| 336 | rs7685048 | T | C | 0.4654 | -0.0101 | 0.0017 | 4.10E-09 | 2.54E-05 | 17.56467006 |
| 337 | rs768840 | A | G | 0.4183 | 0.0114 | 0.0018 | 2.00E-10 | 2.88E-05 | 19.52058586 |
| 338 | rs769449 | A | G | 0.1161 | -0.0254 | 0.0027 | 2.30E-20 | 3.20E-05 | 18.16425701 |
| 339 | rs7694732 | G | A | 0.4378 | -0.0099 | 0.0017 | 8.70E-09 | 2.42E-05 | 16.69469085 |
| 340 | rs7703576 | C | T | 0.2885 | 0.0103 | 0.0019 | 4.80E-08 | 1.75E-05 | 12.06491572 |
| 341 | rs7724675 | A | G | 0.2238 | -0.0119 | 0.0021 | 9.50E-09 | 1.61E-05 | 11.15641848 |
| 342 | rs7730004 | T | C | 0.6693 | 0.0148 | 0.0018 | 9.10E-16 | 4.34E-05 | 29.92822843 |
| 343 | rs7730898 | A | G | 0.729 | 0.0168 | 0.0018 | 4.50E-20 | 4.34E-05 | 34.42057522 |
| 344 | rs7761673 | A | T | 0.2058 | -0.0126 | 0.0021 | 1.90E-09 | 1.70E-05 | 11.76830411 |
| 345 | rs7780752 | C | T | 0.36 | 0.0139 | 0.0018 | 1.00E-14 | 3.98E-05 | 27.47976905 |
| 346 | rs7788008 | A | G | 0.4445 | -0.0157 | 0.0017 | 1.10E-19 | 6.10E-05 | 42.12234337 |
| 347 | rs7819514 | A | G | 0.3216 | -0.0107 | 0.0018 | 5.70E-09 | 2.25E-05 | 15.41923859 |
| 348 | rs7826312 | C | T | 0.5879 | 0.0104 | 0.0017 | 4.90E-10 | 2.31E-05 | 18.13484419 |
| 349 | rs7844647 | C | T | 0.2681 | -0.0123 | 0.0018 | 2.80E-11 | 2.31E-05 | 18.3253679 |
| 350 | rs7869771 | C | A | 0.2647 | -0.014 | 0.0019 | 4.90E-13 | 3.11E-05 | 21.13535776 |
| 351 | rs7871866 | C | G | 0.1531 | 0.0187 | 0.0024 | 2.30E-14 | 2.30E-05 | 15.74369913 |
| 352 | rs7925214 | T | C | 0.5133 | 0.0147 | 0.0018 | 4.40E-17 | 4.92E-05 | 33.32516759 |
| 353 | rs7970953 | A | G | 0.29 | 0.0135 | 0.0018 | 9.80E-14 | 2.94E-05 | 23.16437181 |
| 354 | rs7983065 | T | C | 0.4503 | -0.0148 | 0.0017 | 8.90E-18 | 5.43E-05 | 37.52369491 |
| 355 | rs7998796 | G | A | 0.3373 | 0.0105 | 0.0018 | 1.10E-08 | 2.21E-05 | 15.21266208 |
| 356 | rs8027205 | G | C | 0.3967 | -0.0108 | 0.0018 | 1.40E-09 | 2.51E-05 | 17.23207869 |
| 357 | rs8036040 | A | C | 0.4932 | 0.0109 | 0.0017 | 2.70E-10 | 2.97E-05 | 20.55211312 |
| 358 | rs8047395 | A | G | 0.5061 | 0.0642 | 0.0017 | 1.00E-200 | 0.000903816 | 713.6235459 |
| 359 | rs806600 | G | A | 0.475 | -0.0095 | 0.0017 | 3.30E-08 | 2.25E-05 | 15.57545703 |
| 360 | rs8071182 | A | G | 0.1735 | 0.0133 | 0.0022 | 2.10E-09 | 1.36E-05 | 10.48177971 |
| 361 | rs8090983 | G | A | 0.3314 | 0.0118 | 0.0018 | 2.00E-10 | 2.79E-05 | 19.04490115 |
| 362 | rs8097783 | A | G | 0.07554 | -0.0389 | 0.0031 | 7.20E-36 | 2.76E-05 | 21.99286618 |
| 363 | rs8123881 | G | A | 0.1299 | 0.0196 | 0.0024 | 4.40E-16 | 1.90E-05 | 15.07665983 |
| 364 | rs8181823 | C | A | 0.7614 | 0.0127 | 0.002 | 4.10E-10 | 2.12E-05 | 14.65104847 |
| 365 | rs818524 | C | T | 0.6939 | 0.0106 | 0.0019 | 3.40E-08 | 1.97E-05 | 13.22214802 |
| 366 | rs8192675 | C | T | 0.2888 | 0.0152 | 0.0018 | 1.40E-17 | 3.68E-05 | 29.29381931 |
| 367 | rs825688 | T | C | 0.456 | -0.0095 | 0.0017 | 4.70E-08 | 2.26E-05 | 15.49357528 |
| 368 | rs845084 | A | G | 0.2678 | 0.014 | 0.002 | 1.30E-12 | 2.80E-05 | 19.21663236 |
| 369 | rs852056 | C | T | 0.7584 | -0.0128 | 0.002 | 1.80E-10 | 2.17E-05 | 15.01043799 |
| 370 | rs865809 | G | A | 0.7678 | -0.0127 | 0.002 | 5.40E-10 | 2.09E-05 | 14.37790366 |
| 371 | rs872281 | T | C | 0.1728 | -0.0151 | 0.0023 | 4.70E-11 | 1.80E-05 | 12.32222185 |
| 372 | rs876605 | G | A | 0.7352 | -0.0108 | 0.002 | 3.40E-08 | 1.64E-05 | 11.35394693 |
| 373 | rs879620 | T | C | 0.6179 | 0.0231 | 0.0018 | 5.30E-38 | 0.000112974 | 77.77714245 |
| 374 | rs895330 | G | C | 0.1924 | -0.0201 | 0.0023 | 5.50E-19 | 3.47E-05 | 23.73458327 |
| 375 | rs902695 | A | G | 0.4798 | -0.0103 | 0.0017 | 2.20E-09 | 2.70E-05 | 18.32515434 |
| 376 | rs9294260 | A | G | 0.4731 | 0.0147 | 0.0016 | 1.80E-19 | 5.37E-05 | 42.085071 |
| 377 | rs9300422 | G | A | 0.6903 | -0.0103 | 0.0018 | 4.00E-09 | 1.76E-05 | 14.00054911 |
| 378 | rs930295 | C | A | 0.8417 | -0.0211 | 0.0023 | 1.00E-19 | 3.25E-05 | 22.42798846 |
| 379 | rs9304665 | A | T | 0.7633 | 0.0229 | 0.002 | 2.90E-29 | 6.87E-05 | 47.37651062 |
| 380 | rs934224 | T | C | 0.7399 | 0.0107 | 0.002 | 4.70E-08 | 1.59E-05 | 11.01682862 |
| 381 | rs9362662 | G | A | 0.5201 | -0.0112 | 0.0017 | 1.20E-10 | 3.17E-05 | 21.66797324 |
| 382 | rs9367368 | C | T | 0.3033 | -0.0121 | 0.0018 | 1.00E-11 | 2.43E-05 | 19.09780195 |
| 383 | rs9408882 | A | G | 0.4594 | -0.0093 | 0.0016 | 1.30E-08 | 2.11E-05 | 16.78151022 |
| 384 | rs946824 | C | T | 0.859 | -0.0206 | 0.0026 | 1.10E-15 | 2.20E-05 | 15.20681741 |
| 385 | rs947612 | A | G | 0.7516 | -0.0116 | 0.002 | 5.60E-09 | 1.81E-05 | 12.5611953 |
| 386 | rs9478671 | G | A | 0.2087 | 0.012 | 0.0021 | 1.70E-08 | 1.57E-05 | 10.78507223 |
| 387 | rs9538162 | C | T | 0.4138 | -0.0156 | 0.0018 | 4.80E-19 | 5.28E-05 | 36.4411563 |
| 388 | rs9547153 | G | A | 0.3839 | 0.0098 | 0.0017 | 8.70E-09 | 2.03E-05 | 15.72031738 |
| 389 | rs9571687 | A | C | 0.329 | -0.0129 | 0.0018 | 2.80E-12 | 3.28E-05 | 22.67753366 |
| 390 | rs9615905 | T | C | 0.45 | 0.011 | 0.0017 | 2.70E-10 | 3.00E-05 | 20.72547571 |
| 391 | rs962273 | C | T | 0.7057 | 0.0137 | 0.0019 | 2.60E-13 | 3.12E-05 | 21.59666022 |
| 392 | rs9650755 | G | A | 0.2664 | 0.0154 | 0.002 | 2.80E-15 | 3.35E-05 | 23.17492268 |
| 393 | rs977747 | G | T | 0.5949 | -0.0169 | 0.0017 | 1.30E-24 | 6.00E-05 | 47.63616045 |
| 394 | rs9783858 | T | C | 0.5191 | 0.0091 | 0.0017 | 3.30E-08 | 1.86E-05 | 14.30631149 |
| 395 | rs9806742 | A | G | 0.8826 | 0.0208 | 0.0026 | 1.40E-15 | 1.92E-05 | 13.26322243 |
| 396 | rs9816226 | T | A | 0.8199 | 0.0323 | 0.0021 | 1.60E-52 | 8.98E-05 | 69.8729244 |
| 397 | rs9845966 | G | T | 0.5479 | -0.0105 | 0.0017 | 2.50E-10 | 2.43E-05 | 18.89974703 |
| 398 | rs987237 | G | A | 0.1803 | 0.0409 | 0.0021 | 9.30E-84 | 0.000140925 | 112.1369655 |
| 399 | rs9926784 | C | T | 0.1822 | -0.0258 | 0.0021 | 9.90E-35 | 5.70E-05 | 44.98315758 |
| 400 | rs9927848 | A | C | 0.7326 | -0.0122 | 0.002 | 6.40E-10 | 2.12E-05 | 14.57893951 |
| 401 | rs9951619 | G | T | 0.7643 | 0.0156 | 0.002 | 1.40E-15 | 2.84E-05 | 21.92067081 |
| 402 | rs998732 | G | A | 0.1578 | -0.0171 | 0.0022 | 2.00E-14 | 2.02E-05 | 16.05856677 |
| 403 | rs999889 | A | G | 0.2818 | -0.0108 | 0.0019 | 1.40E-08 | 1.89E-05 | 13.0786724 |
| **Exposures/No.** | **SNP** | **A1** | **A2** | **Eaf** | **Beta** | **SE** | **P** | **R^2^** | **F-statistic** |
| **Body fat percentage ^d^** |  |  |  |  |  |  |  |  |  |
| 1 | rs10050620 | T | C | 0.325382 | -0.0103073 | 0.00164616 | 3.80E-10 | 3.79E-05 | 17.21241151 |
| 2 | rs10100245 | A | G | 0.564497 | 0.0152146 | 0.00155645 | 1.40E-22 | 0.000103341 | 46.98686695 |
| 3 | rs1013293 | A | G | 0.430286 | -0.0139271 | 0.00155866 | 4.10E-19 | 8.61E-05 | 39.14697374 |
| 4 | rs10144067 | T | C | 0.59128 | 0.0100925 | 0.00158231 | 1.80E-10 | 4.33E-05 | 19.66438276 |
| 5 | rs10146997 | G | A | 0.221871 | 0.018731 | 0.00185697 | 6.30E-24 | 7.73E-05 | 35.13386601 |
| 6 | rs10209821 | T | C | 0.342612 | 0.0123638 | 0.0016188 | 2.20E-14 | 5.78E-05 | 26.27816773 |
| 7 | rs10245306 | C | G | 0.685981 | 0.00917484 | 0.00167492 | 4.30E-08 | 2.84E-05 | 12.92758224 |
| 8 | rs10259620 | G | A | 0.787159 | -0.0139877 | 0.00189659 | 1.60E-13 | 4.01E-05 | 18.2267383 |
| 9 | rs1038088 | G | T | 0.519335 | 0.0125471 | 0.00154189 | 4.00E-16 | 7.27E-05 | 33.0620436 |
| 10 | rs10423928 | A | T | 0.194436 | -0.0232502 | 0.00194747 | 7.40E-33 | 9.82E-05 | 44.65389027 |
| 11 | rs10496731 | G | T | 0.374905 | -0.0115311 | 0.00158686 | 3.70E-13 | 5.44E-05 | 24.7504578 |
| 12 | rs10510025 | T | C | 0.24708 | 0.0129238 | 0.00179232 | 5.60E-13 | 4.26E-05 | 19.34560635 |
| 13 | rs10513935 | A | G | 0.301501 | -0.00942576 | 0.00168416 | 2.20E-08 | 2.90E-05 | 13.19353861 |
| 14 | rs10756798 | T | C | 0.642437 | -0.0140265 | 0.00160866 | 2.80E-18 | 7.68E-05 | 34.9312942 |
| 15 | rs1078455 | C | T | 0.309511 | 0.00964481 | 0.00167852 | 9.10E-09 | 3.10E-05 | 14.11265522 |
| 16 | rs10788497 | C | G | 0.499616 | 0.0103873 | 0.00154427 | 1.70E-11 | 4.98E-05 | 22.62290274 |
| 17 | rs10854853 | T | G | 0.456784 | 0.0104441 | 0.00155077 | 1.60E-11 | 4.95E-05 | 22.5102483 |
| 18 | rs10896012 | C | T | 0.217394 | 0.0115223 | 0.00187372 | 7.80E-10 | 2.83E-05 | 12.86767478 |
| 19 | rs10959841 | C | T | 0.387368 | -0.00911255 | 0.00158643 | 9.20E-09 | 3.44E-05 | 15.66045892 |
| 20 | rs10999460 | T | C | 0.265521 | 0.016885 | 0.00175383 | 6.10E-22 | 7.95E-05 | 36.15497123 |
| 21 | rs11022718 | T | C | 0.202968 | -0.0107504 | 0.00193009 | 2.50E-08 | 2.21E-05 | 10.03772124 |
| 22 | rs11030016 | T | C | 0.739777 | 0.0132564 | 0.001761 | 5.20E-14 | 4.80E-05 | 21.81865221 |
| 23 | rs11030108 | G | A | 0.679962 | -0.0185028 | 0.00165098 | 3.80E-29 | 0.000120239 | 54.67109626 |
| 24 | rs11079849 | T | C | 0.328562 | -0.0130416 | 0.00164632 | 2.30E-15 | 6.09E-05 | 27.68923953 |
| 25 | rs11105842 | A | G | 0.367542 | -0.0101643 | 0.00160647 | 2.50E-10 | 4.09E-05 | 18.61205122 |
| 26 | rs11119208 | G | A | 0.614902 | -0.00965759 | 0.00157945 | 9.70E-10 | 3.89E-05 | 17.70712008 |
| 27 | rs11122450 | G | T | 0.611689 | -0.0101571 | 0.00157943 | 1.30E-10 | 4.32E-05 | 19.64698473 |
| 28 | rs11129660 | T | C | 0.209783 | 0.0110275 | 0.00189369 | 5.80E-09 | 2.47E-05 | 11.24324946 |
| 29 | rs11150745 | G | A | 0.317696 | -0.0126783 | 0.00166019 | 2.20E-14 | 5.56E-05 | 25.28415026 |
| 30 | rs11205303 | C | T | 0.406646 | 0.018328 | 0.00156672 | 1.30E-31 | 0.00014526 | 66.0494084 |
| 31 | rs11208779 | C | G | 0.528878 | 0.010317 | 0.00154445 | 2.40E-11 | 4.89E-05 | 22.23808418 |
| 32 | rs11222371 | T | C | 0.409234 | 0.0118153 | 0.00156853 | 5.00E-14 | 6.03E-05 | 27.4375509 |
| 33 | rs11245344 | T | C | 0.571137 | 0.00937385 | 0.00156054 | 1.90E-09 | 3.89E-05 | 17.67624591 |
| 34 | rs112852122 | A | G | 0.157829 | -0.0164403 | 0.00215064 | 2.10E-14 | 3.42E-05 | 15.53509991 |
| 35 | rs11343 | G | T | 0.561331 | 0.00867343 | 0.00158474 | 4.40E-08 | 3.24E-05 | 14.75244027 |
| 36 | rs113503736 | G | T | 0.223372 | -0.0102974 | 0.00185568 | 2.90E-08 | 2.35E-05 | 10.68388144 |
| 37 | rs11538 | G | A | 0.171838 | 0.0121586 | 0.00204591 | 2.80E-09 | 2.21E-05 | 10.0523196 |
| 38 | rs11619393 | C | T | 0.144968 | 0.0146332 | 0.00219293 | 2.50E-11 | 2.43E-05 | 11.03880916 |
| 39 | rs11619722 | C | T | 0.300933 | -0.0108221 | 0.00168444 | 1.30E-10 | 3.82E-05 | 17.36781903 |
| 40 | rs11664106 | T | A | 0.373948 | 0.010994 | 0.00163476 | 1.80E-11 | 4.66E-05 | 21.17743692 |
| 41 | rs11664848 | G | C | 0.659616 | 0.00965173 | 0.00163558 | 3.60E-09 | 3.44E-05 | 15.63759957 |
| 42 | rs11666808 | C | T | 0.625293 | -0.0177091 | 0.00160075 | 1.90E-28 | 0.000126151 | 57.35934827 |
| 43 | rs11677541 | A | G | 0.535271 | -0.00923656 | 0.00154968 | 2.50E-09 | 3.89E-05 | 17.67481648 |
| 44 | rs11786089 | G | A | 0.459661 | 0.0112967 | 0.00154993 | 3.10E-13 | 5.80E-05 | 26.38981151 |
| 45 | rs11852419 | T | A | 0.261669 | 0.0106498 | 0.00175622 | 1.30E-09 | 3.13E-05 | 14.20921557 |
| 46 | rs11855853 | T | C | 0.269414 | -0.0103933 | 0.00174766 | 2.70E-09 | 3.06E-05 | 13.92277884 |
| 47 | rs11866219 | C | A | 0.583787 | -0.0158787 | 0.00158501 | 1.30E-23 | 0.000107277 | 48.77649752 |
| 48 | rs12053559 | G | T | 0.471201 | 0.00856437 | 0.00154317 | 2.90E-08 | 3.38E-05 | 15.34980548 |
| 49 | rs12072739 | G | A | 0.224584 | 0.0134145 | 0.00184627 | 3.70E-13 | 4.04E-05 | 18.38729205 |
| 50 | rs12375196 | A | C | 0.424277 | 0.0102996 | 0.00156914 | 5.20E-11 | 4.63E-05 | 21.04886057 |
| 51 | rs12376870 | A | G | 0.237663 | -0.0110309 | 0.00181676 | 1.30E-09 | 2.94E-05 | 13.35906911 |
| 52 | rs12402939 | C | A | 0.392363 | -0.0101759 | 0.00157772 | 1.10E-10 | 4.36E-05 | 19.836502 |
| 53 | rs12432026 | G | T | 0.540113 | 0.00996462 | 0.00154621 | 1.20E-10 | 4.54E-05 | 20.63327119 |
| 54 | rs12441543 | A | G | 0.287112 | 0.0114053 | 0.0017089 | 2.50E-11 | 4.01E-05 | 18.23468559 |
| 55 | rs12462975 | A | G | 0.32959 | 0.0123462 | 0.00165332 | 8.20E-14 | 5.42E-05 | 24.64443772 |
| 56 | rs12475388 | A | G | 0.485685 | -0.00988584 | 0.00154662 | 1.60E-10 | 4.49E-05 | 20.41227337 |
| 57 | rs12538435 | G | A | 0.261821 | -0.0109846 | 0.00175319 | 3.70E-10 | 3.34E-05 | 15.17467045 |
| 58 | rs12619178 | T | C | 0.400829 | -0.0144544 | 0.0015682 | 3.00E-20 | 8.98E-05 | 40.81071727 |
| 59 | rs12628603 | A | G | 0.617054 | -0.00959714 | 0.00159107 | 1.60E-09 | 3.78E-05 | 17.19532175 |
| 60 | rs12658319 | T | C | 0.299121 | 0.0107194 | 0.0016852 | 2.00E-10 | 3.73E-05 | 16.96572006 |
| 61 | rs12670456 | G | A | 0.301754 | 0.00941089 | 0.00168003 | 2.10E-08 | 2.91E-05 | 13.22298573 |
| 62 | rs12724928 | C | T | 0.20486 | -0.0146759 | 0.00190484 | 1.30E-14 | 4.25E-05 | 19.33925821 |
| 63 | rs1284373 | T | C | 0.199214 | -0.0108576 | 0.00192322 | 1.60E-08 | 2.24E-05 | 10.16913164 |
| 64 | rs12890931 | G | T | 0.362416 | 0.0120832 | 0.00161524 | 7.40E-14 | 5.69E-05 | 25.8635044 |
| 65 | rs12926311 | C | G | 0.353546 | -0.00918915 | 0.00161862 | 1.40E-08 | 3.24E-05 | 14.73284402 |
| 66 | rs13026103 | A | G | 0.74098 | 0.00961952 | 0.00175567 | 4.30E-08 | 2.53E-05 | 11.52391127 |
| 67 | rs13132853 | G | A | 0.360225 | -0.00936528 | 0.00161107 | 6.10E-09 | 3.43E-05 | 15.57603057 |
| 68 | rs1322842 | G | A | 0.609473 | -0.0114888 | 0.00158746 | 4.60E-13 | 5.48E-05 | 24.93453056 |
| 69 | rs13249935 | C | T | 0.366657 | 0.00954133 | 0.00161413 | 3.40E-09 | 3.57E-05 | 16.22868603 |
| 70 | rs13292699 | C | A | 0.433725 | -0.0200044 | 0.0015583 | 1.00E-37 | 0.000178057 | 80.96478824 |
| 71 | rs13389219 | T | C | 0.392441 | 0.0170899 | 0.00157313 | 1.70E-27 | 0.000123789 | 56.28523687 |
| 72 | rs13436840 | T | C | 0.265069 | 0.0136415 | 0.0017496 | 6.30E-15 | 5.21E-05 | 23.68661126 |
| 73 | rs1350429 | G | A | 0.480306 | 0.00973719 | 0.00154554 | 3.00E-10 | 4.36E-05 | 19.81616086 |
| 74 | rs1377184 | T | A | 0.748963 | 0.0127442 | 0.00178122 | 8.40E-13 | 4.23E-05 | 19.25017157 |
| 75 | rs1421334 | C | A | 0.54884 | -0.0113003 | 0.00155717 | 4.00E-13 | 5.74E-05 | 26.08178037 |
| 76 | rs1453055 | A | G | 0.273355 | 0.0126532 | 0.00173398 | 2.90E-13 | 4.65E-05 | 21.1548613 |
| 77 | rs1456014 | G | A | 0.205602 | -0.0150933 | 0.00190234 | 2.10E-15 | 4.52E-05 | 20.56389297 |
| 78 | rs1469084 | G | A | 0.545742 | 0.00878996 | 0.00154865 | 1.40E-08 | 3.51E-05 | 15.97351707 |
| 79 | rs1475860 | C | G | 0.505368 | 0.00875321 | 0.00155205 | 1.70E-08 | 3.50E-05 | 15.90217408 |
| 80 | rs1503526 | C | T | 0.47999 | 0.0113767 | 0.00154191 | 1.60E-13 | 5.98E-05 | 27.17768703 |
| 81 | rs1559900 | T | C | 0.286141 | 0.00986943 | 0.00170532 | 7.10E-09 | 3.01E-05 | 13.6837792 |
| 82 | rs1568488 | C | G | 0.594863 | 0.0117289 | 0.00158106 | 1.20E-13 | 5.83E-05 | 26.52712318 |
| 83 | rs1605898 | A | T | 0.837607 | 0.0129238 | 0.00209124 | 6.40E-10 | 2.29E-05 | 10.39006623 |
| 84 | rs1624064 | C | T | 0.420353 | -0.0106391 | 0.00156343 | 1.00E-11 | 4.96E-05 | 22.56733552 |
| 85 | rs17055384 | T | C | 0.182952 | -0.0123469 | 0.00200425 | 7.30E-10 | 2.50E-05 | 11.34581592 |
| 86 | rs17172722 | T | C | 0.418942 | -0.010769 | 0.0015641 | 5.80E-12 | 5.08E-05 | 23.0805015 |
| 87 | rs1724557 | A | C | 0.586755 | -0.0110497 | 0.0015726 | 2.10E-12 | 5.27E-05 | 23.94306777 |
| 88 | rs17522122 | T | G | 0.471197 | 0.0126085 | 0.00155155 | 4.40E-16 | 7.24E-05 | 32.91177042 |
| 89 | rs17639996 | A | G | 0.150071 | -0.0143309 | 0.00216477 | 3.60E-11 | 2.46E-05 | 11.17999473 |
| 90 | rs17704028 | T | C | 0.147192 | -0.0146575 | 0.00217921 | 1.70E-11 | 2.50E-05 | 11.35784784 |
| 91 | rs17770336 | T | C | 0.322448 | 0.0154638 | 0.00164622 | 5.80E-21 | 8.48E-05 | 38.55883801 |
| 92 | rs17820010 | G | T | 0.295596 | 0.0124473 | 0.00168847 | 1.70E-13 | 4.98E-05 | 22.63256037 |
| 93 | rs1782508 | G | C | 0.655489 | -0.00913519 | 0.00162015 | 1.70E-08 | 3.16E-05 | 14.35935796 |
| 94 | rs1787013 | C | T | 0.450716 | 0.00961178 | 0.00155008 | 5.60E-10 | 4.19E-05 | 19.03906491 |
| 95 | rs1801282 | G | C | 0.119535 | 0.0301151 | 0.0023675 | 4.60E-37 | 7.49E-05 | 34.06092576 |
| 96 | rs1808629 | A | G | 0.685441 | -0.01632 | 0.00166697 | 1.20E-22 | 9.09E-05 | 41.33562462 |
| 97 | rs1813039 | A | G | 0.709746 | 0.0103333 | 0.00170753 | 1.40E-09 | 3.32E-05 | 15.08915978 |
| 98 | rs1861410 | T | C | 0.555344 | -0.0135852 | 0.0015523 | 2.10E-18 | 8.32E-05 | 37.82955389 |
| 99 | rs1945160 | A | G | 0.375871 | -0.00962166 | 0.0016001 | 1.80E-09 | 3.73E-05 | 16.96536607 |
| 100 | rs1964675 | T | C | 0.711087 | 0.0096786 | 0.00170107 | 1.30E-08 | 2.93E-05 | 13.30182797 |
| 101 | rs1991002 | G | T | 0.495176 | -0.0100728 | 0.00154583 | 7.20E-11 | 4.67E-05 | 21.22877352 |
| 102 | rs2002023 | T | C | 0.409059 | 0.0102433 | 0.00157002 | 6.80E-11 | 4.53E-05 | 20.58008832 |
| 103 | rs2008018 | A | G | 0.322077 | 0.0110009 | 0.00164659 | 2.40E-11 | 4.29E-05 | 19.4927123 |
| 104 | rs2034946 | G | T | 0.208834 | 0.0119487 | 0.00190099 | 3.30E-10 | 2.87E-05 | 13.05542544 |
| 105 | rs2108635 | G | A | 0.338522 | 0.00983786 | 0.00163453 | 1.80E-09 | 3.57E-05 | 16.22412822 |
| 106 | rs2165991 | G | A | 0.253707 | 0.0117936 | 0.00178131 | 3.60E-11 | 3.65E-05 | 16.59967311 |
| 107 | rs2172131 | C | T | 0.578543 | -0.0110832 | 0.00156255 | 1.30E-12 | 5.40E-05 | 24.53595304 |
| 108 | rs2178899 | T | A | 0.128827 | -0.0191217 | 0.002297 | 8.50E-17 | 3.42E-05 | 15.55554585 |
| 109 | rs2182717 | A | G | 0.691021 | 0.010121 | 0.00167361 | 1.50E-09 | 3.44E-05 | 15.61714123 |
| 110 | rs2190788 | T | G | 0.319702 | 0.0110461 | 0.00166011 | 2.90E-11 | 4.24E-05 | 19.2590668 |
| 111 | rs2192527 | G | A | 0.465502 | 0.0141172 | 0.00154567 | 6.60E-20 | 9.13E-05 | 41.51441819 |
| 112 | rs2192649 | G | T | 0.500777 | 0.00852919 | 0.00155109 | 3.80E-08 | 3.33E-05 | 15.11900009 |
| 113 | rs2243928 | G | C | 0.648983 | -0.0120426 | 0.00163641 | 1.90E-13 | 5.43E-05 | 24.67569538 |
| 114 | rs2274224 | C | G | 0.435459 | -0.0159065 | 0.00155477 | 1.40E-24 | 0.000113195 | 51.46796092 |
| 115 | rs2276936 | C | A | 0.470368 | -0.0123522 | 0.00154228 | 1.20E-15 | 7.03E-05 | 31.96191394 |
| 116 | rs2291127 | T | C | 0.156234 | -0.0137926 | 0.00211992 | 7.70E-11 | 2.45E-05 | 11.16065435 |
| 117 | rs2307111 | C | T | 0.39509 | -0.0171461 | 0.00157693 | 1.50E-27 | 0.000124297 | 56.51643322 |
| 118 | rs2481899 | G | A | 0.551171 | 0.00930927 | 0.00156135 | 2.50E-09 | 3.87E-05 | 17.58906986 |
| 119 | rs2499468 | A | C | 0.650982 | 0.0100294 | 0.00161777 | 5.70E-10 | 3.84E-05 | 17.46537539 |
| 120 | rs2508782 | G | A | 0.569716 | 0.00937803 | 0.00155792 | 1.70E-09 | 3.91E-05 | 17.76607945 |
| 121 | rs252749 | A | G | 0.245982 | -0.0102553 | 0.00178627 | 9.40E-09 | 2.69E-05 | 12.22720904 |
| 122 | rs256904 | T | A | 0.746327 | -0.0111835 | 0.00176815 | 2.50E-10 | 3.33E-05 | 15.14827663 |
| 123 | rs262953 | A | G | 0.625978 | -0.0102573 | 0.00159162 | 1.20E-10 | 4.28E-05 | 19.44868408 |
| 124 | rs2660241 | C | T | 0.36485 | 0.0108857 | 0.00160421 | 1.20E-11 | 4.69E-05 | 21.34171957 |
| 125 | rs2678204 | G | T | 0.340185 | 0.0136291 | 0.00162387 | 4.70E-17 | 6.96E-05 | 31.62477743 |
| 126 | rs2692741 | C | G | 0.367194 | 0.00909731 | 0.0016107 | 1.60E-08 | 3.26E-05 | 14.82535434 |
| 127 | rs2785988 | A | C | 0.297382 | 0.0210728 | 0.00168127 | 4.90E-36 | 0.000144402 | 65.65900776 |
| 128 | rs2802774 | A | C | 0.547302 | 0.0105954 | 0.00156929 | 1.50E-11 | 4.97E-05 | 22.58986181 |
| 129 | rs2814993 | A | G | 0.13976 | 0.0259158 | 0.00221742 | 1.50E-31 | 7.22E-05 | 32.84696935 |
| 130 | rs2855818 | A | G | 0.241541 | 0.0153102 | 0.00181772 | 3.70E-17 | 5.72E-05 | 25.99463011 |
| 131 | rs28651380 | G | A | 0.281193 | 0.0104262 | 0.00171657 | 1.20E-09 | 3.28E-05 | 14.9137961 |
| 132 | rs28714450 | T | C | 0.675249 | -0.0101175 | 0.00165029 | 8.70E-10 | 3.63E-05 | 16.48481847 |
| 133 | rs28742003 | T | C | 0.205276 | -0.021134 | 0.00191016 | 1.90E-28 | 8.79E-05 | 39.94336588 |
| 134 | rs2888778 | G | T | 0.652153 | 0.00998353 | 0.00161504 | 6.30E-10 | 3.81E-05 | 17.33740498 |
| 135 | rs2943653 | T | C | 0.672855 | -0.0155134 | 0.00163892 | 2.90E-21 | 8.68E-05 | 39.44809261 |
| 136 | rs2954033 | G | A | 0.695224 | 0.0124143 | 0.00167398 | 1.20E-13 | 5.13E-05 | 23.30772113 |
| 137 | rs2957678 | C | T | 0.498199 | 0.0116378 | 0.00154184 | 4.40E-14 | 6.27E-05 | 28.48740129 |
| 138 | rs2960420 | G | C | 0.350731 | -0.00912091 | 0.00162066 | 1.80E-08 | 3.17E-05 | 14.42557724 |
| 139 | rs2966859 | G | A | 0.789007 | -0.0111483 | 0.00189749 | 4.20E-09 | 2.53E-05 | 11.49334042 |
| 140 | rs2984618 | T | G | 0.416215 | 0.0117369 | 0.0015617 | 5.70E-14 | 6.04E-05 | 27.44963372 |
| 141 | rs3113509 | T | C | 0.731962 | -0.0107277 | 0.00174038 | 7.10E-10 | 3.28E-05 | 14.90913006 |
| 142 | rs319775 | C | T | 0.60881 | 0.00883995 | 0.00158047 | 2.20E-08 | 3.28E-05 | 14.90180739 |
| 143 | rs33836 | T | C | 0.464327 | -0.0163804 | 0.00156772 | 1.50E-25 | 0.000119455 | 54.31448546 |
| 144 | rs34338229 | A | G | 0.332008 | 0.00897307 | 0.00164195 | 4.60E-08 | 2.91E-05 | 13.2471607 |
| 145 | rs34483452 | A | C | 0.13631 | 0.0245244 | 0.00226433 | 2.50E-27 | 6.08E-05 | 27.62214628 |
| 146 | rs34656389 | G | A | 0.367474 | 0.0103136 | 0.00159872 | 1.10E-10 | 4.26E-05 | 19.34760967 |
| 147 | rs347551 | G | C | 0.472318 | 0.00988966 | 0.00156805 | 2.80E-10 | 4.36E-05 | 19.82881277 |
| 148 | rs3743861 | C | G | 0.415256 | -0.0103504 | 0.00156479 | 3.70E-11 | 4.67E-05 | 21.24869275 |
| 149 | rs3754963 | T | A | 0.255074 | -0.0114802 | 0.00176393 | 7.60E-11 | 3.54E-05 | 16.09752109 |
| 150 | rs3764002 | T | C | 0.261517 | -0.0186517 | 0.00175335 | 2.00E-26 | 9.61E-05 | 43.71292584 |
| 151 | rs3765971 | T | C | 0.65847 | -0.0102079 | 0.00162025 | 3.00E-10 | 3.93E-05 | 17.85330993 |
| 152 | rs3766823 | A | G | 0.172004 | 0.0140143 | 0.00203896 | 6.30E-12 | 2.96E-05 | 13.45653153 |
| 153 | rs3791709 | T | A | 0.282417 | 0.0103311 | 0.00171169 | 1.60E-09 | 3.25E-05 | 14.76549703 |
| 154 | rs3803286 | G | A | 0.666725 | -0.0115932 | 0.00163624 | 1.40E-12 | 4.91E-05 | 22.31061387 |
| 155 | rs3817428 | G | C | 0.265007 | -0.0152755 | 0.00174905 | 2.50E-18 | 6.54E-05 | 29.71552712 |
| 156 | rs3826408 | T | C | 0.456883 | 0.00915425 | 0.00154807 | 3.40E-09 | 3.82E-05 | 17.35431223 |
| 157 | rs3923501 | T | C | 0.475818 | 0.0102177 | 0.00154181 | 3.40E-11 | 4.82E-05 | 21.90869767 |
| 158 | rs394608 | C | T | 0.537747 | 0.0123902 | 0.00155475 | 1.60E-15 | 6.94E-05 | 31.57559073 |
| 159 | rs396354 | C | T | 0.715662 | -0.00986214 | 0.00170937 | 8.00E-09 | 2.98E-05 | 13.54734091 |
| 160 | rs396755 | G | C | 0.571071 | -0.0102893 | 0.00155919 | 4.10E-11 | 4.69E-05 | 21.33524136 |
| 161 | rs40071 | C | T | 0.179498 | -0.014887 | 0.00201352 | 1.40E-13 | 3.54E-05 | 16.10219961 |
| 162 | rs41307479 | G | C | 0.220577 | 0.0124051 | 0.00185992 | 2.60E-11 | 3.36E-05 | 15.2963619 |
| 163 | rs41310284 | A | C | 0.101004 | -0.0201214 | 0.00257596 | 5.70E-15 | 2.44E-05 | 11.0808522 |
| 164 | rs429343 | G | A | 0.576575 | -0.0111228 | 0.00155872 | 9.60E-13 | 5.47E-05 | 24.86429679 |
| 165 | rs429358 | C | T | 0.154044 | -0.0215029 | 0.00213952 | 9.20E-24 | 5.79E-05 | 26.32735113 |
| 166 | rs4320040 | G | T | 0.560267 | -0.0101838 | 0.00154798 | 4.70E-11 | 4.69E-05 | 21.32659844 |
| 167 | rs4398538 | C | T | 0.642513 | -0.010008 | 0.00161101 | 5.20E-10 | 3.90E-05 | 17.72904385 |
| 168 | rs441792 | G | A | 0.486601 | 0.0119298 | 0.00154217 | 1.00E-14 | 6.58E-05 | 29.90105336 |
| 169 | rs4466418 | A | G | 0.562278 | 0.00997781 | 0.00155638 | 1.40E-10 | 4.45E-05 | 20.23187134 |
| 170 | rs4496901 | T | G | 0.65522 | -0.00954008 | 0.00162185 | 4.00E-09 | 3.44E-05 | 15.63345229 |
| 171 | rs4500770 | T | A | 0.362615 | -0.00977478 | 0.00160607 | 1.20E-09 | 3.77E-05 | 17.12289088 |
| 172 | rs4547574 | T | A | 0.227697 | -0.0105978 | 0.00183563 | 7.80E-09 | 2.58E-05 | 11.7231807 |
| 173 | rs4690324 | A | G | 0.823217 | -0.0126754 | 0.00202574 | 3.90E-10 | 2.51E-05 | 11.39592915 |
| 174 | rs4709745 | C | T | 0.307185 | 0.00934269 | 0.00167115 | 2.30E-08 | 2.93E-05 | 13.3036586 |
| 175 | rs4718964 | T | G | 0.413126 | 0.0111105 | 0.00157055 | 1.50E-12 | 5.34E-05 | 24.2684667 |
| 176 | rs4759318 | T | C | 0.362201 | 0.0104428 | 0.00160707 | 8.10E-11 | 4.29E-05 | 19.50942787 |
| 177 | rs4762951 | G | A | 0.780444 | -0.0113662 | 0.00186311 | 1.10E-09 | 2.81E-05 | 12.7550134 |
| 178 | rs4776337 | A | G | 0.468075 | 0.00998636 | 0.00154808 | 1.10E-10 | 4.56E-05 | 20.7224626 |
| 179 | rs479018 | A | G | 0.332499 | -0.0144775 | 0.00166392 | 3.30E-18 | 7.39E-05 | 33.60661068 |
| 180 | rs4820323 | G | C | 0.580958 | -0.0169383 | 0.00156868 | 3.50E-27 | 0.000124865 | 56.77478906 |
| 181 | rs482787 | C | T | 0.325766 | 0.0092676 | 0.00164764 | 1.90E-08 | 3.06E-05 | 13.89849177 |
| 182 | rs4837119 | A | T | 0.517271 | -0.0101722 | 0.00154703 | 4.90E-11 | 4.75E-05 | 21.59248079 |
| 183 | rs4876611 | G | A | 0.720188 | 0.0173998 | 0.00171871 | 4.30E-24 | 9.09E-05 | 41.31074885 |
| 184 | rs4894808 | C | G | 0.40013 | -0.0100803 | 0.00160438 | 3.30E-10 | 4.17E-05 | 18.95120725 |
| 185 | rs4908676 | G | A | 0.457718 | 0.00966112 | 0.00154844 | 4.40E-10 | 4.25E-05 | 19.32573038 |
| 186 | rs4959613 | A | C | 0.594024 | 0.00955622 | 0.00161789 | 3.50E-09 | 3.70E-05 | 16.82762438 |
| 187 | rs529200 | G | A | 0.527782 | 0.0106966 | 0.00154338 | 4.20E-12 | 5.27E-05 | 23.94384235 |
| 188 | rs55810445 | T | C | 0.151196 | -0.0140163 | 0.00215528 | 7.90E-11 | 2.39E-05 | 10.85539286 |
| 189 | rs56094641 | G | A | 0.404646 | 0.041033 | 0.00157131 | 2.50E-150 | 0.000722707 | 328.8027078 |
| 190 | rs56218501 | T | C | 0.211814 | -0.0128916 | 0.00188796 | 8.60E-12 | 3.42E-05 | 15.56877354 |
| 191 | rs56328878 | A | C | 0.267194 | -0.00975461 | 0.00173683 | 2.00E-08 | 2.72E-05 | 12.35266888 |
| 192 | rs56369689 | G | A | 0.348594 | -0.00952112 | 0.00165613 | 9.00E-09 | 3.30E-05 | 15.01071279 |
| 193 | rs56399737 | T | C | 0.44917 | -0.0118589 | 0.00155668 | 2.60E-14 | 6.32E-05 | 28.71930783 |
| 194 | rs57636386 | C | T | 0.083836 | -0.0252015 | 0.00279375 | 1.90E-19 | 2.75E-05 | 12.50033058 |
| 195 | rs58862095 | T | C | 0.419262 | -0.0131217 | 0.00156604 | 5.30E-17 | 7.52E-05 | 34.19014938 |
| 196 | rs59227842 | G | A | 0.311468 | 0.0170384 | 0.00167907 | 3.40E-24 | 9.71E-05 | 44.17004643 |
| 197 | rs59499656 | T | A | 0.343189 | -0.0137829 | 0.00163035 | 2.80E-17 | 7.09E-05 | 32.22197499 |
| 198 | rs6064113 | C | T | 0.765465 | -0.0105166 | 0.00185641 | 1.50E-08 | 2.53E-05 | 11.52325059 |
| 199 | rs61903695 | G | A | 0.254879 | 0.0118139 | 0.00177153 | 2.60E-11 | 3.72E-05 | 16.89252763 |
| 200 | rs61910767 | T | C | 0.164197 | -0.0142941 | 0.00208312 | 6.80E-12 | 2.84E-05 | 12.92393096 |
| 201 | rs61969510 | C | T | 0.279026 | 0.0127422 | 0.00173502 | 2.10E-13 | 4.77E-05 | 21.70166006 |
| 202 | rs61975147 | C | T | 0.167185 | -0.0142041 | 0.00207301 | 7.30E-12 | 2.88E-05 | 13.07407797 |
| 203 | rs62107261 | C | T | 0.048327 | -0.0479135 | 0.00359643 | 1.70E-40 | 3.59E-05 | 16.3265279 |
| 204 | rs62190394 | T | C | 0.317033 | 0.0151644 | 0.00165383 | 4.80E-20 | 8.01E-05 | 36.41126673 |
| 205 | rs62218301 | G | A | 0.166049 | -0.0127956 | 0.00208683 | 8.70E-10 | 2.29E-05 | 10.41266743 |
| 206 | rs62443626 | A | G | 0.463946 | -0.0106141 | 0.00155316 | 8.30E-12 | 5.11E-05 | 23.23058825 |
| 207 | rs6480350 | C | T | 0.575106 | -0.00953851 | 0.00156462 | 1.10E-09 | 4.00E-05 | 18.16424108 |
| 208 | rs6491427 | G | A | 0.289013 | -0.013993 | 0.00170162 | 2.00E-16 | 6.11E-05 | 27.79266401 |
| 209 | rs6500594 | G | T | 0.246636 | 0.0110602 | 0.00179199 | 6.70E-10 | 3.11E-05 | 14.15656009 |
| 210 | rs6561937 | A | T | 0.753684 | -0.0117153 | 0.00179629 | 6.90E-11 | 3.47E-05 | 15.79352293 |
| 211 | rs657685 | C | G | 0.456088 | -0.00989059 | 0.00154908 | 1.70E-10 | 4.45E-05 | 20.22653067 |
| 212 | rs6602997 | T | C | 0.711856 | 0.0226029 | 0.00170655 | 4.80E-40 | 0.000158293 | 71.97619099 |
| 213 | rs6688826 | C | T | 0.298206 | 0.0105922 | 0.0016789 | 2.80E-10 | 3.66E-05 | 16.6606797 |
| 214 | rs6693294 | G | A | 0.688566 | -0.0136932 | 0.0016614 | 1.70E-16 | 6.41E-05 | 29.13593808 |
| 215 | rs6699744 | T | A | 0.616045 | 0.0142768 | 0.00158851 | 2.50E-19 | 8.41E-05 | 38.21546947 |
| 216 | rs6750646 | T | C | 0.201154 | -0.0117765 | 0.00192871 | 1.00E-09 | 2.64E-05 | 11.98201551 |
| 217 | rs6752378 | A | C | 0.486256 | 0.0224027 | 0.00153822 | 4.80E-48 | 0.000233101 | 105.9997611 |
| 218 | rs6754292 | T | C | 0.644629 | -0.00932639 | 0.00161898 | 8.40E-09 | 3.34E-05 | 15.20472642 |
| 219 | rs67609008 | C | T | 0.283593 | 0.0113458 | 0.00171702 | 3.90E-11 | 3.90E-05 | 17.74270783 |
| 220 | rs6782581 | G | C | 0.439266 | -0.0101996 | 0.00155143 | 4.90E-11 | 4.68E-05 | 21.29290002 |
| 221 | rs6840236 | C | T | 0.464881 | 0.0121284 | 0.00154596 | 4.30E-15 | 6.74E-05 | 30.62386658 |
| 222 | rs6843910 | A | T | 0.45449 | -0.0104169 | 0.00155088 | 1.90E-11 | 4.92E-05 | 22.37160296 |
| 223 | rs6847975 | A | G | 0.35617 | 0.0113515 | 0.00161543 | 2.10E-12 | 4.98E-05 | 22.64688544 |
| 224 | rs685149 | G | A | 0.645411 | -0.00954107 | 0.00161396 | 3.40E-09 | 3.52E-05 | 15.99608184 |
| 225 | rs6875585 | C | A | 0.670422 | 0.011204 | 0.00164464 | 9.60E-12 | 4.51E-05 | 20.50966898 |
| 226 | rs6927268 | G | T | 0.206332 | -0.0138777 | 0.00190462 | 3.20E-13 | 3.82E-05 | 17.38875397 |
| 227 | rs6948959 | A | G | 0.744356 | -0.0105935 | 0.00177956 | 2.60E-09 | 2.97E-05 | 13.48686273 |
| 228 | rs6973656 | G | A | 0.397021 | 0.00928324 | 0.00157441 | 3.70E-09 | 3.66E-05 | 16.64650749 |
| 229 | rs6977416 | A | G | 0.334244 | -0.0118159 | 0.00164828 | 7.60E-13 | 5.03E-05 | 22.87179649 |
| 230 | rs7020 | A | G | 0.437471 | 0.0130676 | 0.00155587 | 4.50E-17 | 7.64E-05 | 34.72166067 |
| 231 | rs7027304 | T | C | 0.652657 | 0.011578 | 0.0016271 | 1.10E-12 | 5.05E-05 | 22.95790039 |
| 232 | rs704061 | C | T | 0.455038 | 0.0136927 | 0.00154796 | 9.10E-19 | 8.54E-05 | 38.80947439 |
| 233 | rs7046679 | G | C | 0.678548 | -0.00914252 | 0.00165304 | 3.20E-08 | 2.94E-05 | 13.34451064 |
| 234 | rs7124681 | A | C | 0.408419 | 0.0222563 | 0.0015648 | 6.60E-46 | 0.000215019 | 97.77546503 |
| 235 | rs7132908 | A | G | 0.38447 | 0.0197321 | 0.00158551 | 1.50E-35 | 0.000161246 | 73.31932294 |
| 236 | rs7133378 | A | G | 0.319439 | 0.0185779 | 0.00165616 | 3.30E-29 | 0.000120341 | 54.71721776 |
| 237 | rs71658797 | A | T | 0.120747 | 0.0236276 | 0.00236585 | 1.70E-23 | 4.66E-05 | 21.17889671 |
| 238 | rs719802 | C | T | 0.6144 | -0.0115343 | 0.0015826 | 3.10E-13 | 5.54E-05 | 25.169844 |
| 239 | rs7206608 | G | C | 0.321575 | 0.0101676 | 0.00165236 | 7.60E-10 | 3.63E-05 | 16.52173104 |
| 240 | rs7216121 | G | A | 0.626339 | -0.0109061 | 0.00161091 | 1.30E-11 | 4.72E-05 | 21.45518198 |
| 241 | rs7218014 | C | T | 0.197302 | 0.0211077 | 0.00194282 | 1.70E-27 | 8.22E-05 | 37.39074902 |
| 242 | rs72634814 | A | G | 0.345475 | -0.0128866 | 0.00169042 | 2.50E-14 | 5.78E-05 | 26.28351888 |
| 243 | rs72697297 | C | T | 0.179917 | -0.0153167 | 0.00203008 | 4.50E-14 | 3.69E-05 | 16.7987996 |
| 244 | rs72703757 | C | G | 0.198824 | 0.0115499 | 0.00194851 | 3.10E-09 | 2.46E-05 | 11.19403567 |
| 245 | rs72892910 | T | G | 0.172194 | 0.0221404 | 0.00204337 | 2.30E-27 | 7.36E-05 | 33.47214491 |
| 246 | rs72917533 | C | T | 0.185499 | -0.0138669 | 0.0019837 | 2.70E-12 | 3.25E-05 | 14.76665629 |
| 247 | rs72976986 | A | G | 0.190192 | -0.0171142 | 0.00198547 | 6.70E-18 | 5.03E-05 | 22.88815597 |
| 248 | rs7321331 | A | G | 0.742296 | 0.0107397 | 0.00177343 | 1.40E-09 | 3.09E-05 | 14.03123721 |
| 249 | rs73213501 | C | A | 0.171839 | -0.0152527 | 0.00204286 | 8.20E-14 | 3.49E-05 | 15.86704307 |
| 250 | rs7357754 | G | A | 0.500129 | 0.0116159 | 0.00154667 | 5.90E-14 | 6.20E-05 | 28.20365618 |
| 251 | rs7442885 | G | C | 0.214136 | -0.0128176 | 0.00188108 | 9.50E-12 | 3.44E-05 | 15.62712155 |
| 252 | rs74618095 | C | T | 0.157976 | 0.0132675 | 0.00213178 | 4.90E-10 | 2.27E-05 | 10.3049749 |
| 253 | rs7463186 | G | A | 0.516044 | 0.0088055 | 0.00154188 | 1.10E-08 | 3.58E-05 | 16.29082772 |
| 254 | rs7535438 | A | C | 0.290883 | -0.00950812 | 0.00169742 | 2.10E-08 | 2.85E-05 | 12.9445622 |
| 255 | rs7598246 | C | T | 0.583694 | 0.0085494 | 0.00156226 | 4.40E-08 | 3.20E-05 | 14.55474279 |
| 256 | rs7680610 | G | A | 0.652245 | 0.0089709 | 0.00163023 | 3.70E-08 | 3.02E-05 | 13.7372442 |
| 257 | rs7704382 | G | C | 0.433417 | 0.0101719 | 0.00155683 | 6.40E-11 | 4.61E-05 | 20.96714872 |
| 258 | rs7707394 | A | G | 0.357305 | -0.0126609 | 0.00160546 | 3.10E-15 | 6.28E-05 | 28.56469321 |
| 259 | rs7762794 | G | A | 0.285523 | 0.00930584 | 0.00170461 | 4.80E-08 | 2.67E-05 | 12.15990412 |
| 260 | rs7773916 | T | C | 0.213136 | -0.0109353 | 0.00188165 | 6.20E-09 | 2.49E-05 | 11.32867476 |
| 261 | rs7789056 | A | G | 0.563037 | -0.0127186 | 0.00155559 | 2.90E-16 | 7.24E-05 | 32.89496587 |
| 262 | rs78296744 | A | G | 0.272344 | -0.0132887 | 0.00173423 | 1.80E-14 | 5.12E-05 | 23.2726505 |
| 263 | rs7843109 | T | C | 0.688667 | 0.0103657 | 0.00166877 | 5.20E-10 | 3.64E-05 | 16.5456039 |
| 264 | rs78744936 | A | G | 0.265987 | 0.0113474 | 0.00175799 | 1.10E-10 | 3.58E-05 | 16.2692569 |
| 265 | rs7893571 | T | G | 0.665915 | 0.0100326 | 0.00163856 | 9.20E-10 | 3.67E-05 | 16.68100033 |
| 266 | rs7925725 | C | A | 0.411245 | 0.0111592 | 0.00156858 | 1.10E-12 | 5.39E-05 | 24.50978564 |
| 267 | rs7942368 | T | C | 0.217538 | -0.0105396 | 0.00189331 | 2.60E-08 | 2.32E-05 | 10.54973012 |
| 268 | rs7960609 | G | A | 0.326919 | 0.00918558 | 0.00164856 | 2.50E-08 | 3.01E-05 | 13.66319548 |
| 269 | rs7966251 | A | G | 0.255023 | -0.00981406 | 0.00177103 | 3.00E-08 | 2.57E-05 | 11.668285 |
| 270 | rs7972728 | A | C | 0.73765 | 0.0167619 | 0.00175434 | 1.20E-21 | 7.77E-05 | 35.33557912 |
| 271 | rs7975187 | G | A | 0.213862 | 0.0115294 | 0.00188095 | 8.80E-10 | 2.78E-05 | 12.63372672 |
| 272 | rs798549 | A | C | 0.730794 | -0.00992339 | 0.00174424 | 1.30E-08 | 2.80E-05 | 12.73584003 |
| 273 | rs7987928 | A | G | 0.799871 | -0.0138119 | 0.00193112 | 8.50E-13 | 3.60E-05 | 16.37804402 |
| 274 | rs8096564 | T | G | 0.298122 | 0.0107485 | 0.00169686 | 2.40E-10 | 3.69E-05 | 16.79204073 |
| 275 | rs811054 | T | C | 0.537276 | 0.0095458 | 0.00155694 | 8.70E-10 | 4.11E-05 | 18.691593 |
| 276 | rs812949 | C | T | 0.729218 | 0.0133557 | 0.00173358 | 1.30E-14 | 5.16E-05 | 23.44084069 |
| 277 | rs815163 | C | T | 0.563216 | -0.00909072 | 0.00154941 | 4.40E-09 | 3.73E-05 | 16.93752009 |
| 278 | rs853961 | T | G | 0.505909 | 0.0122048 | 0.00154046 | 2.30E-15 | 6.90E-05 | 31.38324842 |
| 279 | rs879620 | T | C | 0.613285 | 0.014719 | 0.00158851 | 1.90E-20 | 8.96E-05 | 40.72837238 |
| 280 | rs881929 | T | G | 0.375396 | -0.0147342 | 0.00159151 | 2.10E-20 | 8.84E-05 | 40.19714996 |
| 281 | rs885114 | A | G | 0.275581 | -0.0146358 | 0.00172806 | 2.50E-17 | 6.30E-05 | 28.64242911 |
| 282 | rs9289630 | C | G | 0.38912 | 0.0123827 | 0.00158474 | 5.60E-15 | 6.38E-05 | 29.02752019 |
| 283 | rs9304665 | A | T | 0.763642 | 0.0118765 | 0.00182093 | 6.90E-11 | 3.38E-05 | 15.35653774 |
| 284 | rs9321191 | C | T | 0.1994 | -0.0108699 | 0.0019277 | 1.70E-08 | 2.23E-05 | 10.15197934 |
| 285 | rs9372414 | T | C | 0.347629 | -0.00933072 | 0.00162144 | 8.70E-09 | 3.30E-05 | 15.02040451 |
| 286 | rs9568867 | A | G | 0.129188 | 0.0193443 | 0.00231898 | 7.30E-17 | 3.44E-05 | 15.65675128 |
| 287 | rs957919 | T | C | 0.278023 | 0.0138914 | 0.00171911 | 6.40E-16 | 5.77E-05 | 26.21452028 |
| 288 | rs9645335 | T | C | 0.367818 | -0.00955748 | 0.00159404 | 2.00E-09 | 3.68E-05 | 16.7188933 |
| 289 | rs972283 | G | A | 0.511944 | -0.0143133 | 0.00154037 | 1.50E-20 | 9.49E-05 | 43.15099973 |
| 290 | rs9788550 | C | G | 0.24751 | -0.0158356 | 0.00179488 | 1.10E-18 | 6.38E-05 | 28.99662663 |
| 291 | rs9814758 | G | T | 0.355786 | -0.0136044 | 0.00161607 | 3.80E-17 | 7.15E-05 | 32.48752155 |
| 292 | rs9843653 | C | T | 0.51154 | 0.0167794 | 0.00154077 | 1.30E-27 | 0.000130363 | 59.27485986 |
| 293 | rs9865173 | A | T | 0.703199 | -0.0120222 | 0.00168776 | 1.10E-12 | 4.66E-05 | 21.18060883 |
| 294 | rs9892466 | A | T | 0.333275 | 0.0100634 | 0.00163774 | 8.00E-10 | 3.69E-05 | 16.7800461 |

^a^ A1 = Effect_allele.exposure; A2 = Other_allele.exposure; Eaf = Eaf.exposure; Beta = Beta.exposure; SE = Se.exposure; P = Pval.exposure.

^b^ The SNPs associated with smoking or obesity were excluded. ^c^ The SNPs associated with telomere length or obesity were excluded. ^d^ The SNPs associated with telomere length or smoking were excluded.

**Supplemental Table 2 Total R^2^, F-statistic and test power for each estimate in the study**

| **Exposures** | **Total R^2^** | **Total F-statistic** | **Test power** |
| --- | --- | --- | --- |
| Telomere length ^a^ | 0.0068 | 38.0199 | 0.45 |
| Heavy smoking | 0.0027 | 48.6456 | 0.05 |
| Smoking initiation | 0.0012 | 19.1902 | 0.05 |
| Age of initiation ^b^ | 0.0006 | 12.2338 | 0.15 |
| Smoking cessation | 0.0005 | 15.2184 | 0.05 |
| Maternal smoking ^b^ | 0.0012 | 12.2799 | 1.00 |
| Heavy drinking | 0.0007 | 23.6185 | 0.08 |
| Body mass index ^c^ | 0.0158 | 31.5935 | 0.62 |
| Body fat percentage ^c^ | 0.0158 | 24.8766 | 0.96 |

^a^ The SNPs associated with smoking or obesity were excluded.

^b^ The SNPs associated with telomere length or obesity were excluded.

^c^ The SNPs associated with telomere length or smoking were excluded.

**Supplemental Table 3 Results of MR-PRESSO in the study**

| **Exposures** | **Causal estimate (beta)** | **Standard deviation** | **P value for estimate ^a^** | **P value for global test ^b^** |
| --- | --- | --- | --- | --- |
| Telomere length ^c^ | -0.7452 | 0.1695 | 3.4016e-05 | 0.6577 |
| Heavy smoking | 0.0643 | 0.1024 | 0.5376 | 0.9017 |
| Smoking initiation | 0.1069 | 0.1888 | 0.5730 | 0.1593 |
| Age of initiation ^d^ | -2.2272 | 0.6715 | 0.0051 | 0.3313 |
| Smoking cessation | -0.1599 | 0.2128 | 0.4628 | 0.8323 |
| Maternal smoking ^d^ | 2.5789 | 1.0102 | 0.0149 | 0.4510 |
| Heavy drinking | 0.3686 | 0.4310 | 0.3997 | 0.5023 |
| Body mass index ^e^ | 0.3540 | 0.1257 | 0.0051 | 0.2003 |
| Body fat percentage ^e^ | 0.5317 | 0.1772 | 0.0029 | 0.1527 |

^a^ P value for estimate indicated P value for causal relationship between the exposures and outcome.

^b^ P value for global test indicated P value for outlier assessment and horizontal pleiotropy.

^c^ The SNPs associated with smoking or obesity were excluded.

^d^ The SNPs associated with telomere length or obesity were excluded.

^e^ The SNPs associated with telomere length or smoking were excluded.
