# Supplementary material for: Genetic association of telomere length, obesity and tobacoo smoking with idiopathic pulmonary fibrosis risk
Source: BMC Public Health. 2023 May 11;23:868. doi: 10.1186/s12889-023-15733-5 (PMC10176771; doi:10.1186/s12889-023-15733-5)
Supplement: Supplementary file 2 — Supplementary Material 2 [file 12889_2023_15733_MOESM2_ESM.docx]

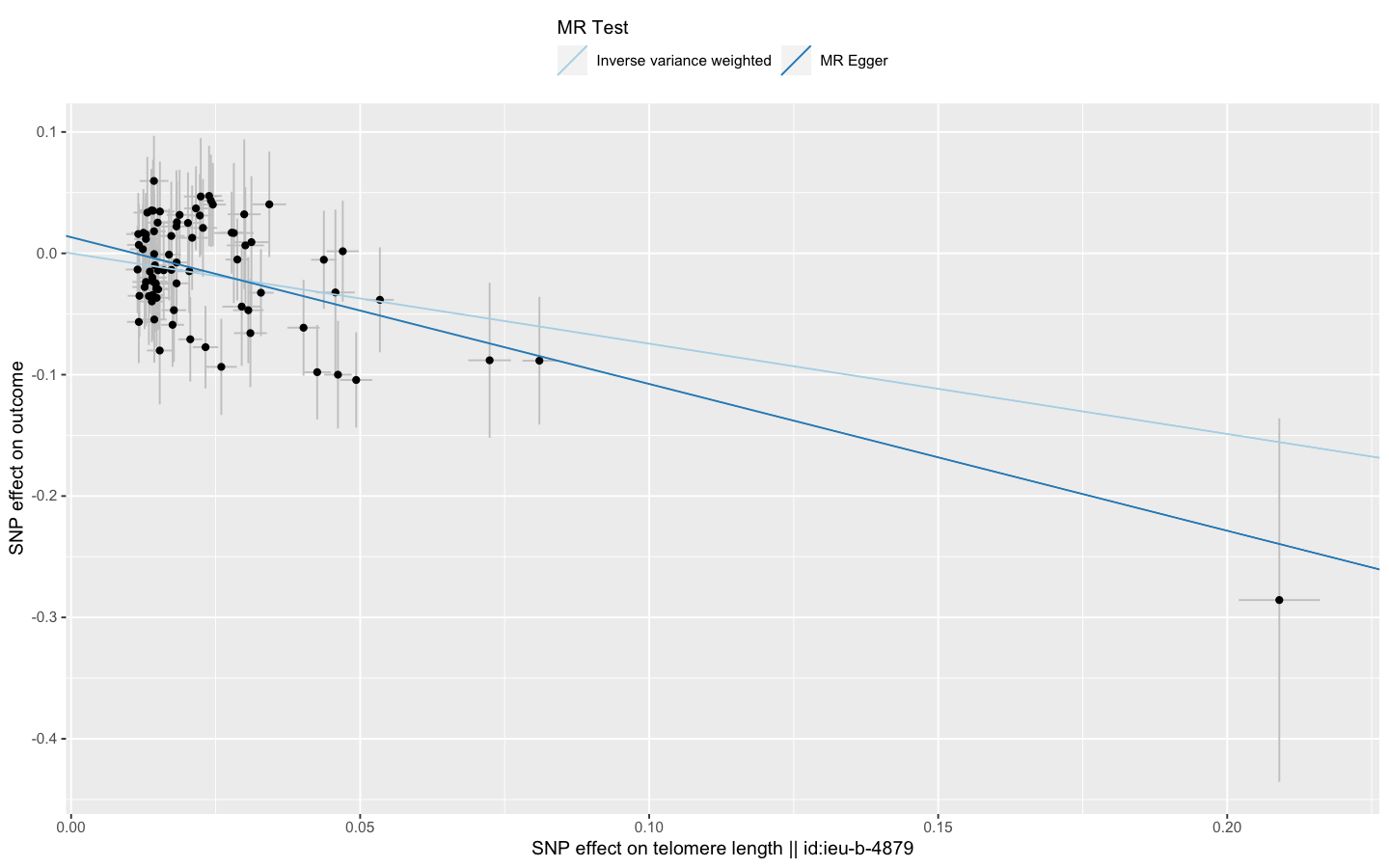


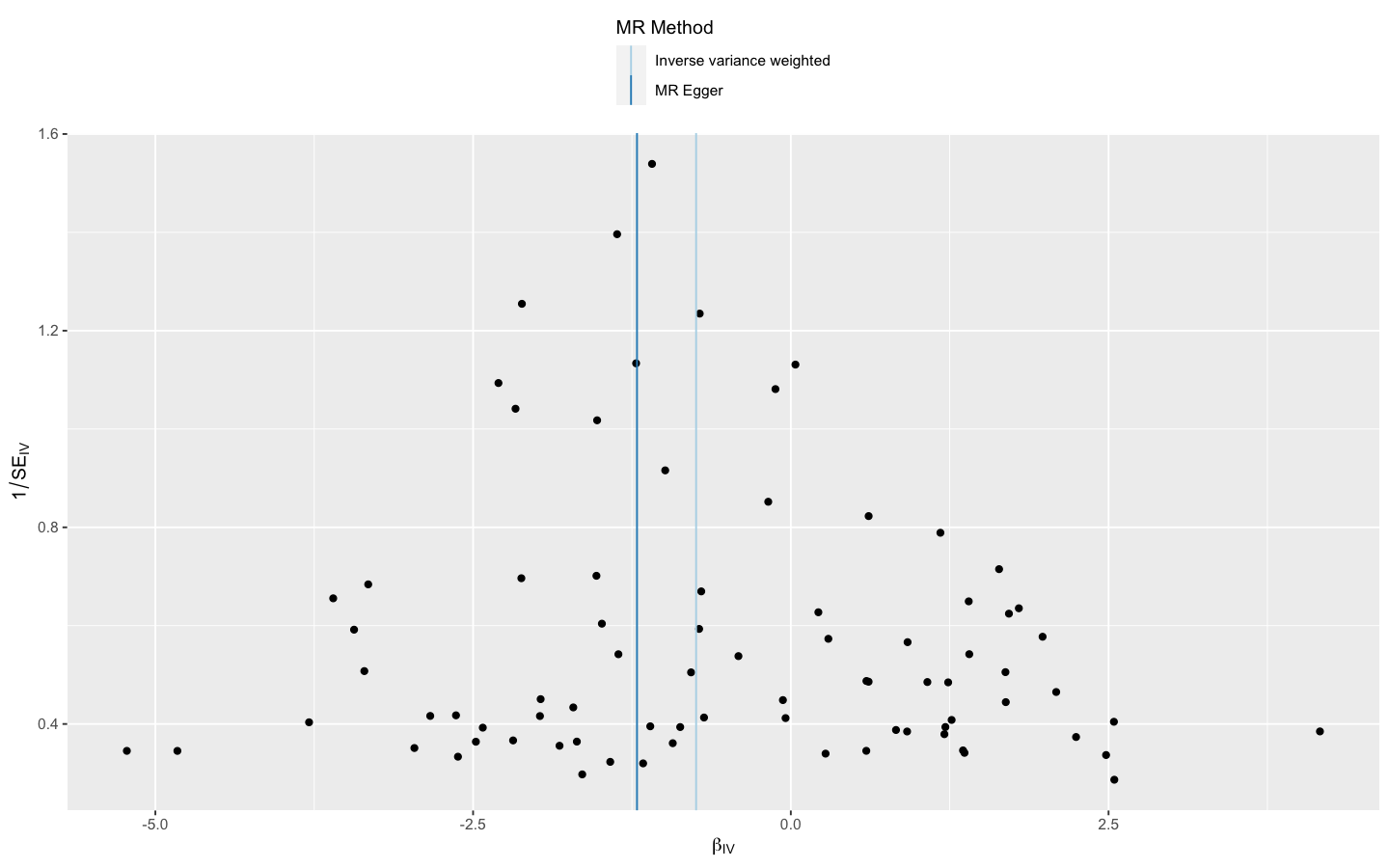


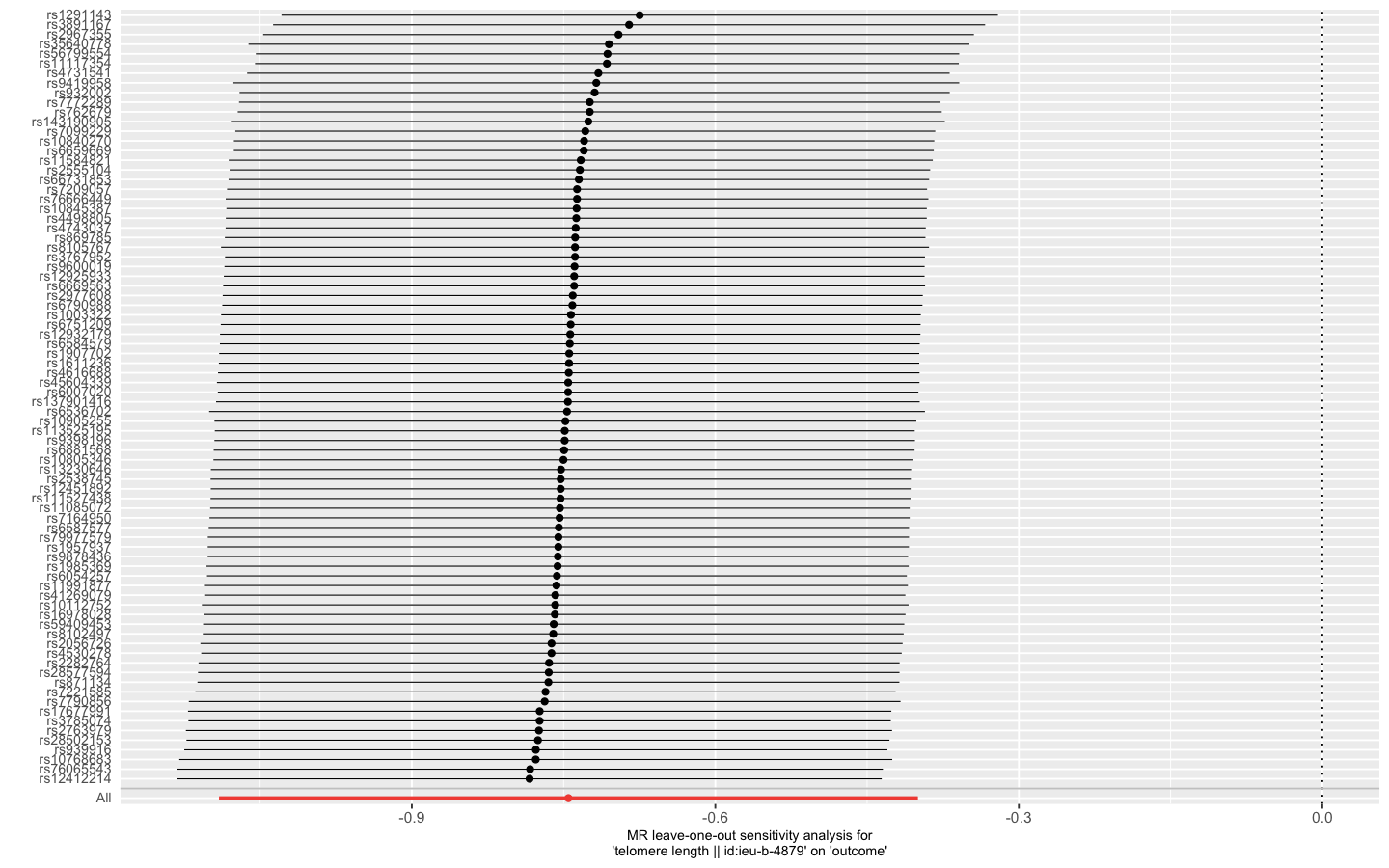


**Supplemental figure 1 Scatter, leave-one-out and funnel plots for effect of telomere length on idiopathic pulmonary fibrosis risk (excluding the SNPs associated with smoking or obesity)**


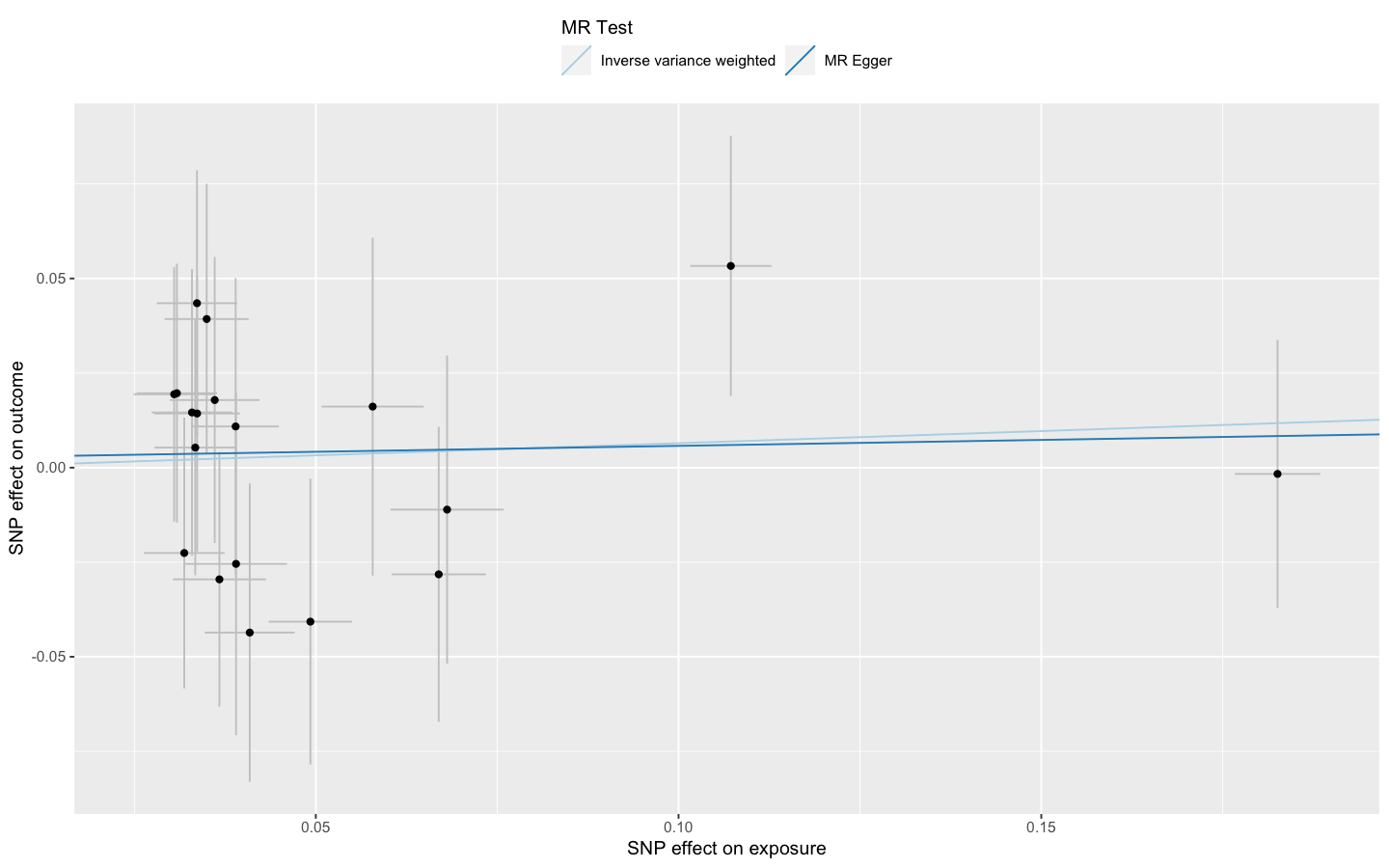

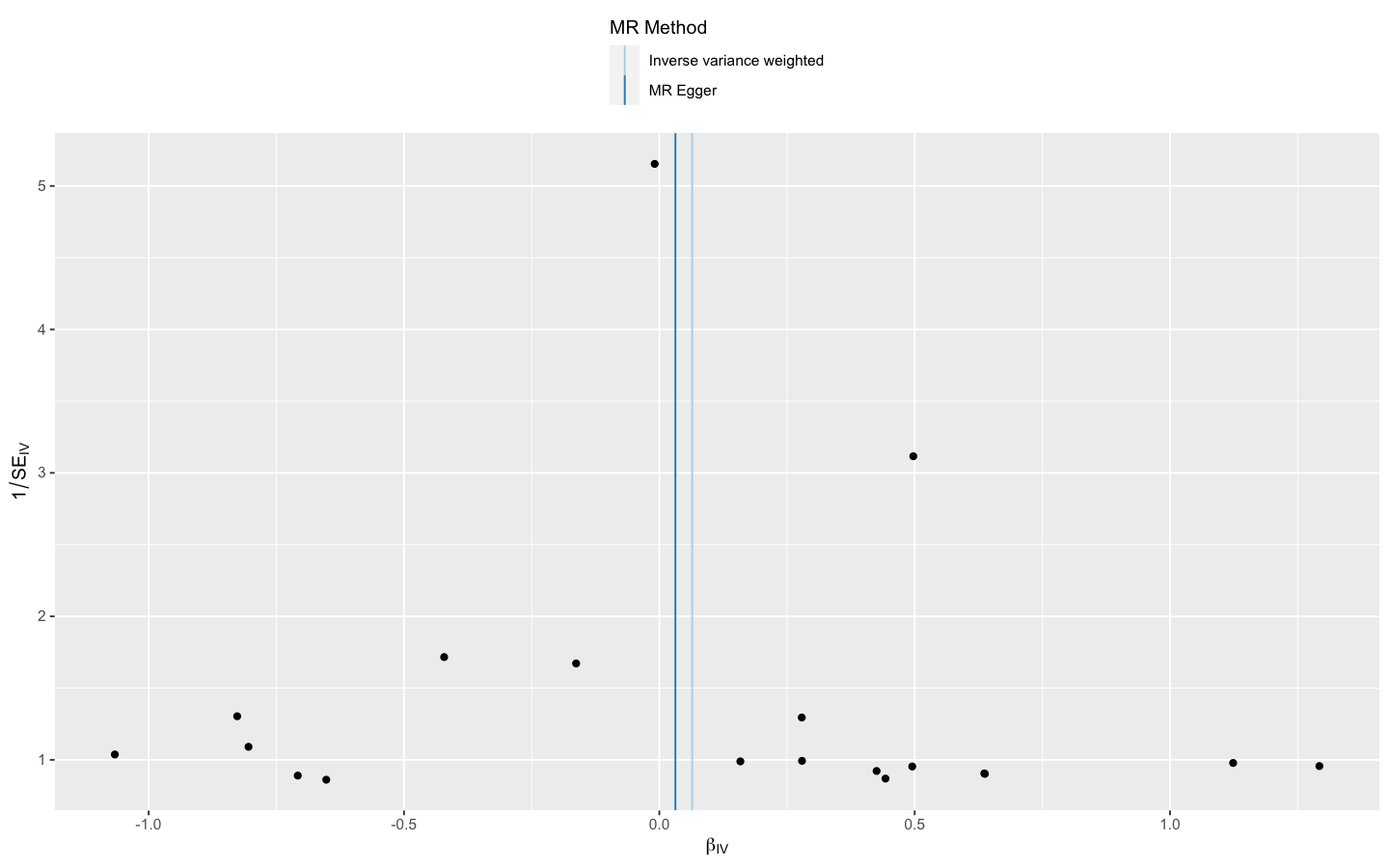

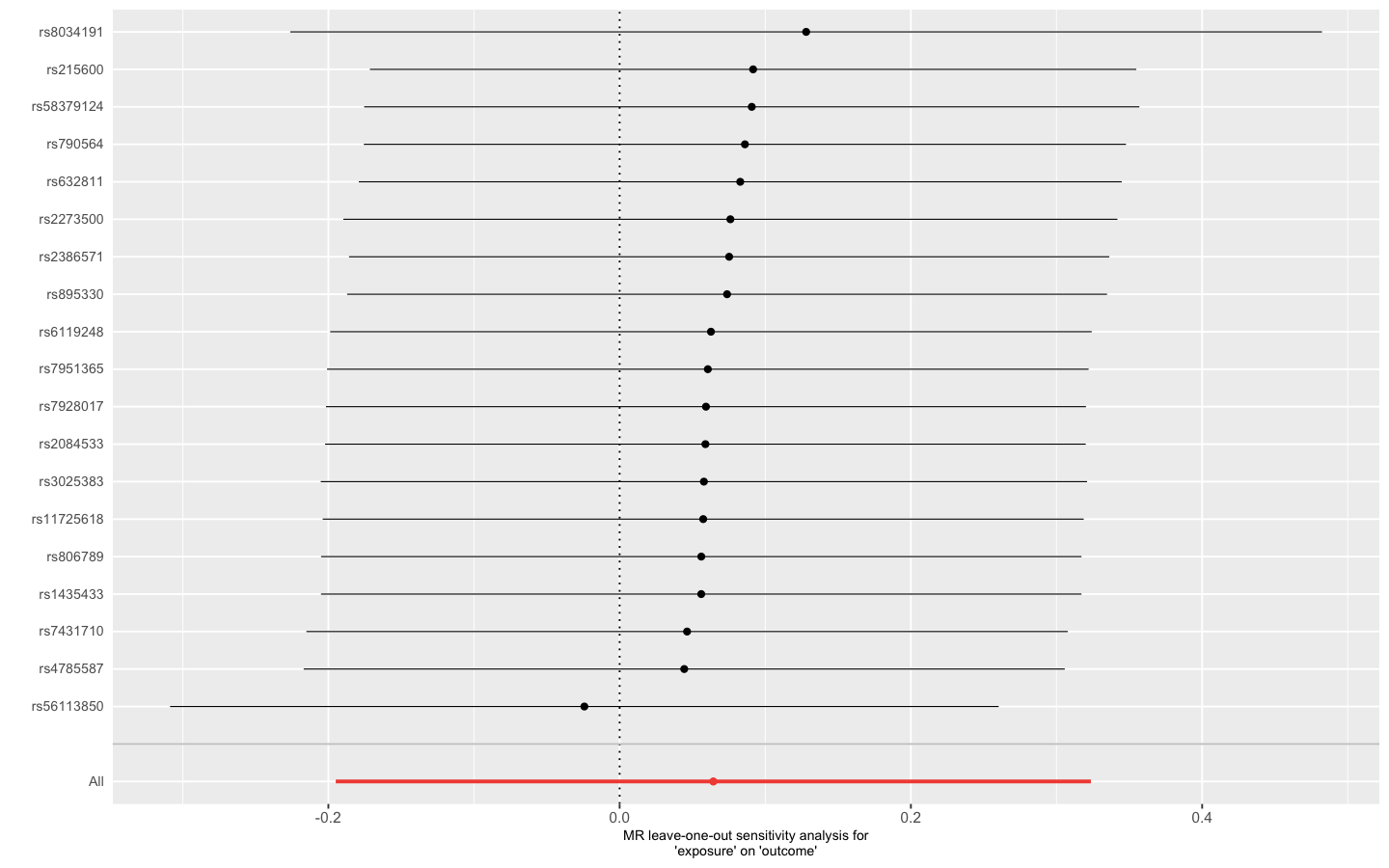


**Supplemental figure 2 Scatter, leave-one-out and funnel plots for effect of heavy smoking on idiopathic pulmonary fibrosis risk**


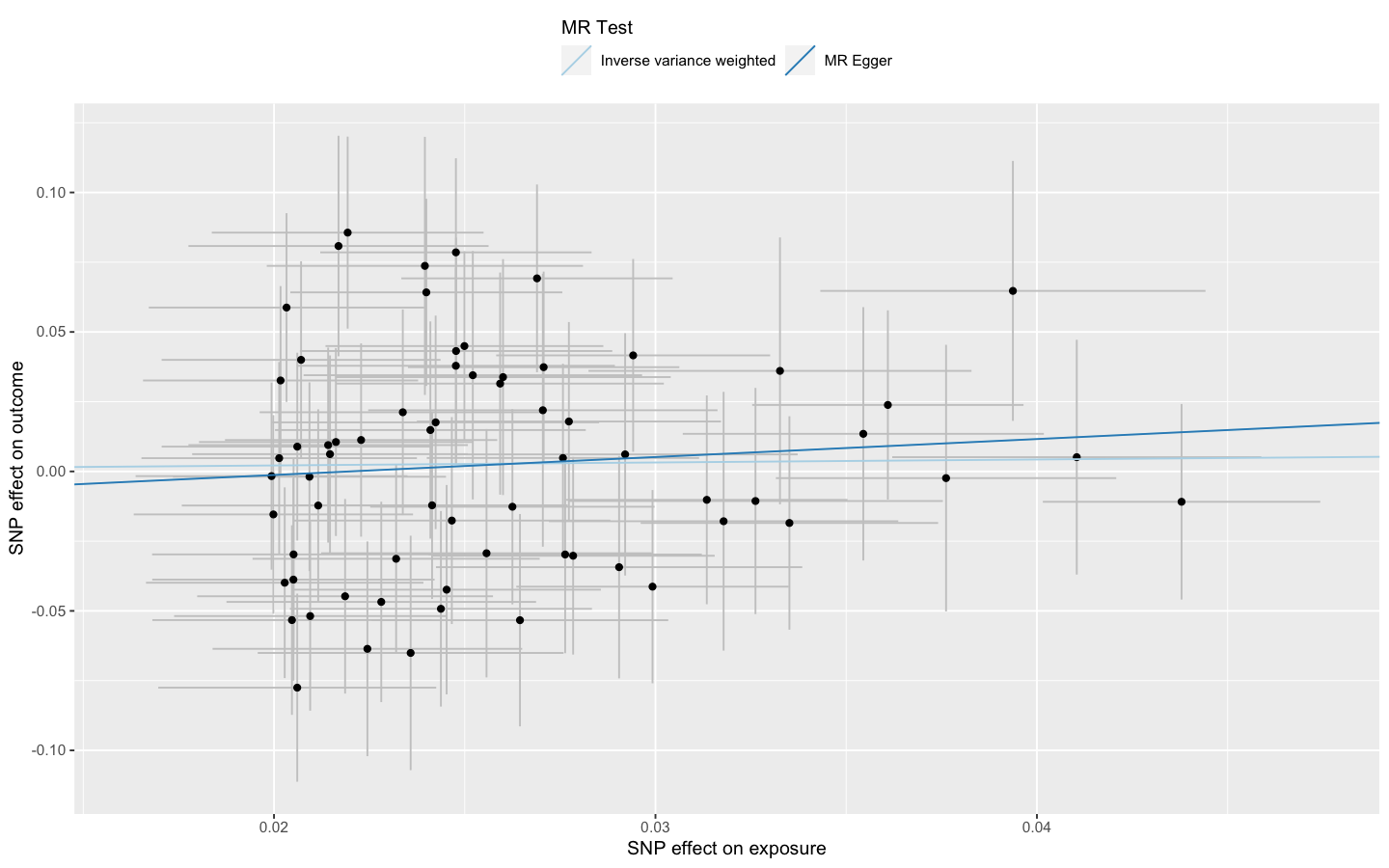


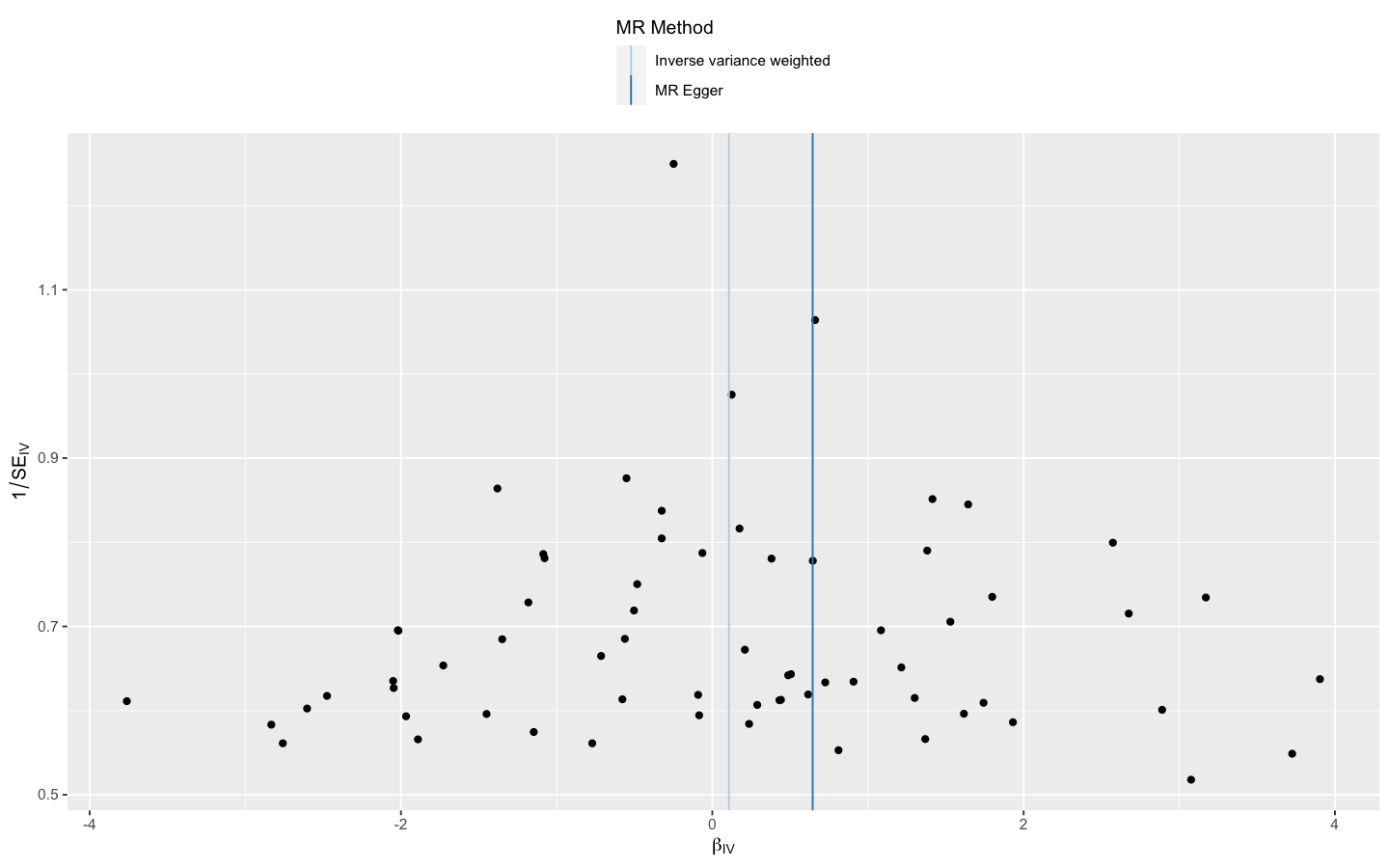


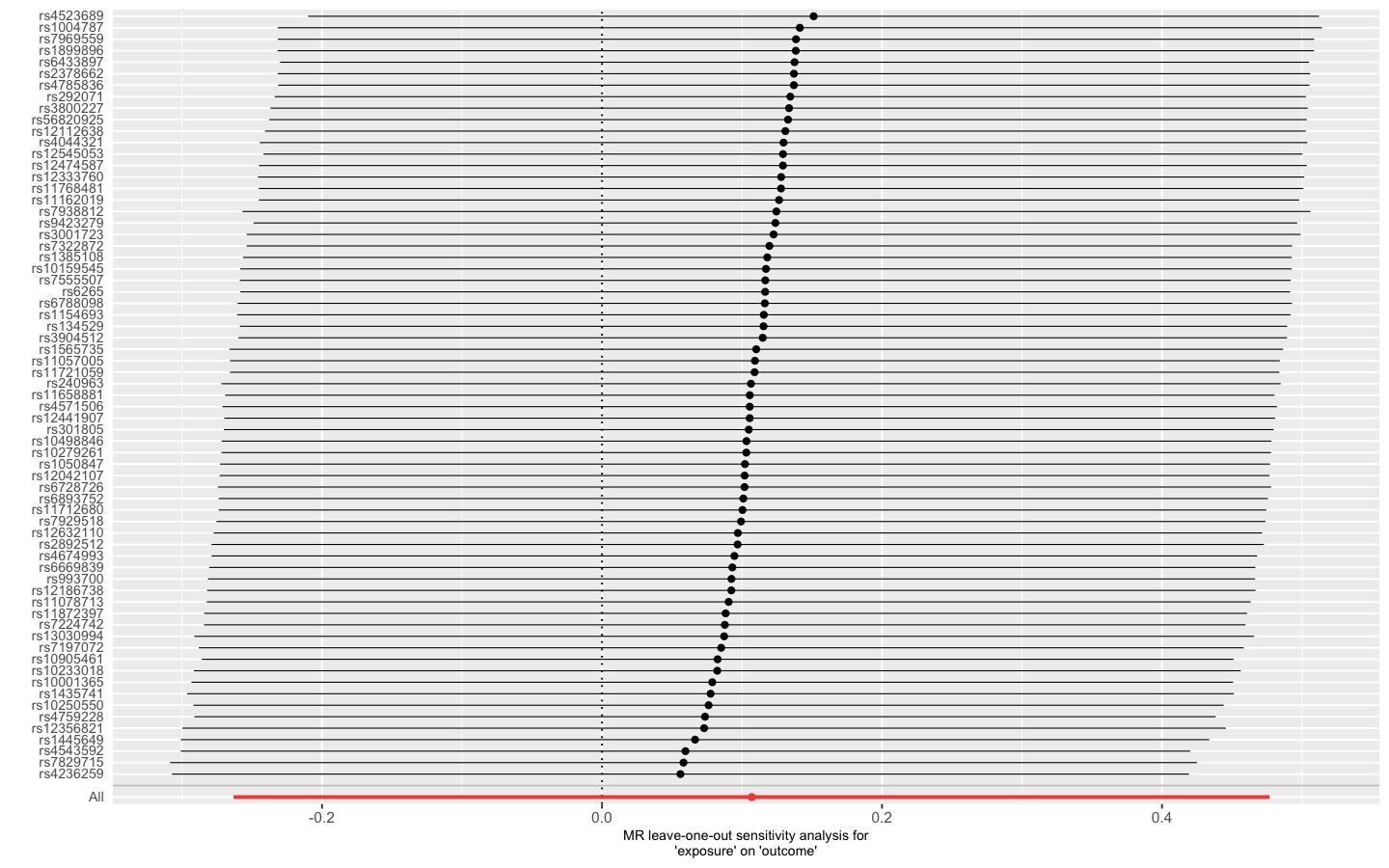


**Supplemental figure 3 Scatter, leave-one-out and funnel plots for effect of smoking**

**initiation on idiopathic pulmonary fibrosis risk**


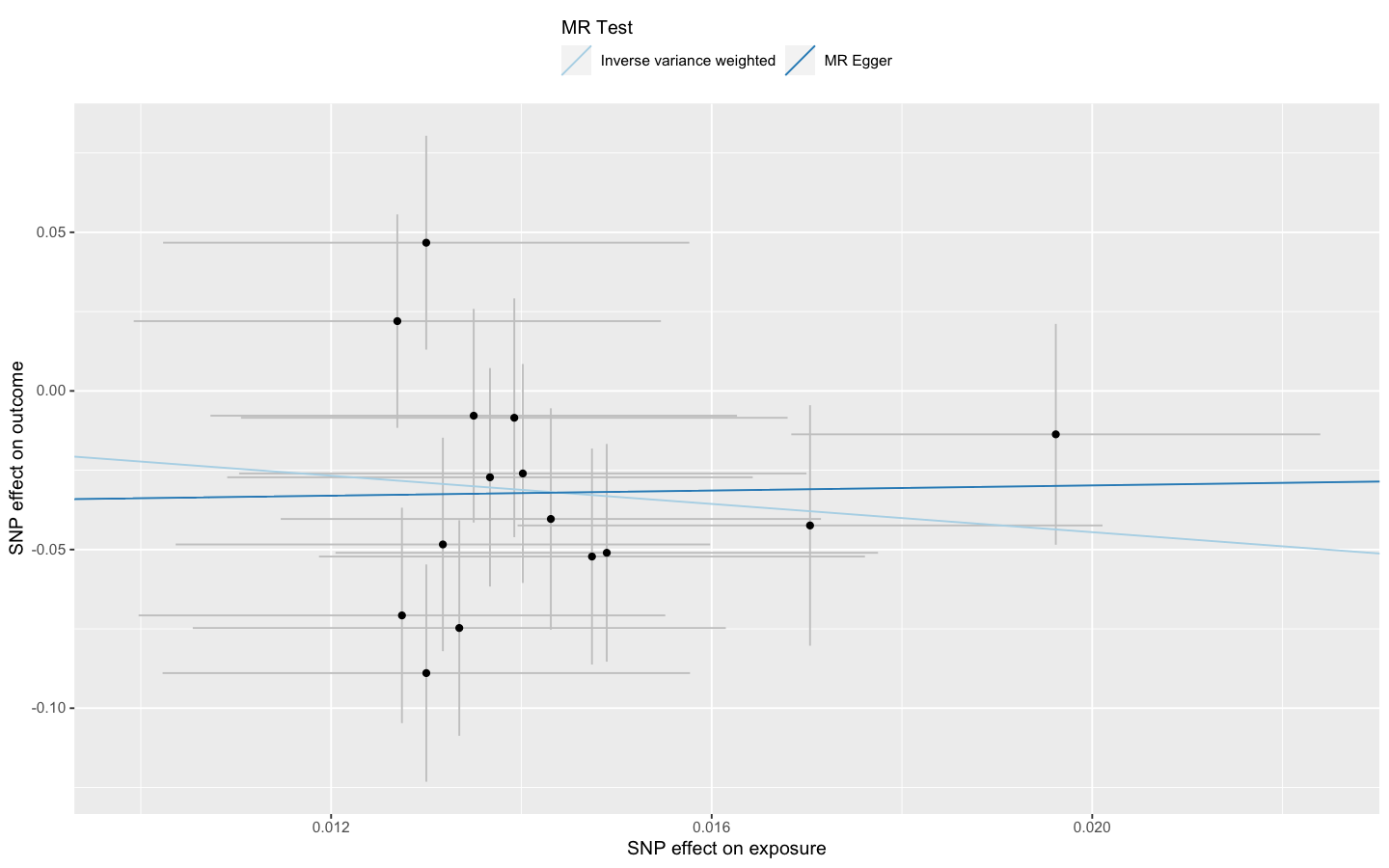


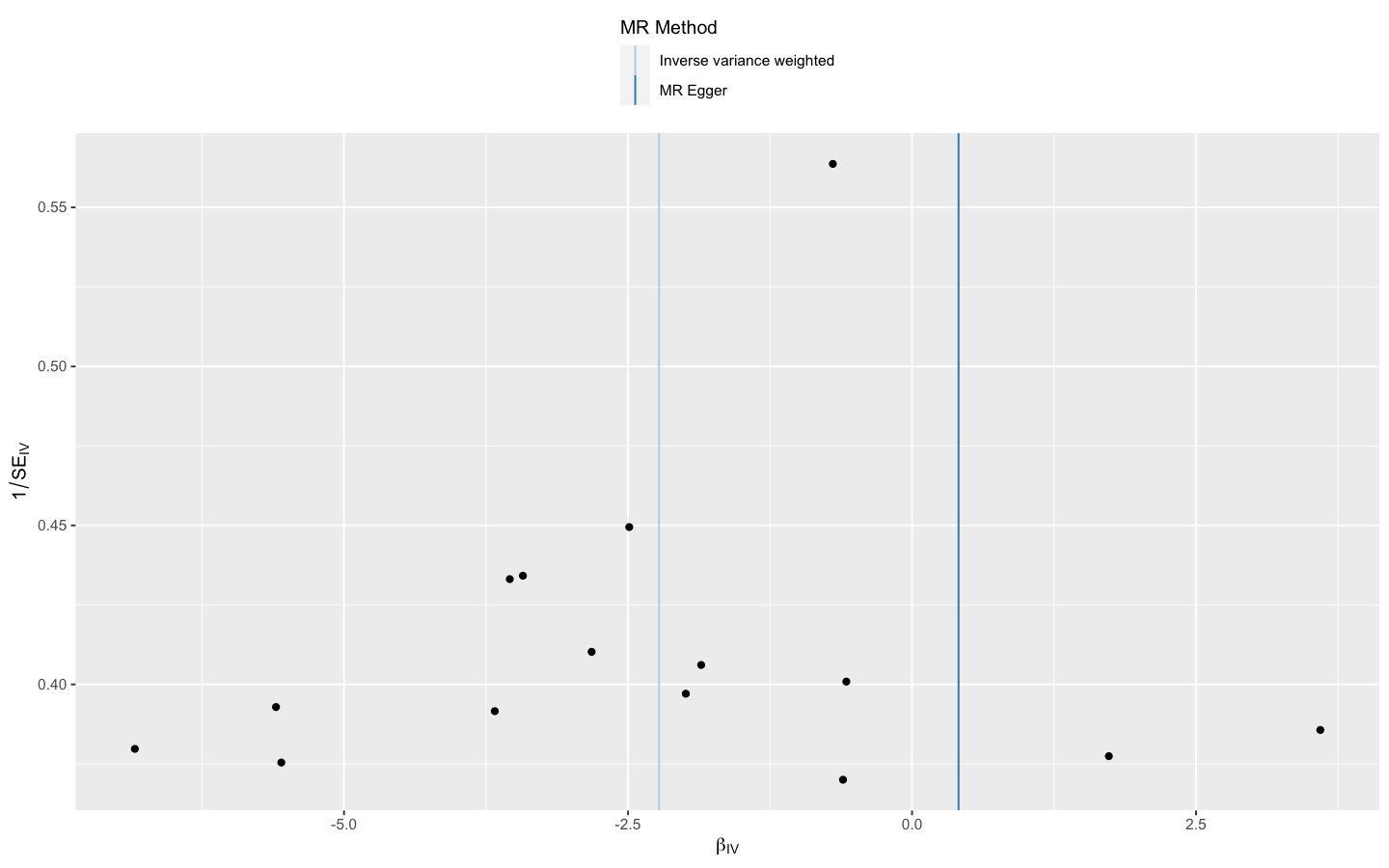


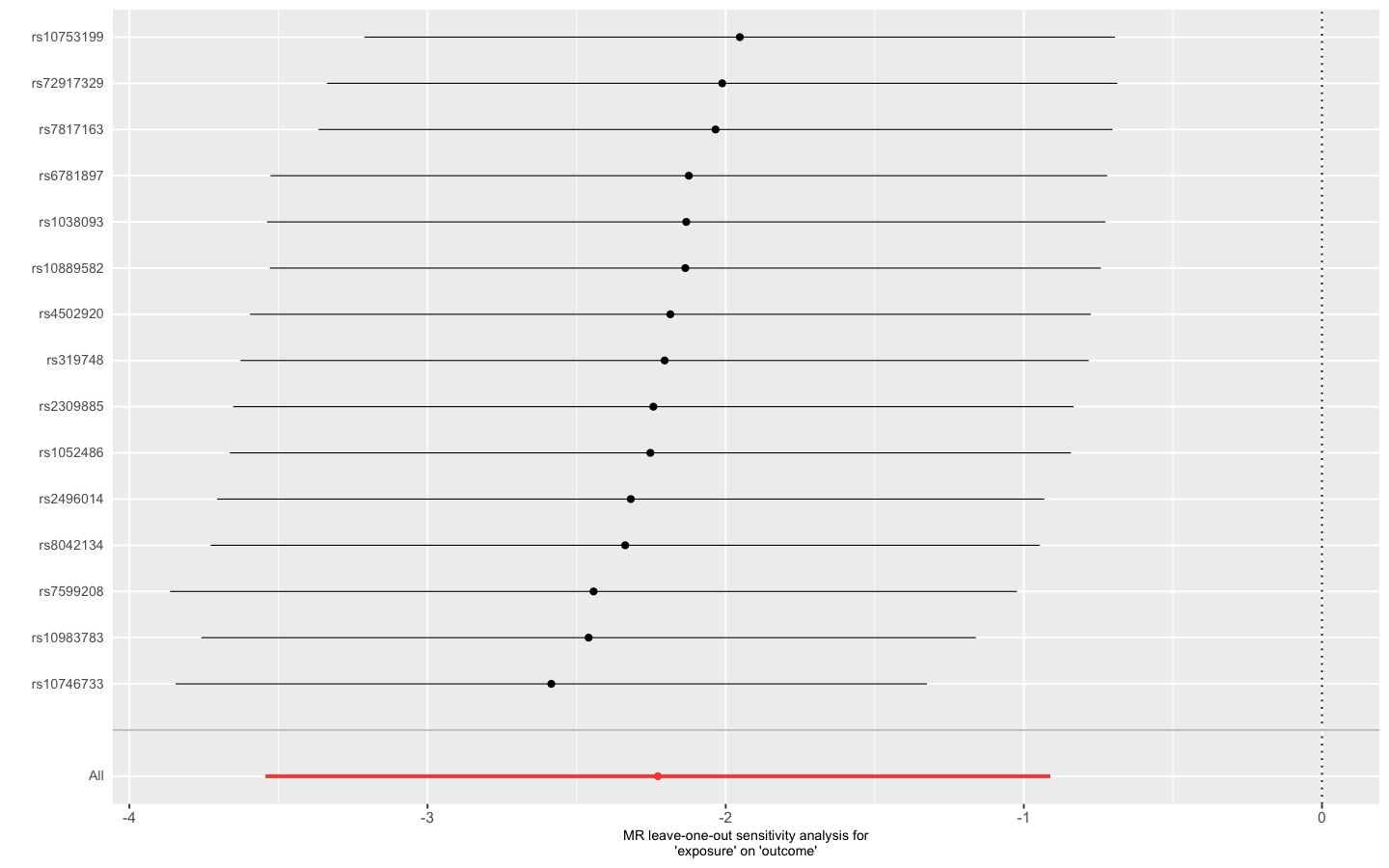


**Supplemental figure 4 Scatter, leave-one-out and funnel plots for effect of age of smoking initiation on idiopathic pulmonary fibrosis risk (excluding the SNPs associated with telomere length or obesity)**


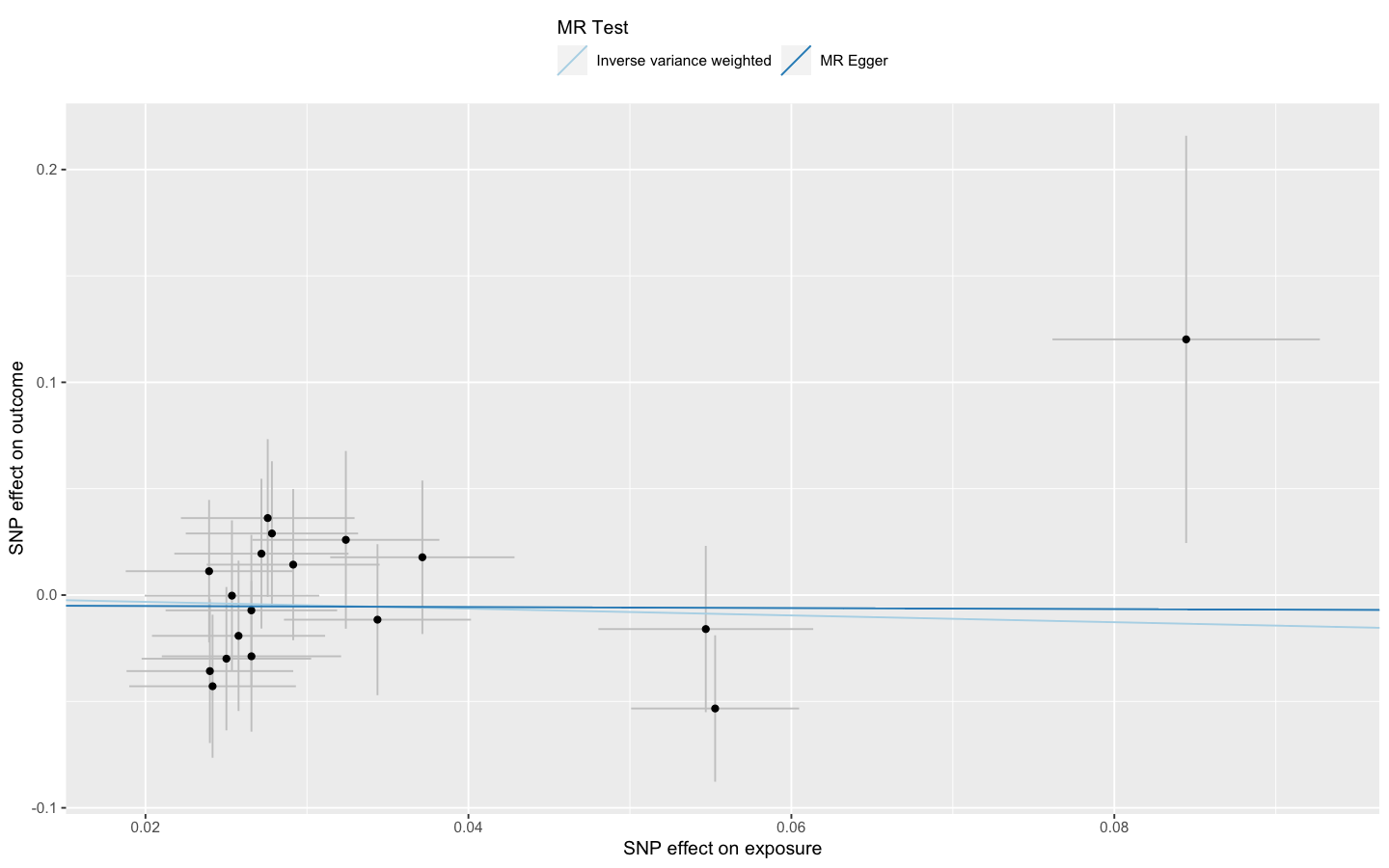


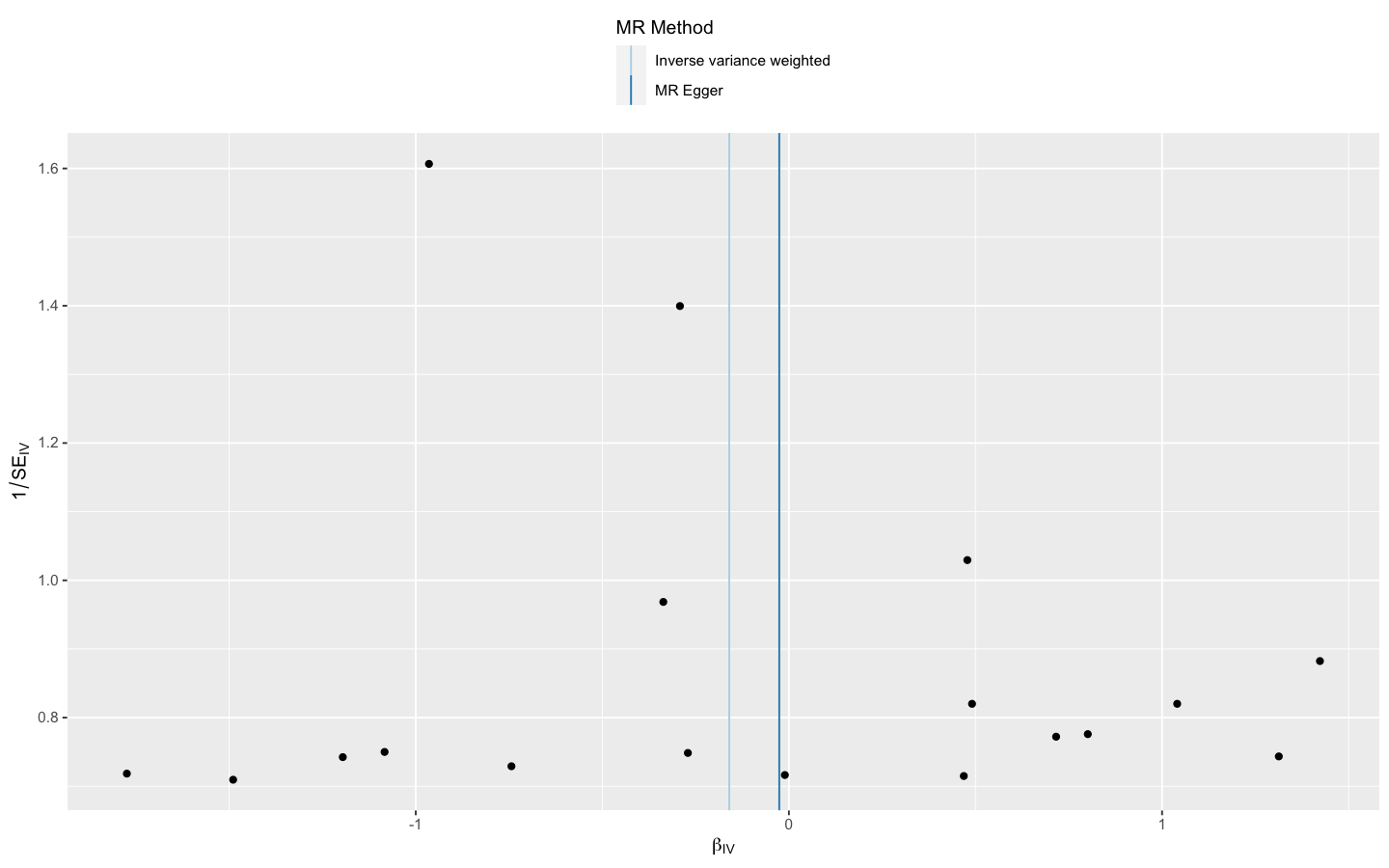


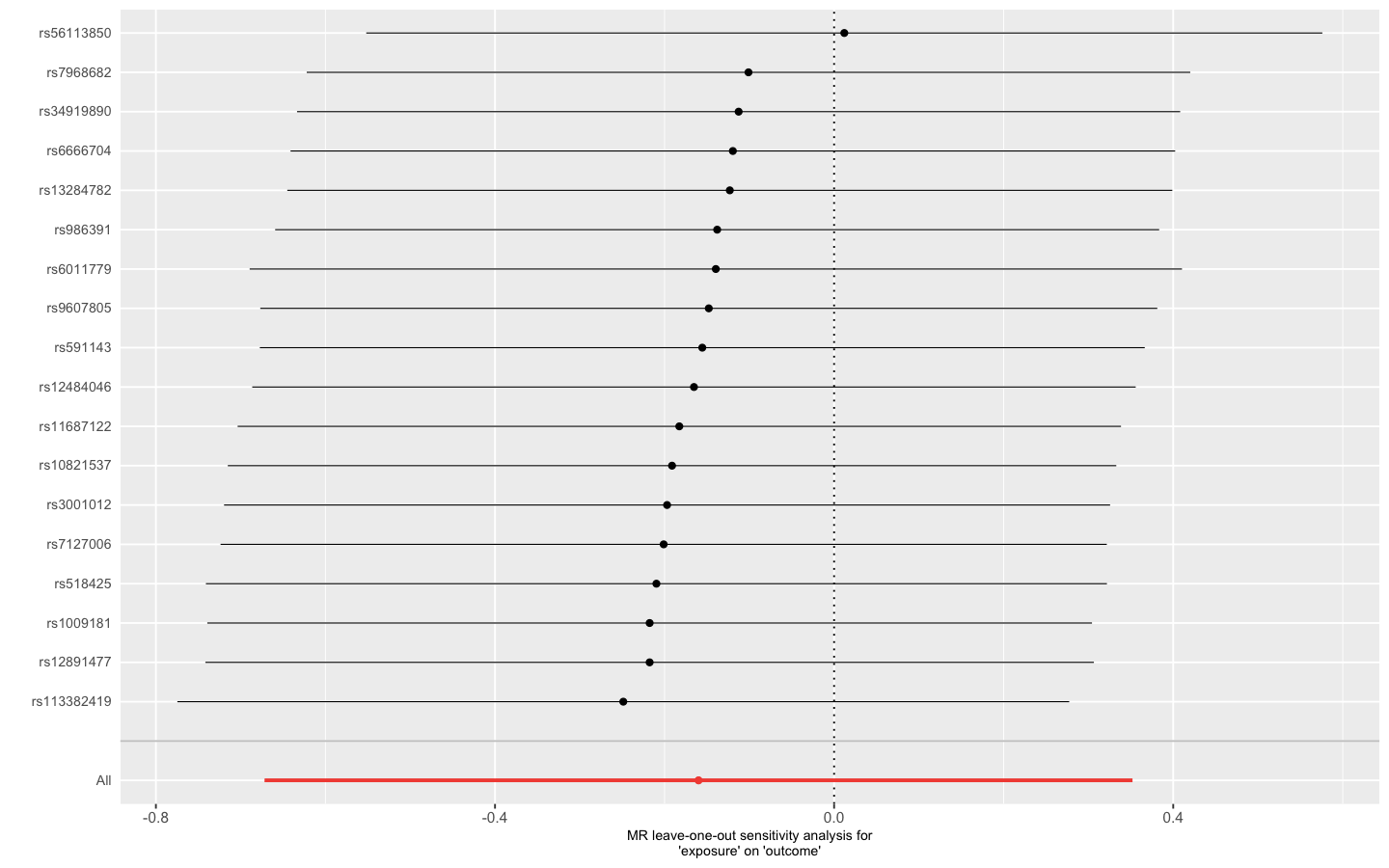


**Supplemental figure 5 Scatter, leave-one-out and funnel plots for effect of smoking cessation on idiopathic pulmonary fibrosis risk**


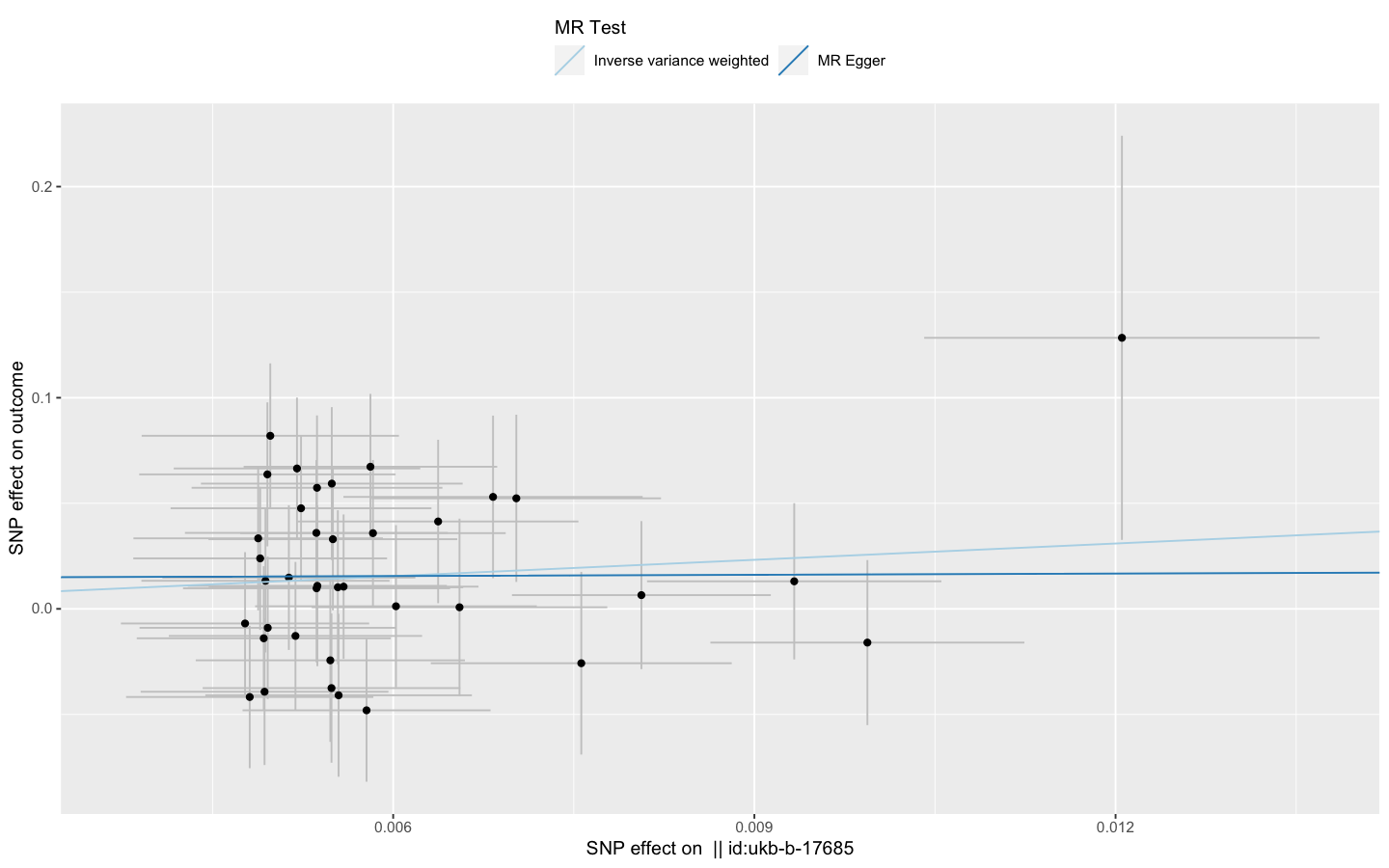


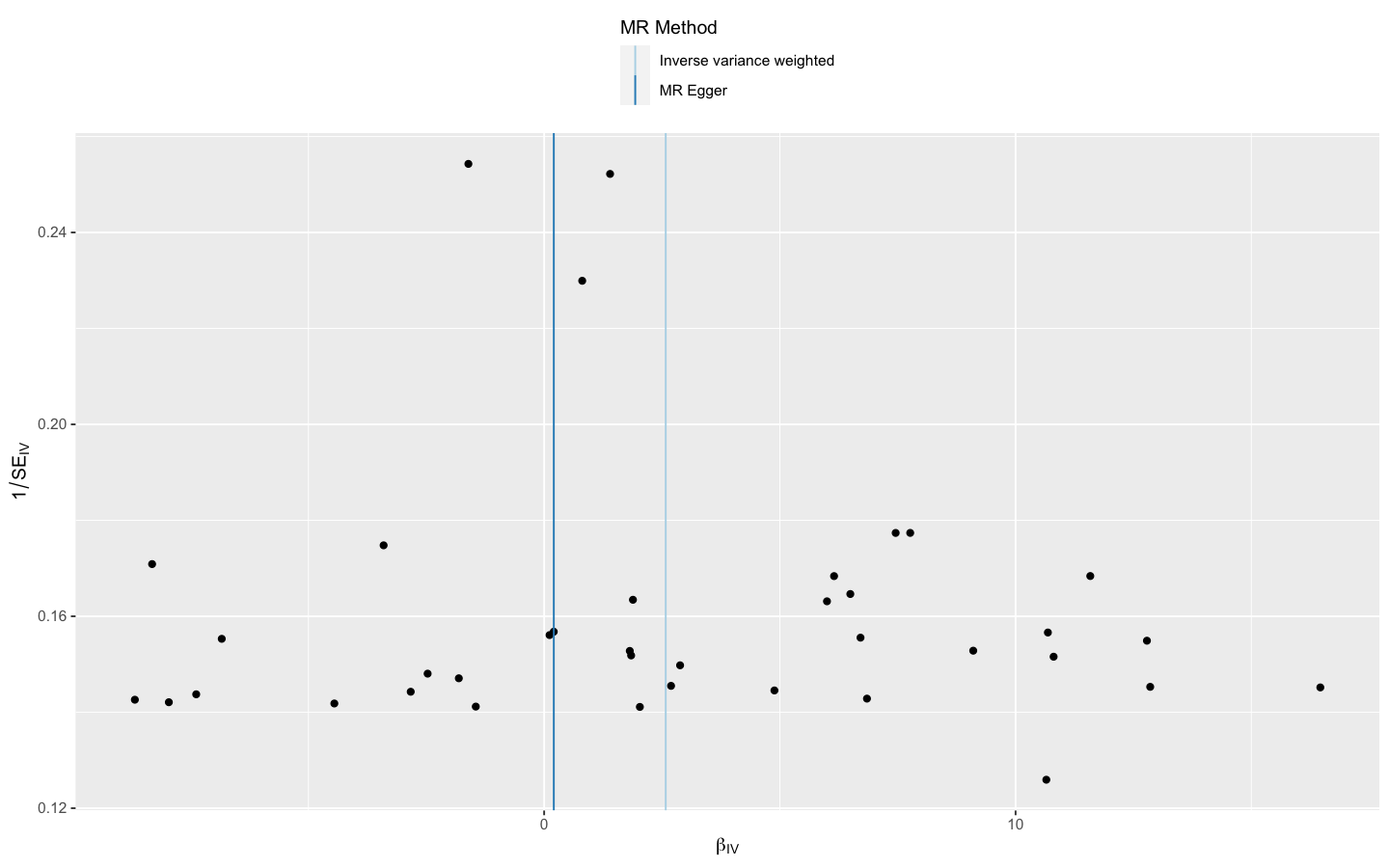


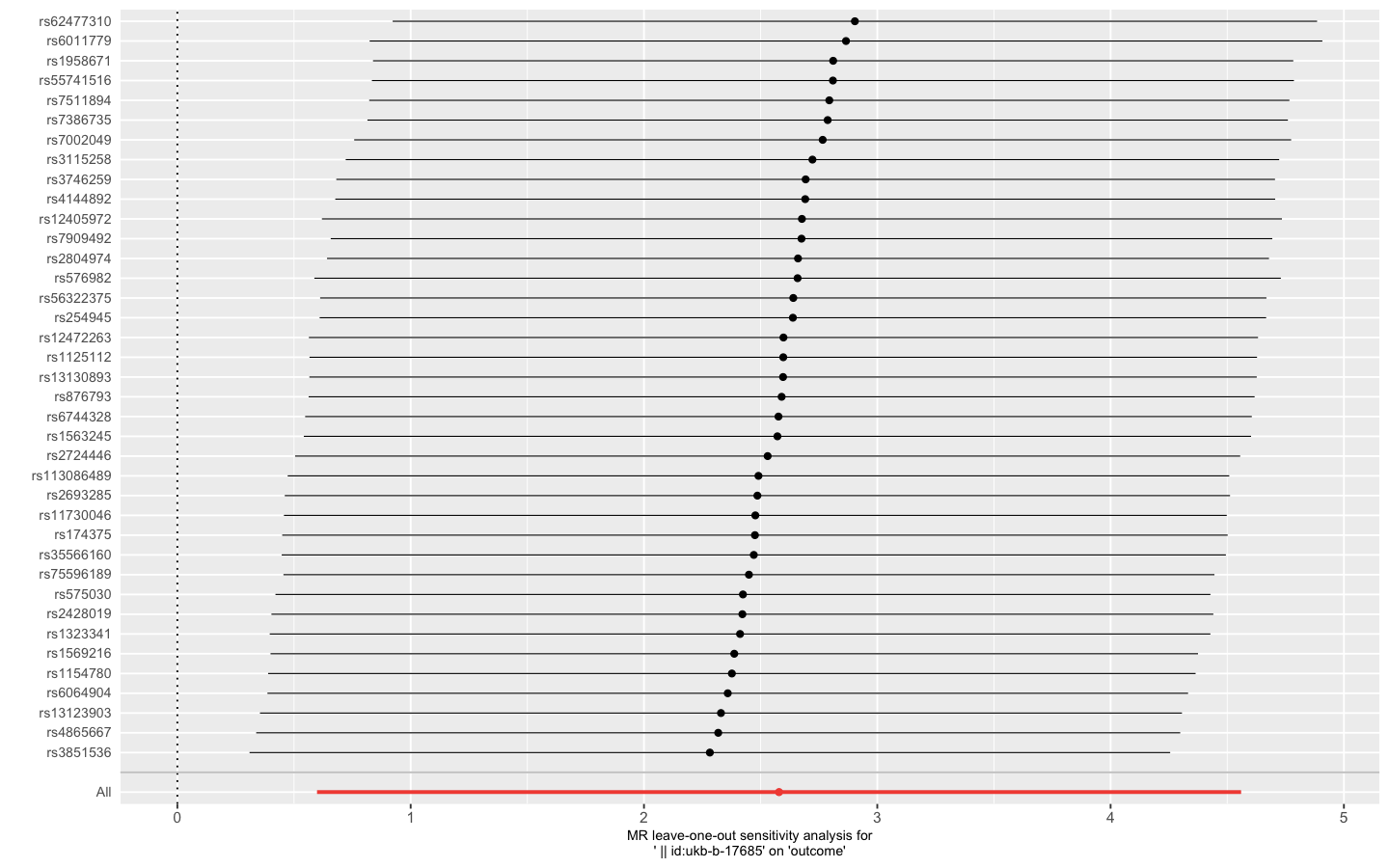


**Supplemental figure 6 Scatter, leave-one-out and funnel plots for effect of maternal smoking on idiopathic pulmonary fibrosis risk (excluding the SNPs associated with telomere length or obesity)**


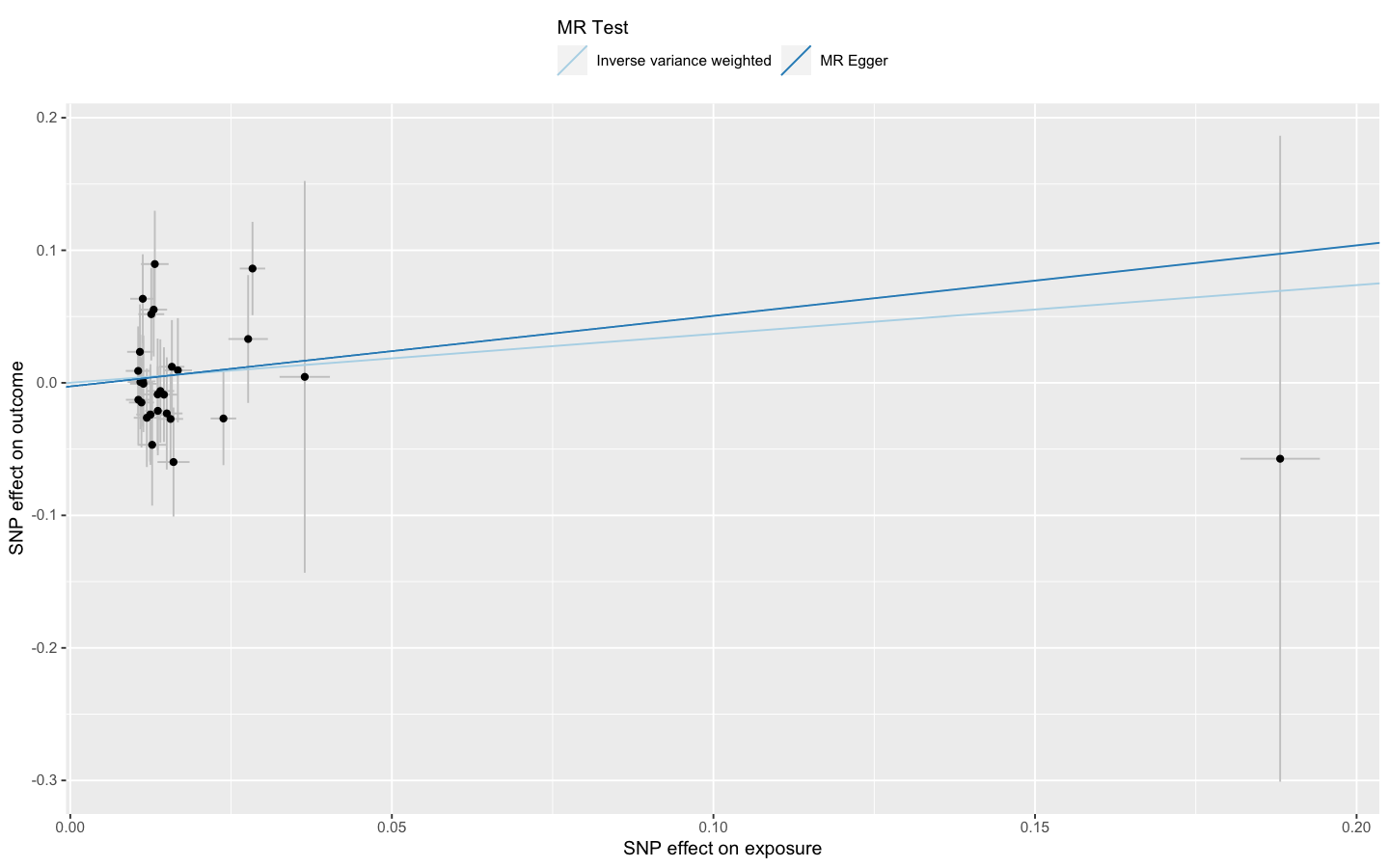


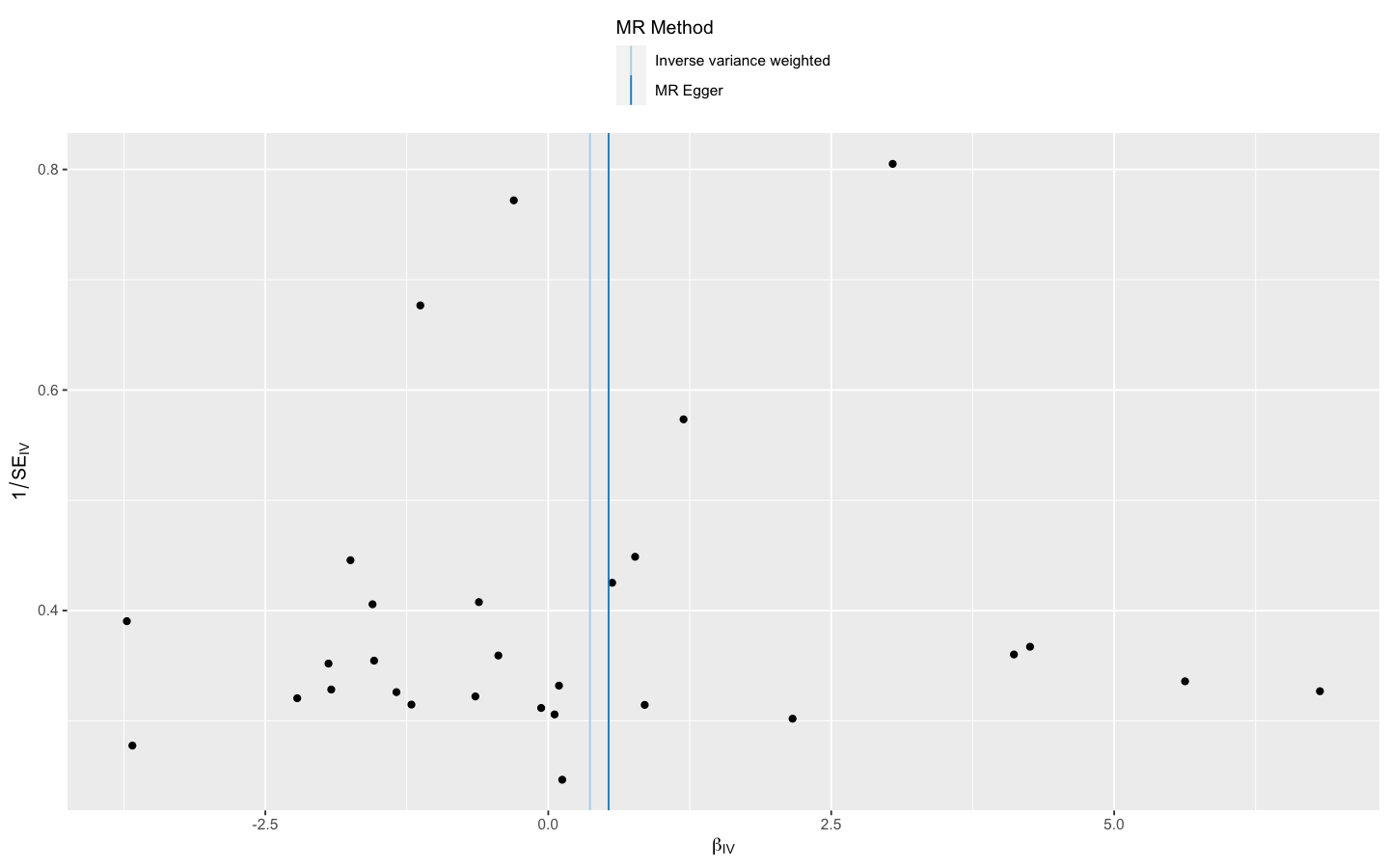


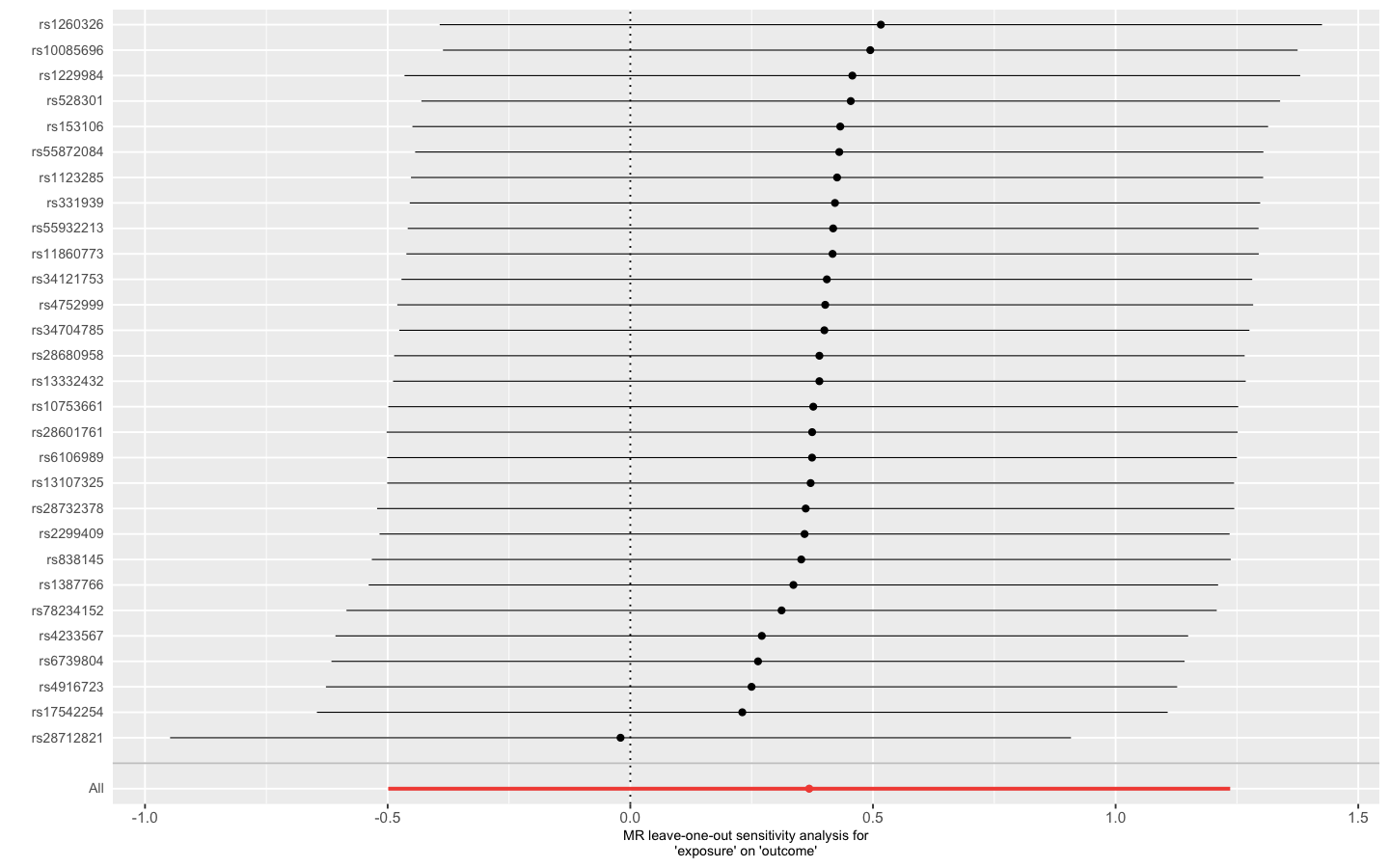


**Supplemental figure 7 Scatter, leave-one-out and funnel plots for effect of heavy drinking on idiopathic pulmonary fibrosis risk**


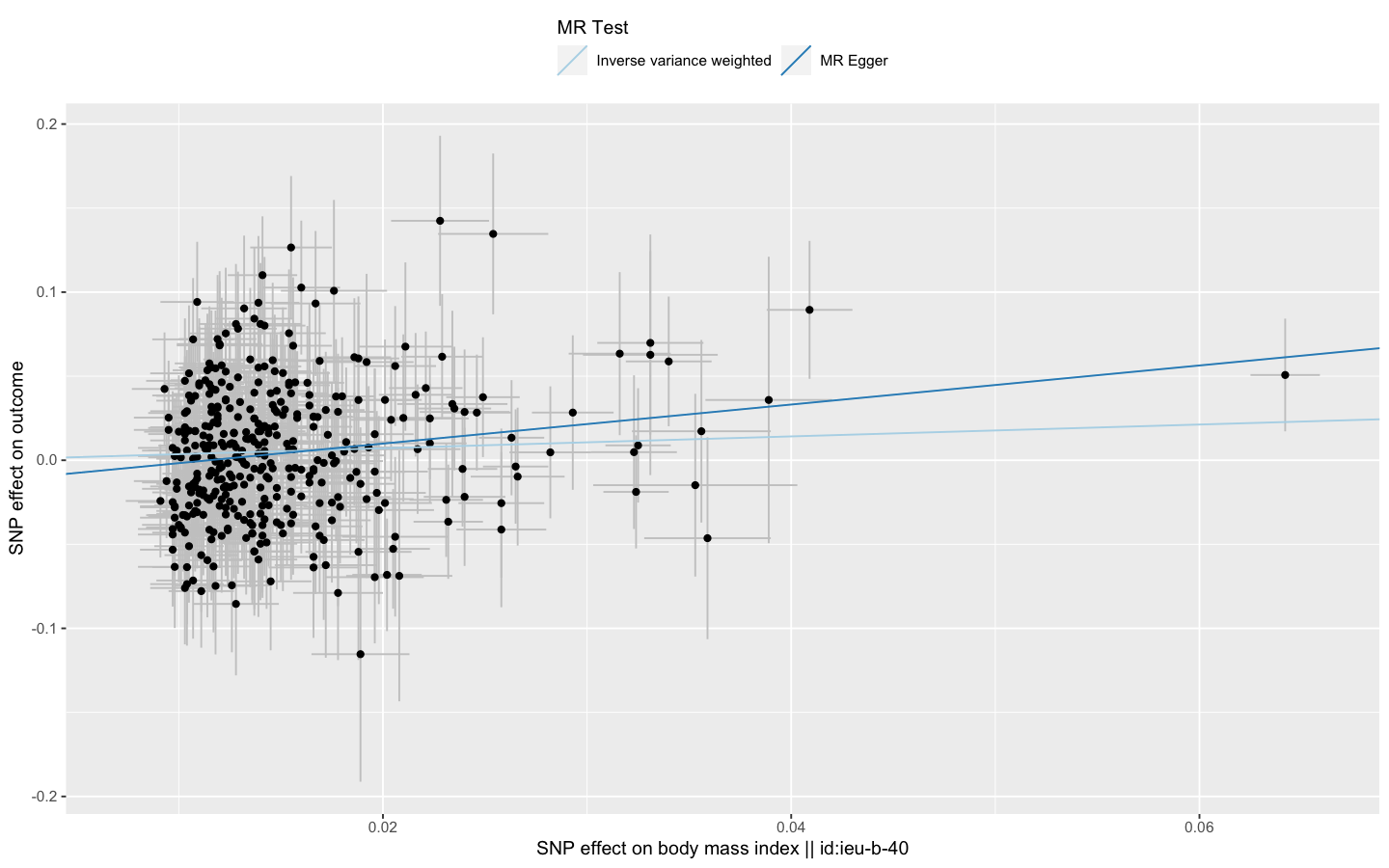


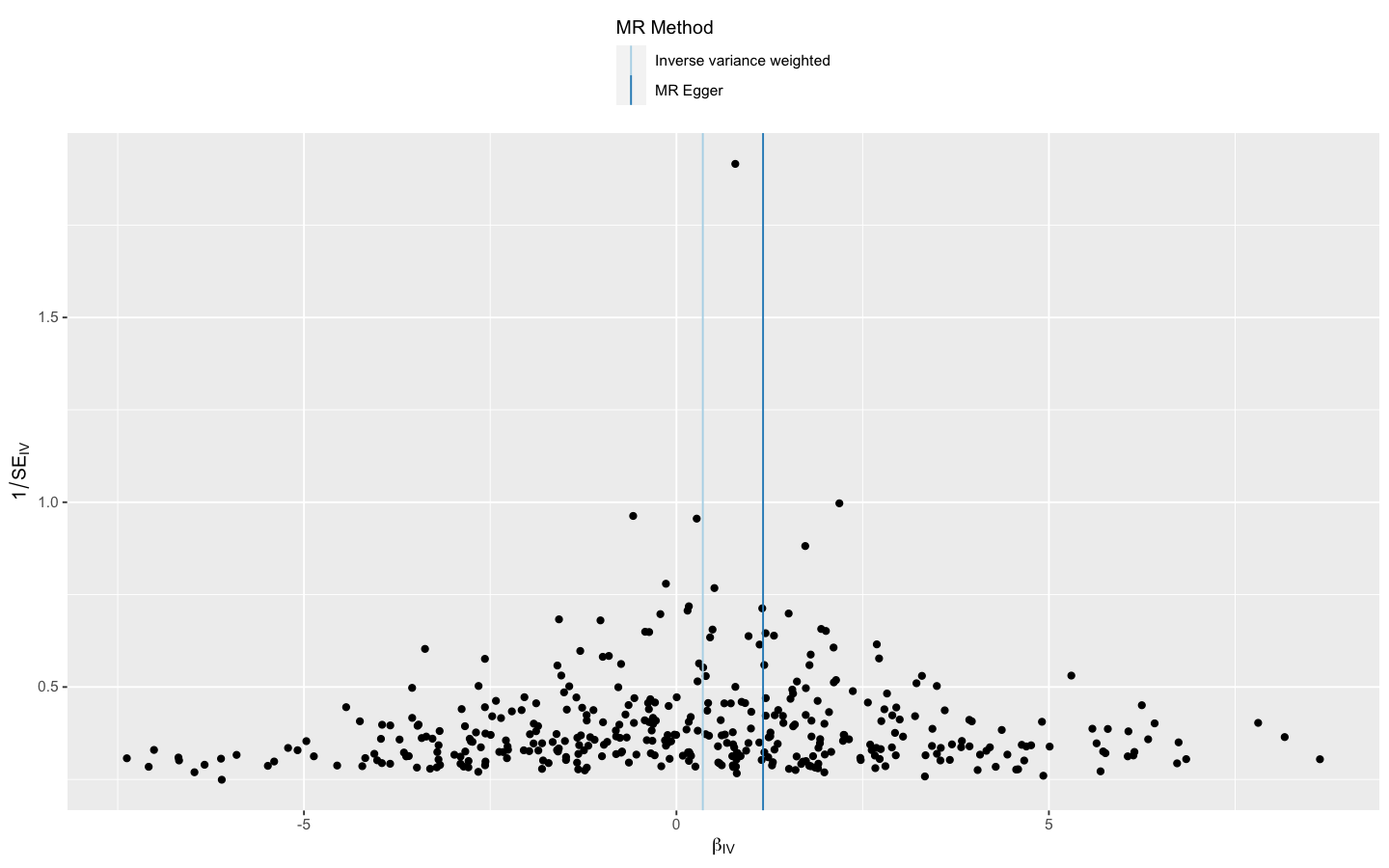


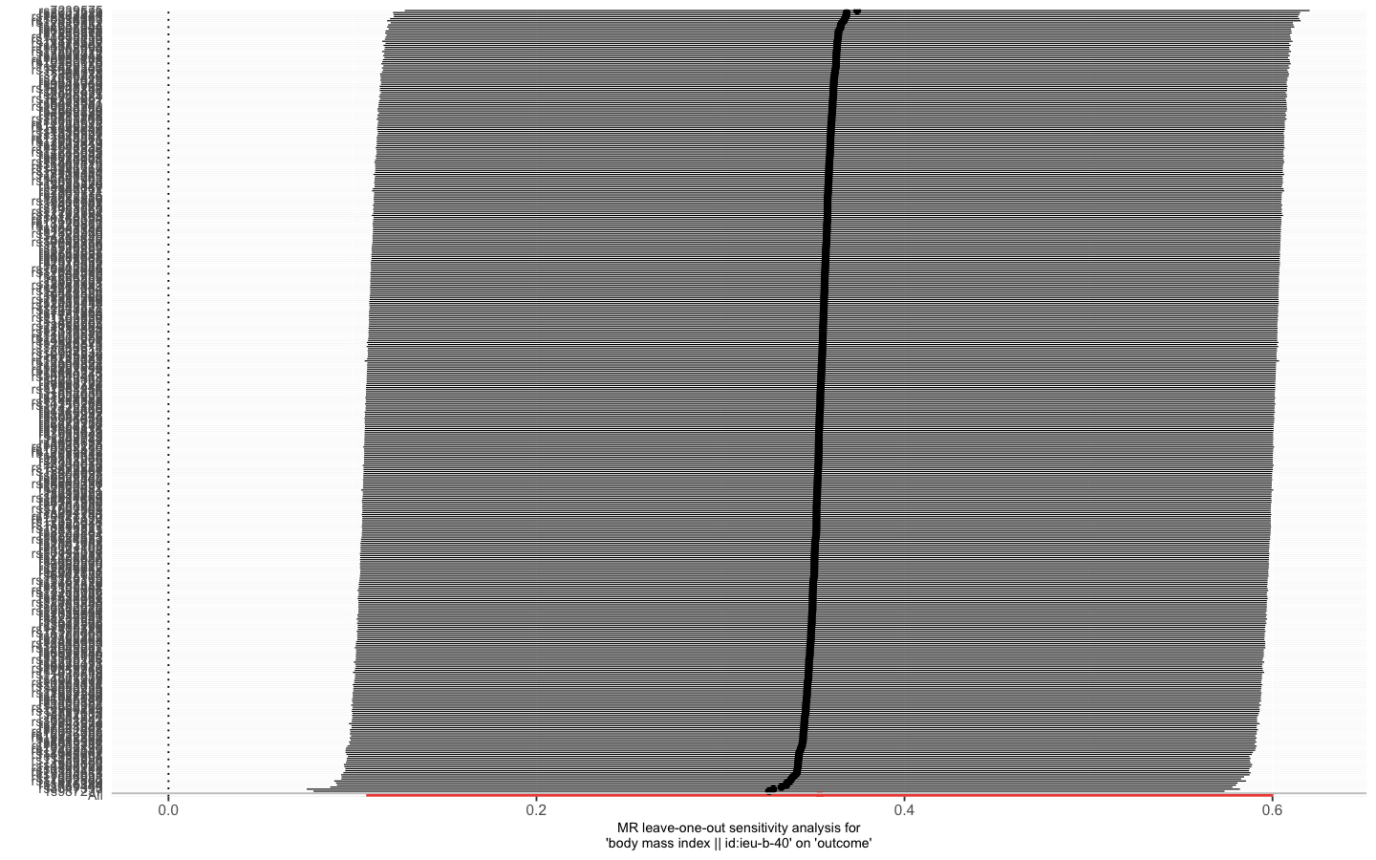


**Supplemental figure 8 Scatter, leave-one-out and funnel plots for effect of body mass index on idiopathic pulmonary fibrosis risk (excluding the SNPs associated with telomere length or smoking)**


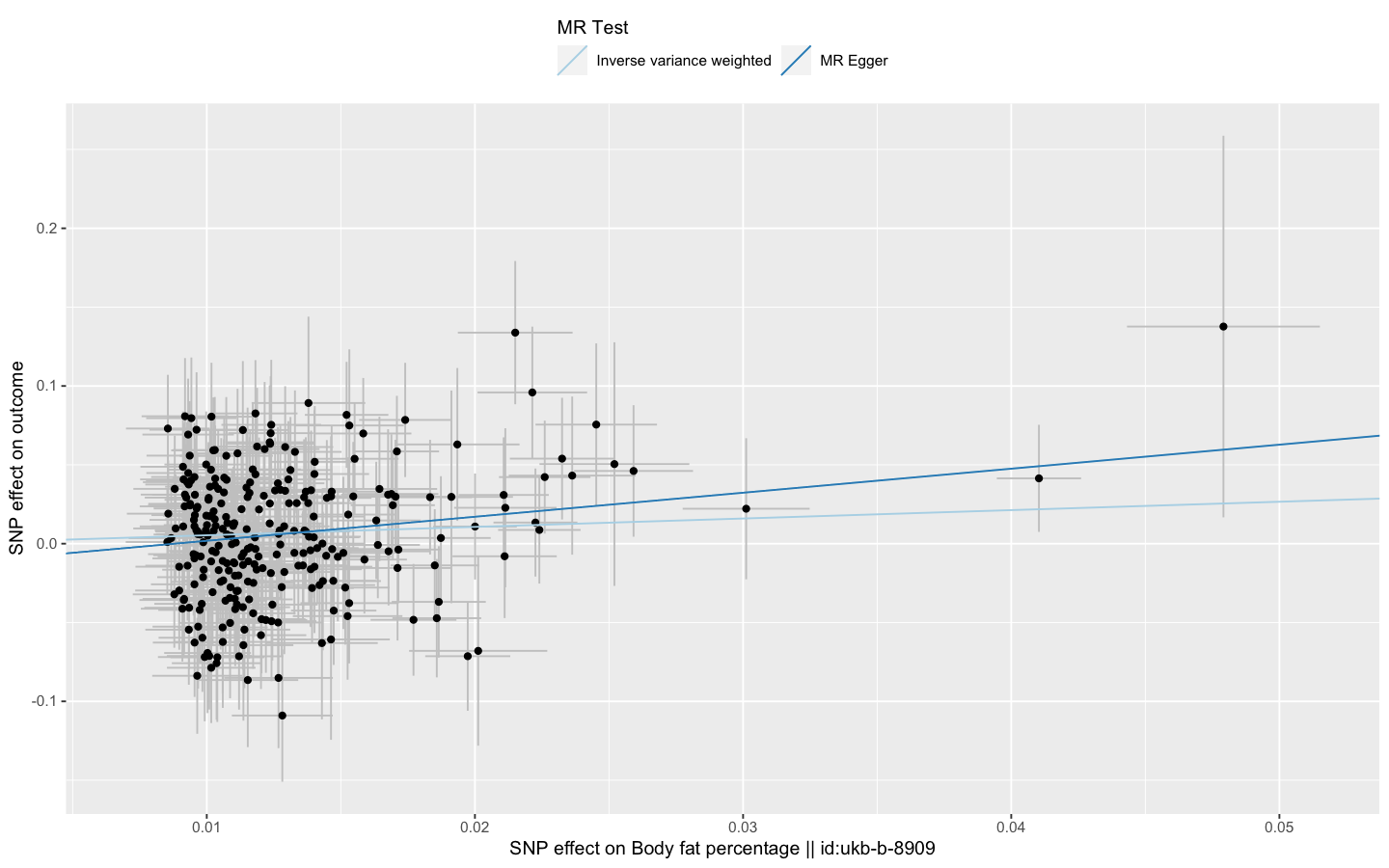


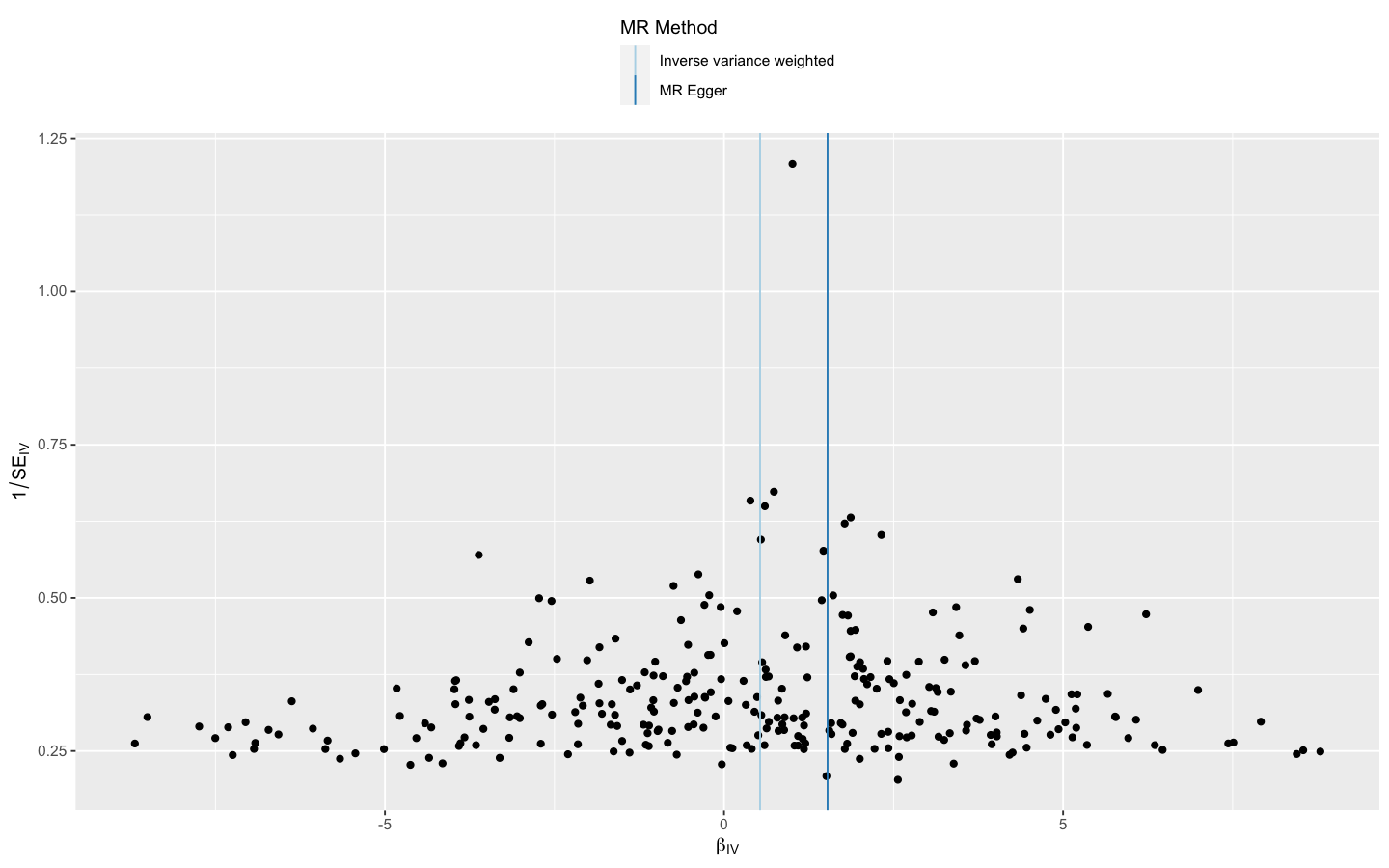


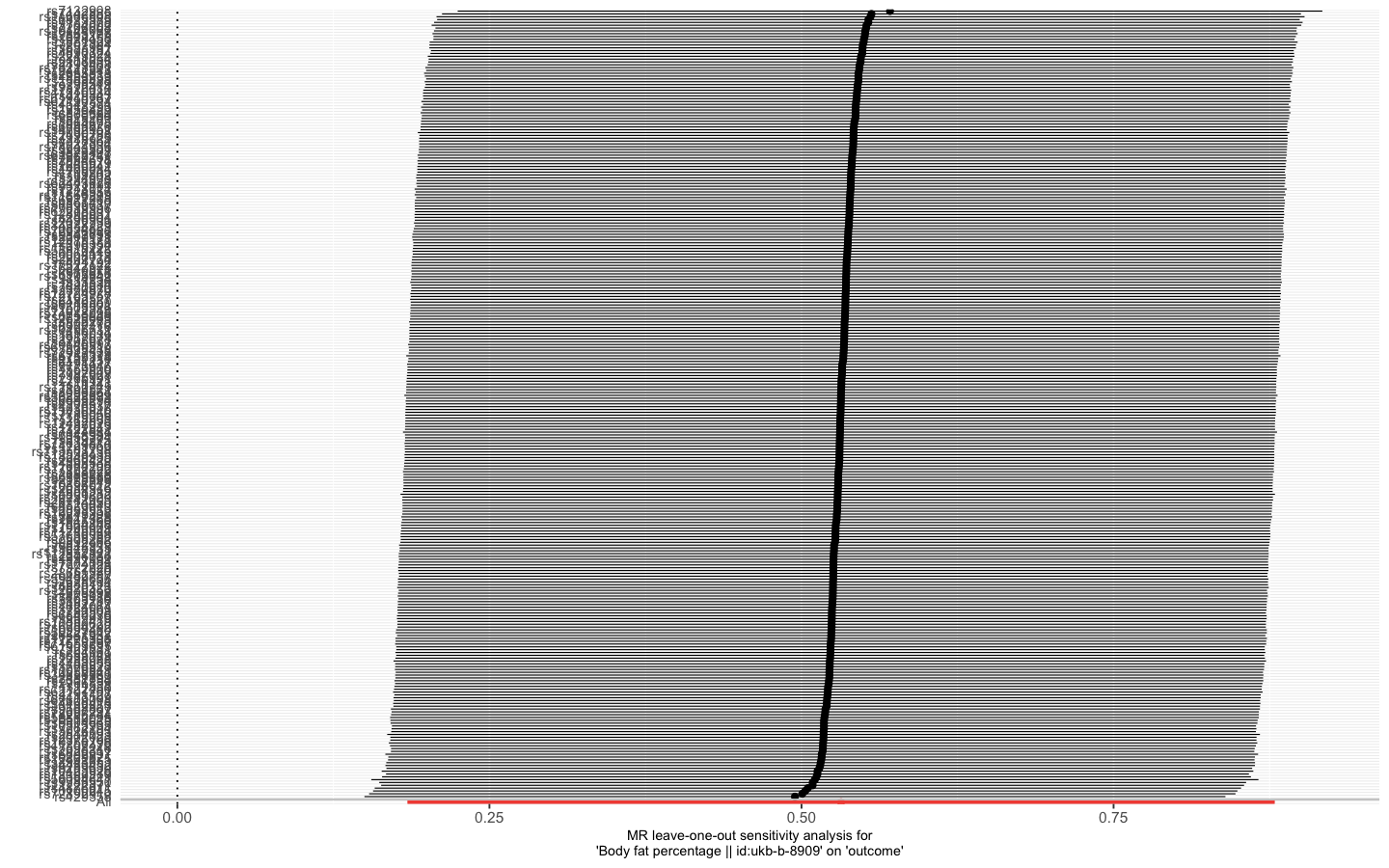


**Supplemental figure 9 Scatter, leave-one-out and funnel plots for effect of body fat percentage on idiopathic pulmonary fibrosis risk (excluding the SNPs associated with telomere length or smoking)**
